# Stable 1,3,2‐Benzodithiazolyl Radicals: Modification of Reactivity, Crystal Packing, and Solid State Magnetic Properties by Fluorination

**DOI:** 10.1002/open.202500561

**Published:** 2026-02-26

**Authors:** Alexander A. Buravlev, Alexander Yu. Makarov, Jordi Ribas‐Ariño, M. Àngels Carvajal, Mercè Deumal, Yaser Balmohammadi, Simon Grabowsky, Inna K. Shundrina, Boris A. Zakharov, Irina G. Irtegova, Mikhail N. Uvarov, Artem S. Bogomyakov, Irina Yu. Bagryanskaya, Leonid A. Shundrin, Andrey V. Zibarev

**Affiliations:** ^1^ Institute of Organic Chemistry Siberian Branch of the Russian Academy of Sciences Novosibirsk Russia; ^2^ Department of Natural Sciences National Research University – Novosibirsk State University Novosibirsk Russia; ^3^ Departament de Ciència de Materials i Química Física, and Institut de Química Teòrica i Computacional (IQTCUB) Universitat de Barcelona Barcelona Spain; ^4^ Department of Chemistry Biochemistry, and Pharmaceutical Sciences University of Bern Bern Switzerland; ^5^ Synchrotron Radiation Facility – Siberian Circular Source of Photons (SRF «SKIF») Novosibirsk Russia; ^6^ Institute of Chemical Kinetics and Combustion Siberian Branch of the Russian Academy of Sciences Novosibirsk Russia; ^7^ International Tomography Center Siberian Branch of the Russian Academy of Sciences Novosibirsk Russia

**Keywords:** 1,3,2‐benzodithiazolyls, fluoroorganics, magnetic properties, reactivity, structure

## Abstract

Impact of fluorination on crystal and molecular structure, heteroatom reactivity, and solid‐state magnetic properties of thermally‐stable *π*‐radicals is studied experimentally and computationally with 1,3,2‐benzodithiazolyl **1** ^**·**^ and its 4,7‐difluoro, 4,5,6,7‐tetrafluoro, and 4,7‐difluoro‐5,6‐(hexafluoropropane‐1,3‐diyl) derivatives **2** ^**·**^‐**4** ^**·**^, respectively. Radicals **2** ^**·**^‐**4** ^**·**^ are isolated by vacuum thermolysis of their unusual covalent 2:1 adducts with 7,7,8,8‐tetracyanoquinodimethane. The impact of fluorination on reactivity is evidenced by transformation of **2** ^**·**^‐**4** ^**·**^ and **2** ^**+**^‐**4** ^**+**^ into corresponding 2H‐1‐oxo‐1,3,2‐benzodithiazoles under the influence of air's or solvents’ moisture; back transformation into the cations under the action of protic acids; and formation of a paramagnetic molecular complex between **3** ^**·**^ and naphthalene, whereas **1** ^**·**^ and octafluoronaphthalene do not exhibit complexation. The crystal structures of **3** ^**·**^ and **4** ^**·**^ reveal a novel packing motif featuring radical pairs linked by four‐center interactions that stack into offset *π*‐columns, forming a unique zip‐*π*‐stack synthon that incorporates head‐over‐tail *π*‐pairs of radicals. Despite the formation of *π*‐pairs, polycrystalline **3** ^**·**^ and **4** ^**·**^ display a nonzero effective magnetic moment that rises with temperature above 200 K, although the values remain significantly lower than those of the high‐temperature polymorphs of magnetically‐bistable **1** ^**·**^ and **2** ^**·**^. This behavior can be rationalized by different magnetic topologies and values of spin exchange between the radicals.

## Graphical abstract

Fluorination reduces solid‐state thermal stability of 1,3,2‐benzodithiazolyl radicals and modulates crystal structures (e.g., by means of a new supramolecular zip‐synthon) and reactivity, in the latter case toward transformations unknown in the hydrocarbon series but potentially general in the fluorocarbon one. Low fluorination is favorable and high fluorination is unfavorable for hysteretic magnetic bistability of the radicals around room temperature.

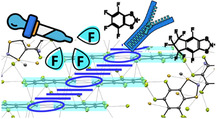

## 1 Introduction

**SCHEME 1:**
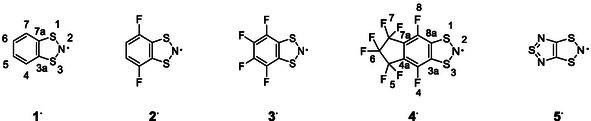
*π*‐Delocalized **R** ^**·**^s [21, 22] represented as N‐centered to avoid resonance superpositions, together with atom numbering.

Fluorine is a very special element exhibiting a unique impact on chemistry and materials science [1, 2]. Partial or complete fluorination is one of the most effective ways to modify chemical reactivity [3, 4, 5, 6]. Furthermore, the selective incorporation of fluorine into organic molecules has proven to be a powerful approach for tuning electronic and optoelectronic properties of organic materials, through electronic effects and/or modifications in crystal packing [2, 7, 8, 9, 10, 11, 12]. While the chemistry of fluorinated radicals is well‐studied [13, 14, 15, 16], the materials science of small, open‐shell fluorinated molecules has received relatively less attention [17, 18, 19, 20]—especially in comparison to other fluorinated closed‐shell organic functional materials. The fluorinated radicals reported so far include 1,3,2‐(aza)benzodithiazolyls (Wolmershäuser radicals **R** ^**·**^s and diradicals) [21, 22, 23], 1,2,3‐(aza)benzodithiazolyls (Herz radicals and diradicals) [24, 25], 1,2,3,4‐dithiadiazolyls [18, 20, 26, 27, 28], 1,2,4‐benzotriazinyls (Blatter radicals) [29, 30], (nitronyl) nitroxides [15, 31, 32], phenalenyls [33], and triarylmethyls [13, 34]. Despite the intensive study on aminyls (Rajca radicals) [35], fluorinated derivatives have not yet been reported, while fluorinated 1,2,3‐benzodithiazolyls (Herz radicals, isomers of **R** ^**·**^s) have only been detected by EPR and remain nonisolated [24]. The studies on fluorinated radicals have shown that the introduction of fluorine atoms can enhance radical stability and induce pronounced changes in macroscopic magnetic behavior by altering crystal packing. These effects are particularly valuable in the design of open‐shell molecular materials for spintronic devices, quantum information processing, and magnetic sensors [36, 37, 38, 39]. Despite these promising attributes, the number of structurally characterized fluorinated radicals remains limited, and their solid‐state magnetic properties are still underexplored. In this context, the present study investigates the synthesis, structure, and magnetic behavior of a series of fluorinated **R** ^**·**^s **1** ^**·**^‐**4** ^**·**^ providing new insights into how fluorination modulates radical reactivity and magnetic response through crystal engineering (Scheme 1, with **1** ^**·**^ = 1,3,2‐benzodithiazolyl; **2** ^**·**^ = 4,7‐difluoro‐1,3,2‐benzodithiazolyl; **3** ^**·**^ = 4,5,6,7‐tetrafluoro‐1,3,2‐benzodithiazolyl; **4** ^**·**^ = 4a,7a‐(hexafluoropropane‐1,3‐diyl)‐4,8‐difluoro‐1,3,2‐benzodithiazolyl; **5** ^**·**^ = 1,3,5‐trithia‐2,4,6‐triazapentalenyl).

The 1,3,2‐dithiazolyl family [21, 22, 23, 26, 40, 41, 42] represents a significant class of stable organic radicals, notable for providing several examples of bistable magnetic materials, i.e., systems that exhibit two polymorphic forms with distinct magnetic properties within a specific temperature range due to a hysteretic phase transition [21, 22, 23, 43, 44, 45, 46, 47, 48, 49, 50, 51, 52]. This phenomenon is particularly interesting when the bistability range encompasses room temperature (conventionally, 298 K) [21, 44]. Radicals **1** ^**·**^ [45, 46, 53, 54, 55, 56], **2** ^**·**^ [21], and their congener **5** ^**·**^ [44, 57, 58, 59, 60, 61, 62, 63, 64–65] (Scheme 1) feature low‐temperature (LT) diamagnetic and high‐temperature (HT) paramagnetic phases, i.e., magnetic bistability. Radical **5** ^**·**^ undergoes photo‐ [60, 61] and pressure‐ [65] induced phase transitions alongside with a thermally‐induced transition [44, 61]. It displays a broad‐loop hysteretic magnetic bistability (T↑ 320 K, T↓ 232 K) [44, 59], thus serving as reference compound for the whole **R** ^**·**^s family. However, **5** ^**·**^ is scarcely amenable to chemical modification by known methods, aside from its use as a ligand in metal coordination compounds [66]. Indeed, heavier‐chalcogen analogs of **5** ^**·**^, which are *a priori* hardly accessible if they exist at all [22], are unknown. In this context, **R** ^**·**^s appear more promising, as they are more prone to chemical modification (cf. **1** ^**·**^‐**4** ^**·**^, Scheme 1). In contrast to the low‐fluorinated derivative **2** ^**·**^ [21], only the low‐temperature (LT) phase of **1** ^**·**^ has been structurally resolved, while the structure of the high‐temperature (HT) phase revealed by magnetometry and powder EPR remained unknown [45, 46, 53, 54, 55, 56] before this work [67]. In this context, the potential of highly‐fluorinated **3** ^**·**^ and **4** ^**·**^ congeners of **1** ^**·**^ and **2** ^**·**^ is of obvious interest, and is here explored encompassing synthetic availability, crystal packing, solid‐state magnetic properties, and reactivity.

**FIGURE 1:**
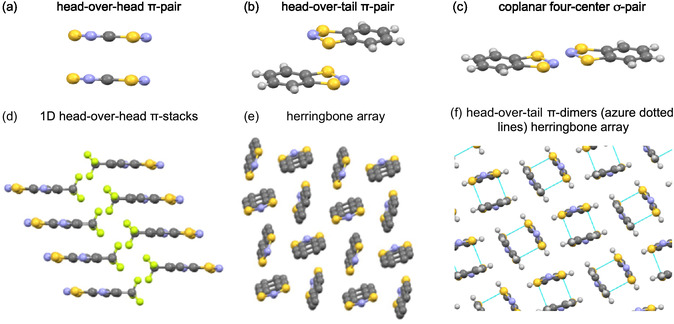
Building blocks and supramolecular synthons in crystal lattices of **R** ^**·**^s. Building blocks: (a) head‐over‐head *π*‐pair (**5** ^**·**^, CCDC 158385, 1255679) [58, 59], (b) head‐over‐tail *π*‐pair (**1** ^**·**^, CCDC 1151722) [46, 54, 55], and (c) coplanar four‐center *σ*‐pair (**1** ^**·**^, CCDC 1151722) [46, 54, 55]. Supramolecular synthons: (d) 1D head‐over‐head *π*‐stacks (CCDC 802275) [23], (e) herringbone array (CCDC 1252222) [72], and (f) head‐over‐tail *π*‐dimers (azure dotted lines) herringbone array (**1** ^**·**^, CCDC 1151722) [46, 54, 55] Color code: C, gray; H, light gray; F, light‐green; N, blue; S yellow.

In the context of highly‐correlated magnetic properties/crystal packing, supramolecular synthons [68, 69], i.e., crystal packing motifs that can be used as patterns to design materials, must be first disclosed. The **R** ^**·**^s are planar [21, 22], which can result in solid‐state building blocks displaying: 1) head‐over‐head *π*‐pairs, 2) head‐over‐tail *π*‐pairs, and 3) coplanar four‐center *σ*‐pairs (Figure 1). The *π*‐pairs exhibit different degrees of longitudinal/latitudinal overlap ranging between perfectly eclipsed and staggered situations. Additionally, in the crystalline state, they are involved in lateral secondary bonding interactions (SBIs) [70, 71]. The building blocks combine and lead to supramolecular synthons, such as 1) 1D head‐over‐head *π*‐stacks of **R** ^**·**^s, i.e., lamellar array, which can be either regularly spaced or dimerized [23]; 2) herringbone array of **R** ^**·**^s [72]; and 3) head‐over‐tail sandwich herringbone array with **R** ^**·**^s dimeric units in a parallel‐planar, centrosymmetric, staggered geometry, giving rise to 2D sheets of weakly linked S atoms [54, 55] (Figure 1). The most commonly encountered synthon consists of slipped *π*‐stacks of **R** ^**·**^s [21, 45, 73, 74, 75], whereas the coplanar four‐center *σ*‐pairs are exhibited by **R** ^**·**^s in metal coordination compounds [53]. In all cases, these synthons are then held together by SBIs to arrange into the final 3D crystal packing. Notably, the **R** ^**·**^‐based materials with different synthons share the same magnetic response, e.g., magnetic bistability, paramagnetism, etc. [21, 22, 23, 45, 54, 55, 72, 73, 74, 75]. Therefore, the magnetic behavior of the **R** ^**·**^‐based crystals is apparently not critically dependent on the synthons the **R** ^**·**^s exhibit in the crystal packings.

For **1** ^**·**^ and **2** ^**·**^, the synthons in the crystal packing and the magnetic response are summarized as follows. Crystalline **1** ^**·**^ consists of centrosymmetric dimers, where the dimer units form a 2D herringbone‐like network (Figure 1) [46, 54, 55]. While the bulk crystals of **1** ^**·**^ are diamagnetic due to this dimerization at room temperature, they exhibit a phase transition to a paramagnetic solid phase at 346 K (**1** ^**·**^‐HT) and a superheating process that results in the double melting (melt → recrystallization → melt process) around 360–365 K. Supercooling of the paramagnetic **1** ^**·**^‐HT leads to an antiferromagnetic (AFM) ordering at 11 K [54].

For bistable **2** ^**·**^ [21], as for other bistable **R** ^**·**^s with known crystal structures, the LT phase (296 K) is composed of dimerized (···**R** ^**·**^−**R** ^**·**^···)_*n*_ *π*‐stacks and the HT phase (328 K) of equidistant (···**R** ^**·**^···**R** ^**·**^···)_*n*_ *π*‐stacks. These equidistant *π*‐stacks are not potential energy surface minima but average structures arising from a dynamic interconversion between two degenerate (···**R** ^**·**^−**R** ^**·**^···**R** ^**·**^−**R** ^**·**^···)_*n/*__2_ ↔ (−**R** ^**·**^···**R** ^**·**^−**R** ^**·**^···**R** ^**·**^−)_*n/*__2_ configurations. Both phases show head‐over‐head *π*‐stacks of nearly eclipsed **R** ^**·**^s as a synthon (Figure 1). It must be stressed that temperature‐dependent intra‐stack dynamics give rise to a second‐order phase transition changing the dominant magnetic interactions. However, this dynamics itself is insufficient to generate magnetic bistability. It additionally requires rearrangements of lateral SBIs/*σ*‐contacts between the *π*‐stacks via a first‐order phase transition [21, 50, 57, 62, 63, 64]. In the case of **2** ^**·**^, H···F hydrogen bonds [76, 77, 78, 79, 80], impossible for **1** ^**·**^, **3** ^**·**^, and **4** ^**·**^, were supposed to be crucial, which are shortened as compared with the corresponding sum of van der Waals (VdW) radii [21].

In this work, structural and magnetic properties of **1** ^**·**^ [46, 54, 55], **2** ^**·**^ [21], and newly synthesized **3** ^**·**^ and **4** ^**·**^ are compared using single‐crystal and powder X‐ray diffraction (XRD), solution and variable‐temperature powder electron paramagnetic resonance (EPR), simultaneous thermogravimetry–differential scanning calorimetry (TG‐DSC), and superconducting quantum interference device (SQUID) magnetometry. Redox properties of **3** ^**+**^ and **4** ^**+**^ are studied by cyclic voltammetry (CV). Theoretical calculations are used to identify the synthons of **1** ^**·**^‐**4** ^**·**^, and crucial synthon···synthon contacts/SBIs, aiming at indicating relative stabilities among observed synthons. Alongside with the synthon approach [68, 69] operating with molecular ensembles, Hirshfeld surface analyses [81, 82] of **1** ^**·**^‐**4** ^**·**^ are conducted. Finally, the crystal packing of **1** ^**·**^‐**4** ^**·**^ is further inspected to interpret their magnetic response. These approaches are each other complementary and jointly provide a more complete picture at the microscopic level of **1** ^**·**^‐**4** ^**·**^ [83, 84].

In the reactivity context, reactions unknown in the hydrocarbon series but, expectedly, possessing a general character in the fluorocarbon one, are found. They embrace the formation of 2:1 covalent adducts of **2** ^**·**^‐**4** ^**·**^ with 7,7,8,8‐tetracyanoquinodimethane (TCNQ); the 1:1 complexation of **3** ^**·**^ with naphthalene, whereas **1** ^**·**^ and octafluoronaphthalene do not form complex; reactions of **2** ^**·**^‐**4** ^**·**^ and **2** ^**+**^‐**4** ^**+**^ with H_2_O transforming them into 2H‐1‐oxo‐1,3,2‐benzodithiazoles, whereas **1** ^**·**^ is stable in aqueous solutions. Reactivity is therefore introduced as an element to highlight the Lewis ambiphilic character and potential for supramolecular engineering of **3** ^**·**^ and **4** ^**·**^.

## 2 Experimental and Computational Section

### 2.1 General

Starting materials were sourced from Macklin and Sigma Aldrich except 3,4,5,6‐tetrafuorobenzene‐1,2‐bis(sulfenyl chloride) [85], for which a new synthetic protocol was elaborated (see Section S1). Compounds **1** ^**+**^Cl^–^, **2** ^**+**^Cl^–^, and **1** ^**·**^ were synthesized by published methods [21, 86]. For preparation of, and manipulation with, **1** ^**+**^Cl^–^‐**4** ^**+**^Cl^–^, **1** ^**·**^‐**4** ^**·**^, and **6**‐**8**, glovebox, Schlenk, and vacuum‐line techniques were used.

Elemental analyses for C, H, N, and S were accomplished with Euro EA 3000 and Carlo Erba 1106 analyzers; for S also by the manual combustion method and for F by spectrophotometric method [87]. High‐resolution MS (EI, 70 eV) and ESI‐MS spectra were measured with Thermo Electron DFS and Bruker micrOTOF‐Q instruments, respectively, using for ESI‐MS calibrations an arginine solution in double distilled water acidified with formic acid (see Section S3). ^1^H (300.1 MHz) and ^19^F (282.4 MHz) NMR spectra were recorded on a Bruker AV‐300 spectrometer, with δ chemical shifts referenced to TMS and C_6_F_6_ (δ^19^F = –162.9 ppm with respect to CFCl_3_) (see Section S4). IR and attenuated total reflectance (ATR) IR spectra were collected with Bruker Tensor 27 and Varian 640‐IR instruments using a diamond as ATR crystal (see Section S5). UV–vis spectra were obtained using an HP 8453 spectrophotometer (see Section S6). Simultaneous TG‐DSC measurements were carried out under helium flow using a Netzsch STA 409 instrument equipped with platinum pan. Temperature and heat flow calibration was performed according to ISO 11357–1 standard using Netzsch calibration set; data were analyzed using *Netzsch Proteus Thermal Analysis* software (see Section S7).

### 2.2 Syntheses

#### 2.2.1 Salts 3^+^Cl^–^ and 4^+^Cl^–^

At 0°C and under argon, a solution of 0.57 g (2 mmol) of Me_3_SiN_3_ in 5 mL of CH_2_Cl_2_ was added to a solution of 0.27 mL (2 mmol) of 3,4,5,6‐tetrafuorobenzene‐1,2‐bis(sulfenylchloride) in 12 mL of the same solvent. After 1 h, the solvents were distilled off, 3 mL of pentane were added to the residue, and the solid filtered off. 4,5,6,7‐Tetrafluoro‐1,3,2‐benzodithiazolium chloride **3** ^**+**^Cl^–^ (CCDC 731911) [88] (0.453 g, 86%) was obtained in the form of orange powder, m.p. 103°C (decomp.). Found/calculated for C_6_ClF_4_NS_2_: C 27.88/27.54, Cl 12.21/13.55, F 29.19/29.05, N 5.39/5.35, S 24.54/24.51. MS, M^+^, m/z, measured/calculated for C_6_F_4_N^32^S_2_: 225.9401/225.9403. ^19^F NMR (CDCl_3_), δ, ppm: 33.4 (m, 2F), 16.7 (m, 2F); (CDCl_3_/CF_3_CO_2_H): 37.5 (m, 2F), 28.9 (m, 2F). UV–vis (CH_2_Cl_2_), *λ*_max_, nm/log *ε*: 231/3.95, 302/3.46, 388/3.39. ATR IR, *v*, cm^–1^: 3143 w, 3051 w, 2611 w, 1871 w, 1847 w, 1778 w, 1720 w, 1685 w, 1635 m, 1608 m, 1468 vs, 1442 vs, 1334 s, 1281 vs, 1132 vs, 1038 vs, 984 s, 903 m, 682 s, 822 s, 727 s, 633 m, 565 s, 484 m.

At ambient temperature and with CaCl_2_‐tube protection, 10 mL (≈124 mmol) of SO_2_Cl_2_ were added to 5.00 g (≈10 mmol) of 5,6‐bis(benzylthio)octafluroindane [89], the reaction mixture was kept overnight, and excess of SO_2_Cl_2_ was distilled off. Under argon, the residue was dissolved in 125 mL of CH_2_Cl_2_ and a solution of 1.31 mL (≈10 mmol) of Me_3_SiN_3_ in 25 mL of the same solvent was added at 0°C. After 1 h, the solvents were distilled off, 30 mL of pentane were added, and the solid filtered off. 4,7‐Difluoro‐5,6‐(hexafluoropropane‐1,3‐diyl)‐1,3,2‐benzodithiazolium chloride **4** ^**+**^Cl^–^ (3.21 g, 87%) was obtained in the form of red powder, m.p. 60°C (decomp.). Found/calculated for C_9_ClF_8_NS_2_: C 29.26/28.93, Cl 9.30/9.49, F 40.30/40.67, N 3.71/3.75, S 17.05/17.16. MS, M^+^, m/z, measured/calculated for C_9_F_8_N^32^S_2_: 337.9340/372.9039. ^19^F NMR (CDCl_3_), δ, ppm: 54.0 (s, 4F), 53.6 (s, 2F), 32.0 (s, 2F); (CDCl_3_/CF_3_CO_2_H): 60.0 (s, 2F), 55.0 (s, 4F), 32.9 (s, 2F). UV–vis (CH_2_Cl_2_), *λ*_max_, nm/log *ε*: 227/3.99, 237/3.91, 268/3.73, 321/3.57, 386/2.84. ATR IR, *v*, m^–1^: 2642 w, 1809 w, 1705 w, 1645 m, 1572  w, 1450 vs, 1423 vs, 1302 vs, 1248 vs, 1153 vs, 1099 vs, 978 vs, 951 vs, 897 s, 829 m, 762 s, 708 s, 667 m, 575 s, 548 vs, 440 s.

#### 2.2.2 Attempted Isolation of 3^·^ and 4^·^ From Reaction Solutions

At ambient temperature and under argon, **3** ^+^Cl^–^ or **4** ^+^Cl^–^ (≈0.45 mmol) in CH_2_Cl_2_ or CH_3_CN (10 mL) were reduced into **3** ^·^ or **4** ^·^, respectively (EPR), with Ph_3_Sb, or [Na(15‐crown‐5)]_2_[S_2_O_4_], or Cu or Ag powder, or Et_4_N^+^I^–^. The solvent was distilled off and the residue heated at ≈65°C/0.25 Torr in a vacuum sublimation apparatus. In most cases, however, no sublimation was observed. Specifically, **4** ^+^Cl^–^ and Ph_3_Sb produced only a minor EPR‐active sublimate that could be dissolved in CH_2_Cl_2_; however, upon cooling to –35°C, only dibenzotetrathiocine (XRD, CCDC 2347752) [89] crystallized. For **3** ^+^Cl^–^ and [Na(15‐crown‐5)]_2_[S_2_O_4_], chromatography (silica column/CH_2_Cl_2_) of the residue afforded dibenzotetrathiocine (^19^F NMR) [89, 90].

#### 2.2.3 Adducts of R^·^s and TCNQ

At ambient temperature and under argon, 1.46 mmol of **2** ^**+**^Cl^–^, **3** ^**+**^Cl^–^, or **4** ^**+**^Cl^–^ in 50 mL of CH_2_Cl_2_ were reduced into **2** ^**·**^‐**4** ^**·**^, respectively (EPR), with 1.83 mmol (0.116 g) of Cu powder. After 1 h, the reaction mixtures were filtered, 0.138 g (0.678 mmol) of TCNQ added to the filtrates, and the solvents distilled off. The residues were stirred with 2 mL of CH_3_CN, and the solids were filtered off: 1,4‐bis(4,7‐difluoro‐1,3,2‐benzodithiazol‐2‐yldicyanomethyl)benzene **6** (77%), or 1,4‐bis(4,5,6,7‐tetrafluoro‐1,3,2‐benzodithiazol‐2‐yldicyanomethyl)benzene **7** (74%), or 1,4‐bis(4,5,5,6,6,7,7,8‐octafluoroindano[5,6‐*d*][1,3,2]dithiazol‐2‐yldicyanomethyl)benzene **8** (71%), respectively, were obtained in the form of yellowish powders.

Adduct **6**, decomposition in ≈126°C–147°C range. Found/calculated for C_24_H_8_F_4_N_6_S_4_: C 49.69/49.31, H 1.44/1.38, F 13.04/13.00, N 14.67/14.38, S 21.14/21.94. UV–vis (CH_2_Cl_2_), *λ*_max_, nm/log *ε*: 259/4.50, 294/3.77, 379/4.70, 400/4.89. ATR IR, *v*, cm^–1^: 3090 w, 3051 w, 2486 w, 2418 w, 2243 w, 2224 w, 2102 w, 1924 w, 1875 w, 1799 w, 1751 w, 1656 w, 1639 w, 1585 w, 1543 w, 1512 w, 1471 s, 1418 m, 1373 w, 1298 w, 1282 w, 1261 w, 1230 s, 1200 m, 1153 w, 1119 m, 1080 w, 1022 w, 985 m, 951 m, 897 w, 843 m, 822 s, 731 m, 706 m, 667 w, 598 w, 575 w, 555 w, 544 w, 488 w, 474 w, 426 w, 405 w. Single crystals suitable for XRD were obtained from CH_2_Cl_2_ solution at –35°C.

Adduct **7**, decomposition in ≈110°C–124°C range. Found/calculated for C_24_H_4_F_8_N_6_S_4_: C 43.90/43.90, H 0.98/0.61, F 21.83/23.15, N 12.80/12.80, S 19.53/19.35. UV–vis (CH_2_Cl_2_), *λ*_max_, nm/log *ε*: 252/4.31, 290/3.76, 378/4.71, 400/4.91. IR (KBr), *v*, cm^–1^: 3049 w, 2243 w, 2224 w, 1990 w, 1927 w, 1797 w, 1543 w, 1479s, 1416 m, 1338 w, 1276 w, 1201 w, 1120 m, 1103 m, 1078 m, 1062 w, 1038 s, 984 m, 910 w, 866 m, 839 s, 817 m, 744 w, 737 w, 669 w, 629 w, 596 w, 571 w, 546 w, 507 w, 474 w, 431 w.

Adduct **8**, decomposition in ≈111°C–166°C range. Found/calculated for C_30_H_4_F_16_N_6_S_4_: C 41.58/40.92, H 0.74/0.46, F 32.70/34.52, N 10.15/9.54, S 14.61/14.56. UV–vis (CH_2_Cl_2_), *λ*_max_, nm/log *ε*: 262/4.44, 286/3.76, 379/4.55, 400/4.75. IR (KBr), *v*, cm^–1^: 3049 w, 2245 w, 2224 w, 1992 w, 1927 w, 1799 w, 1620 w, 1605 w, 1543 w, 1473 s, 1429 s, 1331 s, 1302 s, 1250 s, 1201 s, 1161 s, 1103 s, 1055 m, 984 m, 964 m, 947 m, 922 m, 877 s, 864 m, 818 m, 741 w, 704 w, 690 w, 667 w, 590 m, 579 m, 548 m, 474 w, 442 w, 424 w.

Molecular ions **6** ^+^‐**8** ^+^ were not observed by either MS or ESI‐MS. In ESI‐MS, [TCNQ]^·–^ and **2** ^**+**^‐**4** ^**+**^ were detected (see Section S3).

#### 2.2.4 Radicals 2^·^‐4^·^

In a vacuum sublimation apparatus, 0.13 mmol of **6**, **7**, or **8** were heated under 0.5 Torr at 75°C, affording **2** ^**·**^ (78%), **3** ^**·**^ (68%), or **4** ^**·**^ (75%), respectively, as sublimated black crystals suitable for XRD.

Radical **2** ^**·**^ [21], UV–vis (CH_2_Cl_2_), *λ*_max_, nm/log *ε*: 223/3.55, 259/4.17, 292/3.40, 317/3.00, 378/3.25, 398/3.30.

Radical **3** ^**·**^, found/calculated for C_6_F_4_NS_2_: C 32.01/31.86, F 33.16/33.60, N 6.77/6.19, S 27.85/28.35. MS, M^+^, m/z, measured/calculated for C_6_F_4_N^32^S_2_: 225.9405/225.9403. UV–vis (CH_2_Cl_2_), *λ*_max_, nm/log *ε*: 253/3.97, 290/3.45, 318/3.30, 390/3.30. ATR IR, *v*, cm^–1^: 1626 w, 1585 w, 1460 vs, 1298 s, 1271 s, 1109 s, 1028 vs, 881 vs, 746 s, 681 s, 627 m, 525 m, 442 w.

Radical **4** ^**·**^, found/calculated for C_6_H_2_F_2_NS_2_: C 32.17/31.96, F 44.96/44.94, N 4.05/4.14, S 18.91/18.96. MS, M^+^, m/z, measured/calculated for C_9_F_8_N^32^S_2_: 337.9340/337.9339. UV–vis (CH_2_Cl_2_), *λ*_max_, nm/log *ε*: 226/3.63, 262/4.33, 399/3.40. ATR IR, *v*, cm^–1^: 1628 w, 1581 w, 1462 s, 1421 s, 1306 s, 1238 s, 1198 s, 1144 vs, 1090 vs, 1063 s, 941 vs, 874 s, 820 m, 706 m, 679 s, 577 s, 550 s, 523 s, 569 w, 428 m.

#### 2.2.5 Hydrolysis of 2^·^‐4^·^

At 4°C, 0.15 mmol of **2** ^**·**^, **3** ^**·**^, or **4** ^**·**^ were exposed to air for 72 h. This yielded 2H‐1‐oxo‐4,7‐difluoro‐1,3,2‐benzothiadiazole (**9**, 89%), 2H‐1‐oxo‐4,5,6,7‐tetrafluoro‐1,3,2‐benzothiadiazole (**10**, 94%), or 2H‐1‐oxo‐4,7‐difluoro‐5,6‐(hexafluoropropane‐1,3‐diyl)‐1,3,2‐benzodithiazole (**11**, ≈100%), respectively, as light‐gray powders.

Compound **9**, m.p. 101°C–103°C (decomp.). Found/calculated for C_6_H_3_F_2_NOS_2_: C 34.93/34.78, H 1.51/1.46, F 18.32/18.34, N 6.79/6.76, S 31.03/30.94. MS, m/z, measured/calculated for C_6_H_3_F_2_NO^32^S_2_: 206.9620/206.9619. NMR (CDCl_3_), δ, ppm: ^1^H: 7.20 (td, 1H, *J*_1_ = 8.5, *J*_2_ = 3.7 Hz), 6.99 (td, 1H, *J*_1_ = 8.3, *J*_2_ = 3.2 Hz); ^19^F: 48.88 (td, 1H, *J*_1_ = 19.2, *J*_2_ = 7.7, *J*_3_ = 3.7 Hz). UV–vis (CH_2_Cl_2_), *λ*_max_, nm/log *ε*: 231/3.96, 247/3.69, 307/3.57. ATR IR, *v*, cm^–1^: 3126 s, 2615 w, 1861 w, 1792 w, 1745 w, 1641 w, 1593 w, 1462 vs, 1367 w, 1292 m, 1227 s, 1126 s, 1072 vs, 951 m, 841 s, 814 vs, 773 s, 706 vs, 604 vs, 563 s, 523 s, 482 m, 417 s.

Compound **10**, m.p. 80°C–81°C (decomp.). Found/calculated for C_6_HF_4_NOS_2_: C 29.51/29.63, H 0.51/0.41, F 31.46/31.25, N 5.76/5.76, S 25.05/26.37. MS, m/z, measured/calculated for C_6_HF_4_NO^32^S_2_: 242.9435/242.9430. NMR (CDCl_3_), δ, ppm: ^1^H: 5.63 (s, 1H); ^19^F: 31.2 (dd, 1F; *J*_1_ = 20.9, *J*_2_ = 14.6 Hz), 16.7 (ddd, 1F; *J*_1_ = 20.9, *J*_2_ = 14.6, *J*_3_ = 6.5 Hz), 16.0 (ddd, 1F; *J*_1_ = 20.9, *J*_2_ = 14.6, *J*_3_ = 6.5 Hz), 8.0 (dd, 1F; *J*_1_ = 20.9, *J*_2_ = 14.6 Hz). UV–vis (CH_2_Cl_2_), *λ*_max_, nm/log *ε*: 230/3.91, 248/3.66, 303/3.37. ATR IR, *v*, cm^–1^: 3099 m, 2602 w, 1628 w, 1579 w, 1468 vs, 1338 m, 1284 m, 1113 m, 1059 vs, 1032 vs, 889 s, 835 m, 781 m, 741 w, 714 w, 604 w, 577 m, 536 m, 482 m, 430 m.

Compound **11**, 105°C–110°C. Found/calculated for C_9_HF_8_NOS_2_: C 30.55/30.43, H 0.47/0.28, N 4.00/3.94, F 42.21/42.79, S 17.40/18.05. MS, m/z, measured/calculated for C_9_HF_8_NO^32^S_2_: 354.9370/354.9366. NMR [(D_3_C)_2_S=O], δ, ppm: ^1^H: 3.85 (s, 1H); ^19^F: 57.4 (m, 2F), 55.6 (m, 2F), 48.1 (dt, 1F; *J*_1_ = 24.2, *J*_2_ = 7.1 Hz), 44.7 (dt, 1F; *J*_1_ = 24.2, *J*_2_ = 6.8 Hz), 34.3 (m, 2F). UV–vis (CH_2_Cl_2_), *λ*_max_, nm / log *ε*: 230/3.96, 268/3.76, 321/3.56. IR (KBr), *v*, cm^−1^: 3030 s, 2667 m, 1632 m, 1475 vs, 1435 s, 1306 vs, 1252 vs, 1198 m, 1157 vs, 1076 vs, 947 vs, 883 s, 833 w, 702 w, 679 w, 656 w, 584 m, 557 m, 530 m, 469 m, 434 m.

Single crystals of **9**‐**11** suitable for XRD were obtained as white needles: for **9** and **10**, by gas‐phase diffusion of hexane or n‐decane into CH_2_Cl_2_ solutions at ambient‐temperature, followed by complete evaporation; for **11**, by mutual solvent diffusion at –20°C in a two‐layered system of hexane and saturated CH_2_Cl_2_ solution.

#### 2.2.6 Hydrolysis of 3^+^Cl^–^ and 4^+^Cl^–^

Crystalline **3** ^+^Cl^–^ is quite stable in air at ambient temperature. In wet CDCl_3_, **3** ^+^Cl^–^ hydrolyzes to **10** by ≈10% within 2 h (^1^H and ^19^F NMR). Crystalline **4** ^+^Cl^–^ decomposes very fast at ambient temperature into an unidentified black tar. In air at –20°C, **4** ^+^Cl^–^ quantitatively converts to **11** within 10 days.

#### 2.2.7 Conversion of 9‐11 in 2^+^‐4^+^ With Trifluoroacetic Acid

At ambient temperature, 0.03 mmol of **9**, **10**, or **11** were dissolved in 0.5 mL of CF_3_CO_2_H. ^19^F NMR (CF_3_CO_2_H), δ, ppm: **2** ^+^CF_3_CO_2_^–^: 87.3 (CF_3_CO_2_^–^ ↔ CF_3_CO_2_H), 56.9 (**2** ^+^) (56.7 in CDCl_3_) [21]; **3** ^+^CF_3_CO_2_^–^: 87.3 (CF_3_CO_2_^–^ ↔ CF_3_CO_2_H), 37.8 (m, 2F; **3** ^+^), 29.6 (m, 2F; **3** ^+^); **4** ^+^CF_3_CO_2_^–^: 87.3 (CF_3_CO_2_^–^ ↔ CF_3_CO_2_H), 59.7 (m, 2F; **4** ^+^), 54.9 (m, 4F; **4** ^+^), 32.8 (m, 2F; **4** ^+^). Attempts to isolate **2** ^+^CF_3_CO_2_^–^ resulted in a tentative solvate, **2** ^+^CF_3_CO_2_^–^ × CF_3_CO_2_H, possibly formulated as H^+^[**2**(CF_3_CO_2_)_2_]^–^, where the complex anion features double chalcogen bonding driven by two *σ*‐holes at the S atom [91]. No attempts were made to isolate salts **3** ^+^CF_3_CO_2_^–^ and **4** ^+^CF_3_CO_2_^–^ due to their higher hydrolytic instability compared to **2** ^+^.

#### 2.2.8 Complexation of 3^·^ With Naphthalene

At ambient temperature and under argon, 0.0200 g (≈0.09 mmol) of **3** ^**·**^ and 0.0113 g (0.09 mmol) of naphthalene were dissolved in 1 mL of Et_2_O. The solution was evaporated, and the residue sublimed at 45°C under 0.3 Torr to give complex **12** (0.0267 g, 85%) as small red crystals; m.p. 81°C (decomp.). Single crystals suitable for XRD were obtained as red prisms by slow sublimation in a sealed ampoule at 40°C under 0.5 Torr.

### 2.3 Crystallography

Single‐crystal XRD (see Section S2) measurements were conducted using a Bruker Kappa Apex II CCD diffractometer with MoK*α* radiation and graphite monochromator. Structures were solved using SHELXT and refined with SHELXL [92], applying absorption corrections via SADABS [93]. Hydrogen atoms were placed using a riding model, except for the NH groups in **10**‐**12**, which were located from difference Fourier maps. Crystal structures were then analyzed for shortened intermolecular contacts/SBIs using Mercury [94]. Crystallographic data were deposited at the CCDC with the deposition numbers 2452853 (for **3** ^**·**^), 2452854 (for **4** ^**·**^), 2452855 (for **6**), 2452857 (for **9**), 2452858 (for **10**), 2452859 (for **11**), and 2452856 (for **12**) and are freely accessible. Variable‐temperature powder XRD was performed with a STOE IPDS‐II system, using MoKα radiation and Oxford Cryostream for temperature control. The X‐AREA software [95] was used for data acquisition and integration.

### 2.4 Electrochemistry

CV of **3** ^**+**^Cl^–^ and **4** ^**+**^Cl^–^ was performed in CH_3_CN with 0.1 M Et_4_N^+^ClO_4_^–^ at 295 K under argon, using a three‐electrode setup and a PG 310 USB potentiostat. A stationary Pt disk (area 0.0122 cm^2^) served as the working electrode, with a Pt helix as the auxiliary and a saturated calomel electrode (SCE) as the reference electrode. The cell was connected by a salt bridge filled with the same electrolyte solution. CVs were recorded using a triangular potential sweep, and peak potentials were referenced to SCE. EPR spectra of **3** ^**·**^ and **4** ^**·**^ under electrochemical conditions were obtained by potentiostatic electrochemical reduction of **3** ^**+**^Cl^–^ and **4** ^**+**^Cl^–^at 295 K under anaerobic conditions in dry MeCN/0.1 M Et_4_N^+^ClO_4_^–^ with dioxygen removed by the freeze‐pump‐thraw method, using an Ellins P‐20X potentiostat (see Section S8).

### 2.5 Electron Paramagnetic Resonance

Solution EPR spectra were recorded using an ELEXSYS E‐540 spectrometer (X‐band, MW frequency ≈9.48 GHz, MW power 10 mW, modulation frequency 100 kHz, and modulation amplitude 0.1 mT). Electrochemically generated **3** ^**·**^ and **4** ^**·**^ were studied in potentiostatic mode under anaerobic conditions using a Pt electrode in dry MeCN with Et_4_NClO_4_. Spectra simulations were performed with *Winsim 2002* [96] using the Simplex algorithm.

Variable‐temperature powder EPR spectra of **3** ^**·**^ were measured on an ELEXSYS E‐540 spectrometer using a high‐Q cylindrical resonator Bruker ER4119HS and an ER 4131VT temperature control unit. Spin content was quantified using a rectangular double resonator and a deuterated 3‐(piperid‐1‐yl)−2,2,5,5‐tetramethyl‐pyrroline‐1‐oxyl radical as a standard. At 295 K, the spin count for **3** ^**·**^ was 165·10^15^. For **4** ^**·**^, the spectra were obtained using a Linev Systems Adani Spinscan X spectrometer (MW frequency ≈9.37 GHz, MW power 0.32 mW, modulation frequency 100 kHz, and modulation amplitude 0.1 mT) equipped with Bruker BVT3000 variable temperature unit, with Mn^2+^ in MgO as a reference. The second Mn^2+^ EPR line was used to normalize and calculate spin concentration, corresponding to 469·10^12^ S = ½ radicals. Measurements were performed in sealed glass tubes under an inert atmosphere (see Section S9).

### 2.6 Magnetometry

Magnetic susceptibility of polycrystalline **3** ^**·**^ and **4** ^**·**^ was measured over the range 2–300 K using a SQUID magnetometer under a 5 kOe field (see Section S10). Diamagnetic corrections were applied using Pascal's constants, with estimated susceptibilities of –80·10^–6^ cm^3^ mol^–1^ for **3** ^**·**^ and –108·10^–6^ cm^3^ mol^–1^ for **4** ^**·**^. The temperature dependence of susceptibility was modeled using a Curie‐like equation. Effective magnetic moments were calculated and analyzed to estimate the fraction of paramagnetic states ($\omega_{\mathrm{HS}}$) to be ≈0.17 for **3** ^**·**^ and ≈0.22 for **4** ^**·**^, which translates into a 17% for **3** ^**·**^ and 22% for **4** ^**·**^ of paramagnetic defects. Theoretical *µ*_eff_ values for **R** ^**·**^ species were taken as 1.73 *µ*_B_.

### 2.7 Theoretical Calculations

All calculations were performed using ORCA5.0.4 [97] and Gaussian09 [98] programs with implemented basis sets. IR and UV–vis spectra were calculated for fully optimized geometries by DFT and TD‐DFT [99] at the BP86 and TD‐(U)B3LYP levels of theory, respectively, with def2‐qzvppd basis set. In the IR calculations, scale factors [100] were applied; in UV–vis calculations, solvents were included using the conductor‐like polarizable continuum model (CPCM) [101] (see Sections S5 and S6). The first adiabatic ionization energies (*a*I*E*_1_) of **1** ^**·**^‐**4** ^**·**^ were calculated at the DLPNO‐CCSD(T)/aug‐cc‐pvtz level of theory [102] as the energy difference between fully optimized geometries by PBE0/aug‐cc‐pvtz and (U)PBE0/aug‐cc‐pvtz for the **R** ^+^ and **R** ^**·**^ states, respectively (**R** = **1**‐**4**; see Section S8). The hfc constants of **3** ^**·**^ and **4** ^**·**^ were calculated at the (U)PBE0 level of theory with DKH‐def2‐qzvpp and saug‐ANO‐pvtz basis sets, respectively, for fully optimized geometries (see Sections S8 and S9).

Solid‐state optimizations and interaction energy calculations were performed using the QuantumEspresso (QE) code [103] with the (U)PBE functional, Grimme‐D3 general dispersion correction [104, 105], and ultrasoft pseudopotentials [106] (see Section S11). Relaxation of **2** ^**·**^‐LT, **2** ^**·**^‐HT, and **3** ^**·**^ was carried out with fixed‐cell (FC) optimizations using the XRD cell parameters and variable‐cell (VC) optimizations. Hirshfeld surface analyses were conducted using the Crystal Explorer 21 program [81, 82] (see Section S12). Magnetic response was simulated using a first‐principles bottom‐up working strategy [107, 108, 109], in which significant magnetic exchange interactions *J*_AB_ define the magnetic topology of **2** ^**·**^‐LT, **2** ^**·**^‐HT, and **3** ^**·**^/**4** ^**·**^. (U)B3LYP magnetic *J*_AB_'s couplings obtained with broken‐symmetry [110, 111, 112, 113] (BS) approach were validated against higher‐level CASSCF/QD‐NEVPT2 calculations (see Section S13), using Gaussian09 [98] and ORCA5.0.4 [97], respectively.

## 3 Results and Discussion

Syntheses, XRD crystal and molecular structures, and magnetic properties of **1** ^**·**^ [45, 46, 53, 86] and **2** ^**·**^ [21] have been reported previously, whereas **3** ^**·**^ was only detected by EPR in solution after chemical reduction of **3** ^+^ [88].

### 3.1 Syntheses, Thermal Stability, and Heteroatom Reactivity

**SCHEME 2:**
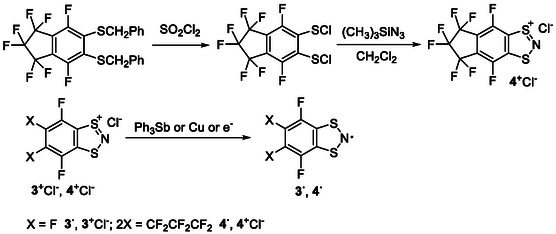
Synthesis of **4** ^+^Cl^–^ and reduction of **R** ^**+**^s in **R** ^**·**^s (**R** = **3**, **4**).

**FIGURE 2:**
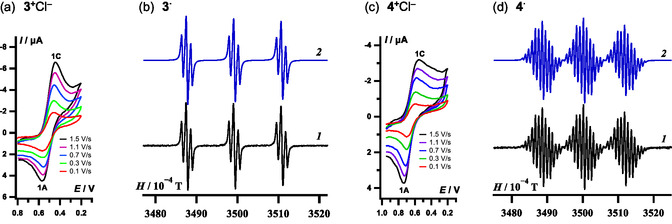
CVs of (a) **3** ^+^Cl^–^ and (c) **4** ^+^Cl^–^, together with EPR spectra of (b) **3** ^**·**^ and (d) **4** ^**·**^ (*1* – experimental, *2* – simulation) in MeCN. In CVs, 1C peak potential (V, vs. SCE): **3** ^+^, 0.46; **4** ^+^, 0.60 (cf. **1** ^**+**^, 0.15 [114, 115]; **2** ^+^, 0.40 [21]). In EPR spectra, experimental/DFT‐calculated with CPCM‐accounted solvent hfc constants *a* (G): **3** ^**·**^ [(U)PBE0/DKH‐def2‐qzvpp]: 11.60/10.70 (N^2^), 1.11/1.09 (F^5,6^), ≈0.03/0.08 (F^4,7^); **4** ^**·**^ [(U)PBE0/saug‐ANO‐pvtz]: 11.66/10.78 (N^2^), 1.89/1.87 (F^5,5′, 7,7′^), 0.72/0.98 (F^6,6′^), 0.91/1.10 (F^4,8^). Note atom numbering is extracted from Scheme 1.

In this work, radicals **3** ^**·**^ and **4** ^**·**^ were generated in solutions by both chemical (with Ph_3_Sb in CH_2_Cl_2_) and electrochemical (CV in MeCN) reduction of cations **3** ^+^ and **4** ^+^ (Scheme 2). Their formation in solution was confirmed by EPR combined with DFT (Figure 2, see Section S9) and UV–vis spectra combined with TD‐DFT (see Section S6). It should be noted that the EPR spectra of **3** ^**·**^ and **4** ^**·**^ were unaffected by the choice of solvent (CH_2_Cl_2_ or MeCN). In the solid state, the authenticity of **3** ^**·**^ and **4** ^**·**^ was demonstrated by single‐crystal XRD and powder EPR.

**SCHEME 3:**
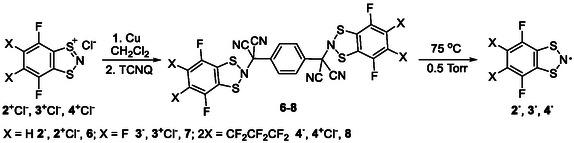
Synthesis of **6**–**8** from **R** ^+^ via intermediate **R** ^**·**^, and their conversion by mild vacuum thermolysis in isolated **R** ^**·**^ (**R** = **2**‐**4**).

**FIGURE 3:**
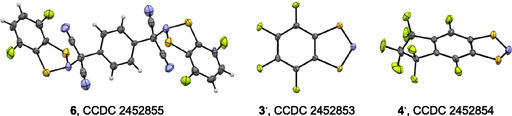
XRD molecular structure (displacement ellipsoids at 30%) of **6**, **3** ^**·**^, and **4** ^**·**^ (disordered, only the major component shown). Color code: C, gray; H, light gray; F, green; N, blue; S, yellow. For **6**, CC distances in the central benzene ring are equal due to aromatization and CS and NS distances have typical values for single bonds in N‐sulfonyl‐1,3,2‐dithiazoles; other bond distances are typical besides the CN single bond elongated to 1.523(4) Å; typical value is 1.469 Å [43, 122, 123]. For **3** ^**·**^/**4** ^**·**^, selected heterocyclic bond distances (Å) and angles (°) (see Scheme 1 for atom numbering): C7*a*–S1 1.741(2)/1.741(3), S1–N2 1.640(2)/1.650(3), N2–S3 1.652(2)/1.637(3), S3–C3*a* 1.745(2)/1.742(3), C3*a*–C7*a* 1.391(3)/1.397(4); C3*a*–C7*a*–S1 113.3(1)/113.9(2), C3*a*–S1–N2 98.72(9)/98.1(1), S1–N2–S3 115.4(1)/115.4(2), N2–S3–C3*a* 98.05(9)/98.8(1), S3–C3*a*–C7*a* 113.8(1)/112.8(2). Bicyclic 6–5 *π*‐moieties are planar within 0.111/0.201 Å for **3** ^**·**^/**4** ^**·**^. Comparison of **1** ^**·**^ [54], **2** ^**·**^ [21], **3** ^**·**^, and **4** ^**·**^ reveals that structural impact of fluorination on heterocycle is negligible.

Radicals **3** ^**·**^ and **4** ^**·**^ were long‐lived at room temperature in solution under an inert atmosphere at concentrations up to ≈0.01 M (EPR). However, complete evaporation of the solutions resulted in tarry products in which only benzo‐fused tetrathiocines [89, 90] (products of self‐condensation of **R** ^**·**^s) [22] were identified. Unexpectedly, **2** ^**·**^–**4** ^**·**^ were isolated via complexation with the strong *π*‐electron acceptor TCNQ [116, 117]. While TCNQ forms a 1:1 charge–transfer complex with **1** ^**·**^ [53, 86], with **2** ^**·**^–**4** ^**·**^ in CH_2_Cl_2_, it gives 2:1 covalent *σ*‐adducts **6**–**8** (Scheme 3, Figure 3) via 1,6‐addition; and **2** ^**+**^Cl^–^‐**4** ^**+**^Cl^–^ interact with [K]^+^[TCNQ]^**·**–^ in the same way (in other scenarios [118], [TCNQ]^**·**–^ can recombine into the *σ*‐dimer, [TCNQ]_2_^2–^). Yet, this is not a general strategy, as **2** ^**·**^–**4** ^**·**^ do not interact with the strong *π*‐electron acceptor 1,2,4,5‐tetracyanobenzene [116]. Mild vacuum thermolysis of **6**–**8** leads to their dissociation into components, as evidenced by high‐yield sublimation of crystalline **2** ^**·**^–**4** ^**·**^ (Scheme 3; Figure 3). Under ambient‐pressure thermolysis, **3** ^**·**^ and **4** ^**·**^ decompose, and only **2** ^**·**^ sublimes (see Section S7).

The difference in reactivity between **1** ^**·**^ and **2** ^**·**^–**4** ^**·**^ toward TCNQ can be attributed to the *π*‐fluoro effect, i.e., energy stabilization of occupied *π*‐MOs of (het)arenes up to ≈1 eV upon fluorination [119]: whereas **1** ^**·**^ is a good *π*‐donor toward TCNQ [53, 85], its fluoro derivatives **2** ^**·**^–**4** ^**·**^ are not, which is consistent with DLPNO‐CCSD(T)‐calculated *a*I*E*_1_ values for **1** ^**·**^‐**4** ^**·**^ of 4.54, 4.83, 4.89, and 5.05 eV, respectively (see Section S8). The addition suggests aromatization of the carbocycle of TCNQ and, therefore, can be considered thermodynamically driven. Although 1,6‐addition reactions are characteristic of TCNQ, with closed‐shell (het)arenes they typically feature a 1:1 stoichiometry and H‐atom transfer from the (het)arene [120] resembling the 1:1 addition observed for another strong *π*‐acceptor, bis(thiadiazolo)pyrazine [121]. In contrast, the much rarer 1,6‐addition reactions of *σ*‐radicals (e.g., Me_2_(NC)C^**·**^) with TCNQ feature 2:1 stoichiometry [120]. To the best of our knowledge, reactions between TCNQ and *π*‐radicals have not been reported previously, and the formation of **6**–**8** is unique.

**FIGURE 4:**
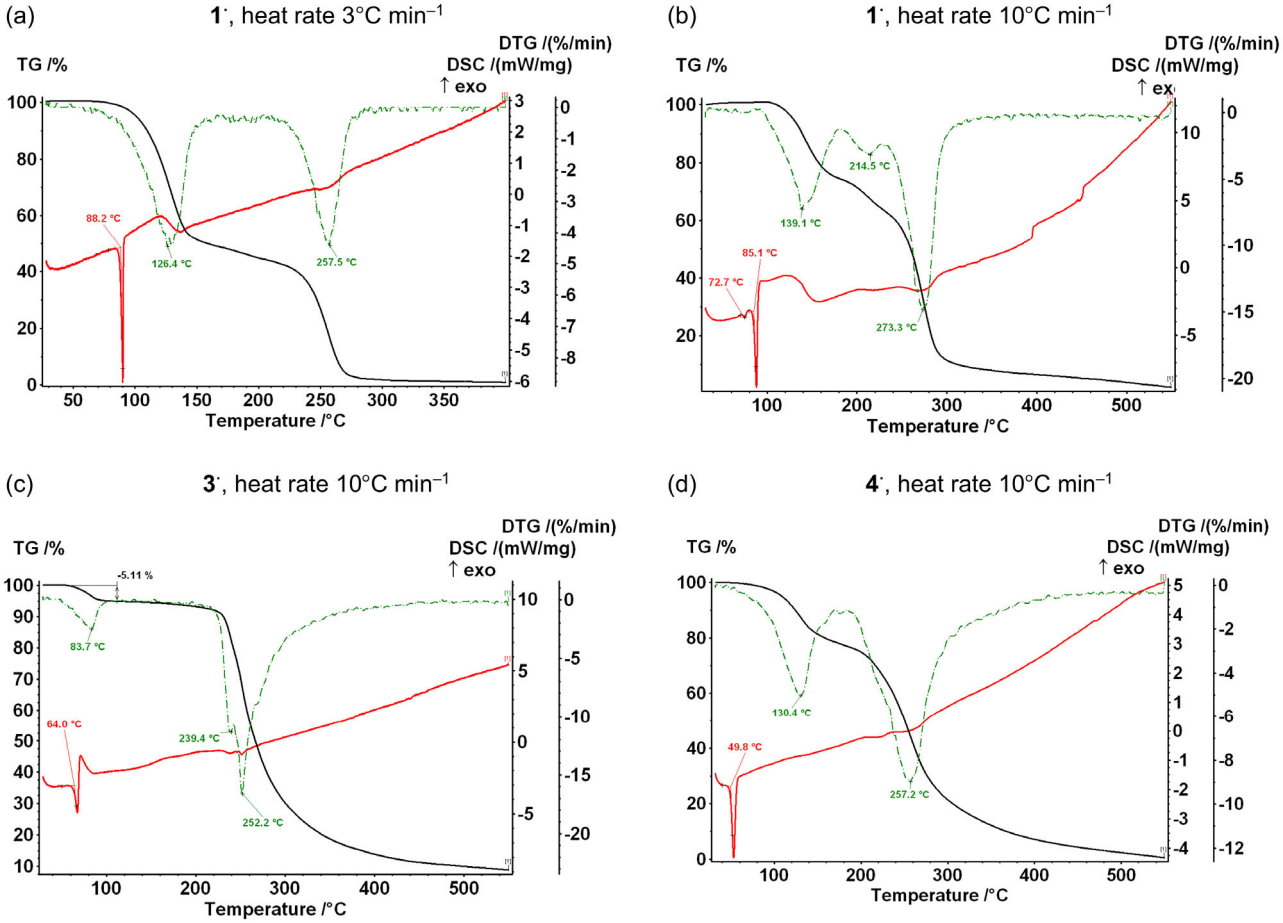
TG‐DSC of (a,b) **1** ^**·**^, (c) **3** ^**·**^, and (d) **4** ^**·**^. Note heat rate is 3°C min^–1^ in (a) and 10°C min^–1^ in (b–d). Color code: TG, black; DTG, green; DSC, red.

Ambient‐pressure thermal stability of **1** ^**·**^–**4** ^**·**^ differs (Figure 4). Compound **1** ^**·**^ melts at ≈86°C; the temperature of its decomposition accompanied by sublimation is heat‐rate dependent: ≈126°C at the rate 3°C min^–1^ and ≈139°C at the rate 10°C min^–1^ (Figure 4a,b). Compound **2** ^**·**^ melts at 114°C with decomposition [21]. Slow decomposition of **3** ^**·**^ occurs even at room temperature under an inert atmosphere. DSC data suggest fast exothermic decomposition of **3** ^**·**^ at 64°C (Figure 4c): ≈5% mass loss roughly corresponds to the nitrogen content in **3** ^**·**^ (≈6%). Most likely, the decomposition products are N_2_ and polyfluorinated dibenzotetrathiocine [89, 90] (or related oligomers/polymers); a related tetraselenocine was previously obtained as a product of the reduction of 1,3,2‐benzodiselenazolium, presumably involving an unstable di‐Se analog of **1** ^**·**^ as the key intermediate [124]. In contrast, **4** ^**·**^ melts without decomposition at 52°C (Figure 4d). At higher temperatures, its evaporation proceeds up to ≈120°C, where an inflection of the TG curve indicates decomposition. According to variable‐temperature powder XRD, crystalline **3** ^**·**^ and **4** ^**·**^ become amorphous after heating at 50°C for 1 h and 42°C for 2 h, respectively (see Section S2). Thus, among the discussed tetrad, **1** ^**·**^ is the most thermally stable, i.e., fluorination reduces the thermal stability of **R** ^**·**^s. However, the most interesting observation is the small endothermic DSC peak for **1** ^**·**^ at ≈73°C (346 K) with the heat rate of 10°C min^–1^ (Figure 4b), most likely reflecting the **1** ^**·**^‐LT → **1** ^**·**^‐HT phase transition [45, 46, 53, 67].

**SCHEME 4:**
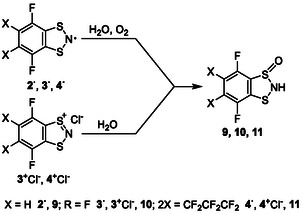
Interactions of **2** ^**·**^–**4** ^**·**^, **3** ^+^, and **4** ^+^ with atmospheric or solvent moisture; for **2** ^**·**^–**4** ^**·**^, they are seemingly assisted by O_2_.

**FIGURE 5:**
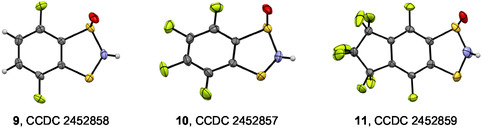
XRD molecular structures (displacement ellipsoids at 30%) of **9**–**11**. Color code: C, gray; H, light gray; F green; N, blue; O, red; S, yellow. Selected heterocyclic bond distances (Å) and angles (°) of **9**/**10**/**11** (see Scheme 1 for atom numbering): S1–O 1.470(2)/1.482(3)/1.472(4), C7*a*–S1 1.783(2)/1.781(3)/1.796(4), S1–N2 1.685(3)/1.645(4)/1.647(6), N2–S3 1.719(2)/1.700(3)/1.699(6), S3–C3*a* 1.783(2)/1.736(3)/1.728(5), C3*a*–C7*a* 1.393(3)/1.386(4)/1.393(6); C3*a*–C7*a*–S1 114.2(2)/114.7(2)/115.5(3), C7*a*–S1–N2 93.1(1)/88.9(1)/90.4(2), S1–N2–S3 115.6(1)/119.6(2)/121.8(3), N2–S3–C3*a* 93.9(1)/90.0(2)/91.9(3), S3–C3*a*–C7*a* 115.5(2)/115.7(2)/115.9(4).

**FIGURE 6:**
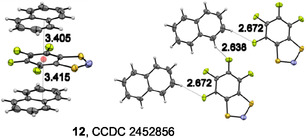
Fragments of XRD crystal structure of **12** (displacement ellipsoids at 30%) exhibiting shortened contacts between **3** ^**·**^ and naphthalene along one *π*‐stack, together with H···F lateral contacts between naphthalenes and **3** ^**·**^ radicals belonging to four different *π*‐stacks. Color code: C, gray; H, light gray; F, green; N, blue; O, red; S yellow. The sum of VdW radii of C and S atoms is ≈3.66 Å, of two C atoms ≈3.54 Å; and of F and H atoms, ≈2.66 Å [129]. In the homocrystal, **3** ^**·**^ is planar within 0.111 Å and, in complex **12**, within 0.087 Å.

Regarding the reactivity of **R** ^**·**^s, only a few nonsystematic examples have been reported to date [21, 22]. Besides the previously discussed addition to TCNQ (Scheme 3), it is found that **2** ^**·**^–**4** ^**·**^ slowly transform into 2H‐1‐oxo‐1,3,2‐dithiazoles **9**–**11**, respectively, under the influence of atmospheric H_2_O and O_2_ (Scheme 4; Figure 5). Compounds **10** and **11** are also identified as products of hydrolysis of **3** ^+^Cl^–^ and **4** ^+^Cl^–^ by atmospheric moisture (Scheme 4), whereas **1** ^+^Cl^–^ was previously reported as stable in aqueous solution [86]. Nonfluorinated 2‐X analogs of **9**–**11** are chiral but easily epimerize under ambient conditions [125], and the same is expected for **9**–**11**. Protic acids, e.g., CF_3_CO_2_H, convert **9**–**11** back to **2** ^+^–**4** ^+^. Finally, it has been shown that radical **3** ^**·**^ forms a 1:1 *π*‐complex with naphthalene (**12**, Figure 6), whereas **1** ^**·**^ and octafluoronaphthalene do not interact [67]. Similar to **2** ^**·**^, the XRD crystal structure of **12** exhibits *π*‐stacks with shortened intermolecular contacts, together with lateral H···F contacts —one slightly above and another slightly below the sum of the VdW radii— most likely representing weak hydrogen bonds. Preliminary SQUID magnetometry of **12** displays hysteretic magnetic bistability in the 140–190 K range. Although **3** ^**·**^ in its individual form decomposes slowly at room temperature and rapidly at 64°C (Figure 4c), **12** is stable up to ≈80°C (TD‐DSC), i.e., to the melting point of naphthalene. Complexation with high‐melting aromatics may thus become a method for stabilizing thermally sensitive **R** ^**·**^s and warrants further exploration. Based on complexation with Lewis *π*‐acids/*π*‐bases, the discussed **R** ^**·**^s, similar to many other chalcogen‐nitrogen *π*‐heterocycles [126, 127, 128], can be classified as Lewis ambiphiles, i.e., compounds simultaneously exhibiting both Lewis acidic and Lewis basic properties.

### 3.2 Magnetic Properties

For potential applications, the most interesting properties are the magnetics response of **R** ^**·**^s around room temperature (≈298 K) [34, 50, 57, 64]. For **1** ^**·**^, the **1** ^**·**^‐HT ↔ **1** ^**·**^‐LT hysteretic loop of ≈20 K (T↑ 346 K, T↓ 324 K) was observed upon supercooling of **1** ^**·**^‐HT; further supercooling led to an AFM ordering at 11 K [45, 46, 53]. Radical **2** ^**·**^ exhibits the **2** ^**·**^‐HT ↔ **2** ^**·**^‐LT hysteretic loop of ≈10 K closer to room temperature (T↑ 322 K, T↓ 313 K) [21]. In this work, magnetic properties of **3** ^**·**^ and **4** ^**·**^ are studied using variable‐temperature powder EPR from low temperatures up to 330 K, and SQUID magnetometry up to 300 K (see Sections S9 and S10, respectively).

**FIGURE 7:**
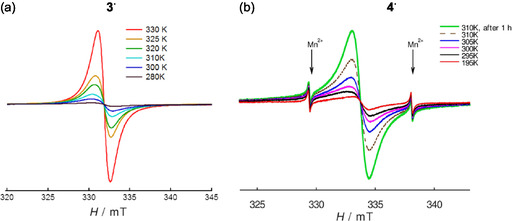
Color‐indicated variable‐temperature powder EPR spectra of (a) **3** ^**·**^ obtained with a stable nitroxide‐radical standard; and of (b) **4** ^**·**^, where arrows indicate the EPR lines of the Mn^2+^ standard.

Variable‐temperature powder EPR shows that **3** ^**·**^ is nearly silent below 240 K, with only a weak anisotropic signal likely due to paramagnetic defects. Between 240 and 330 K, its EPR intensity increases sharply, indicating a temperature‐dependent shift between diamagnetic and paramagnetic responses (Figure 7a). Above 315 K, **3** ^**·**^ becomes unstable, decomposing at 330 K, with irreversible amorphization confirmed by powder XRD (see Section S2). Similarly, **4** ^**·**^ is EPR‐silent below 275 K. Yet, it becomes active at higher temperatures, with a rapid signal increase between 300 and 330 K (Figure 7b). At 310 K, spin concentration rises, but at 320 K, **4** ^**·**^ decomposes, with EPR intensity slowly decreasing and powder XRD confirming amorphization (see Section S2). The thermal behavior of **3** ^**·**^ and **4** ^**·**^ in the range ≈270–310 K can be described by van’t Hoff isobars, suggesting endothermic processes with Δ*H* values of 31.8 and 56.8 kJ mol^–1^, respectively. For **4** ^**·**^, Δ*H* is nearly identical to that of **2** ^**·**^ [21] (56.4 kJ mol^–1^) [130].

**FIGURE 8:**
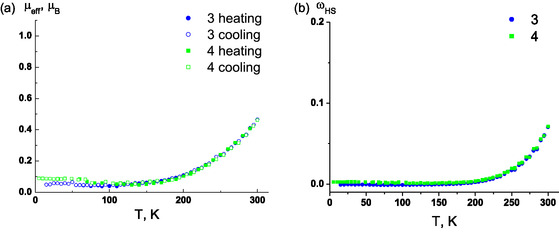
Temperature dependencies of (a) *μ*_eff_ and (b) paramagnetic defects fraction *ω*_HS_ of **3** ^**·**^ and **4** ^**·**^ in the 2–300 K range.

According to SQUID magnetometry (Figure 8a), **3** ^**·**^ and **4** ^**·**^ are diamagnetic in ≈2–200 K range. The residual values of the *µ*_eff_ effective magnetic moment below 200 K are attributed to paramagnetic defects *ω*_HS_, whose fraction are ≈0.17 for **3** ^**·**^ and ≈0.22 for **4** ^**·**^ (Figure 8b). Up to 300 K, *µ*_eff_ increases almost identically for both **3** ^**·**^ and **4** ^**·**^; its changes in the temperature range 200–300 K are reversible, and the heating and cooling *µ*_eff_ (T) curves practically coincide. Thus, **3** ^**·**^ and **4** ^**·**^ are stable up to 300 K, and changes in the *µ*_eff_(*T*) curves are indicative of temperature‐dependent shift between diamagnetic and paramagnetic responses. Similar to **2** ^**·**^ [21], at 300 K, *µ*_eff_ reaches values corresponding to 7% of the paramagnetic state of **3** ^**·**^and **4** ^**·**^ in the samples, i.e., not all radicals behave as ideal S = 1/2 paramagnetic centers. Thus, together with thermal stability, fluorination affects the lower‐temperature threshold of paramagnetism of **1** ^**·**^–**4** ^**·**^ shifting it toward room temperature: **1** ^**·**^ (324 K) → **2** ^**·**^ (313 K) → **3** ^**·**^ (≈315 K) → **4** ^**·**^ (≈310 K). However, for **3** ^**·**^ and **4** ^**·**^ fully completed magneto‐structural transition from diamagnetism to paramagnetism is not observed since it is very close to the thresholds of their thermal instabilities.

### 3.3 Crystal Structure Analysis

The crystal structures of **3** ^**·**^ and **4** ^**·**^ are similar to each other and different from those of **1** ^**·**^ and **2** ^**·**^, and the magnetic behavior of **3** ^**·**^ and **4** ^**·**^ (diamagnetism) differs fundamentally from that of **1** ^**·**^ [45, 46, 53–56] and **2** ^**·**^ [21] (magnetic bistability), which exhibit the coexistence of low‐temperature (LT) and high‐temperature (HT) crystallographic phases. To understand the magnetic response to fluorination, the analysis of **1** ^**·**^‐**4** ^**·**^ from radical to crystal is thus required.

**FIGURE 9:**
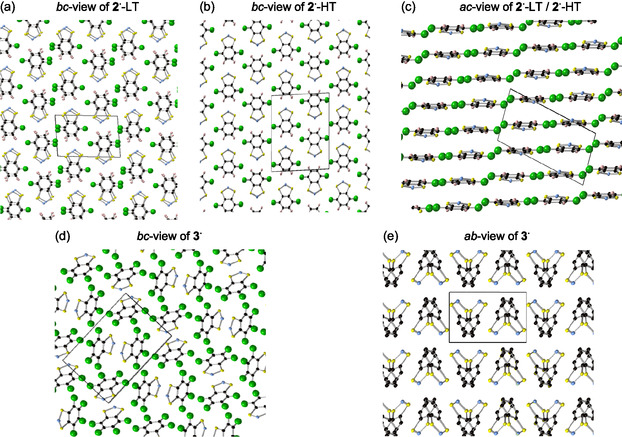
Lamellar array of nearly eclipsed **2** ^**·**^ radicals displaying two distinct crystallographic phases: (a) *bc*‐view of **2** ^**·**^‐LT; (b) *bc*‐view of **2** ^**·**^‐HT; and (c) *ac*‐view of **2** ^**·**^‐LT/**2** ^**·**^‐HT. Herringbone array of **3** ^**·**^ radicals (F atoms are omitted for clarity): (d) *bc*‐view and (e) *ab*‐view.

**FIGURE 10:**
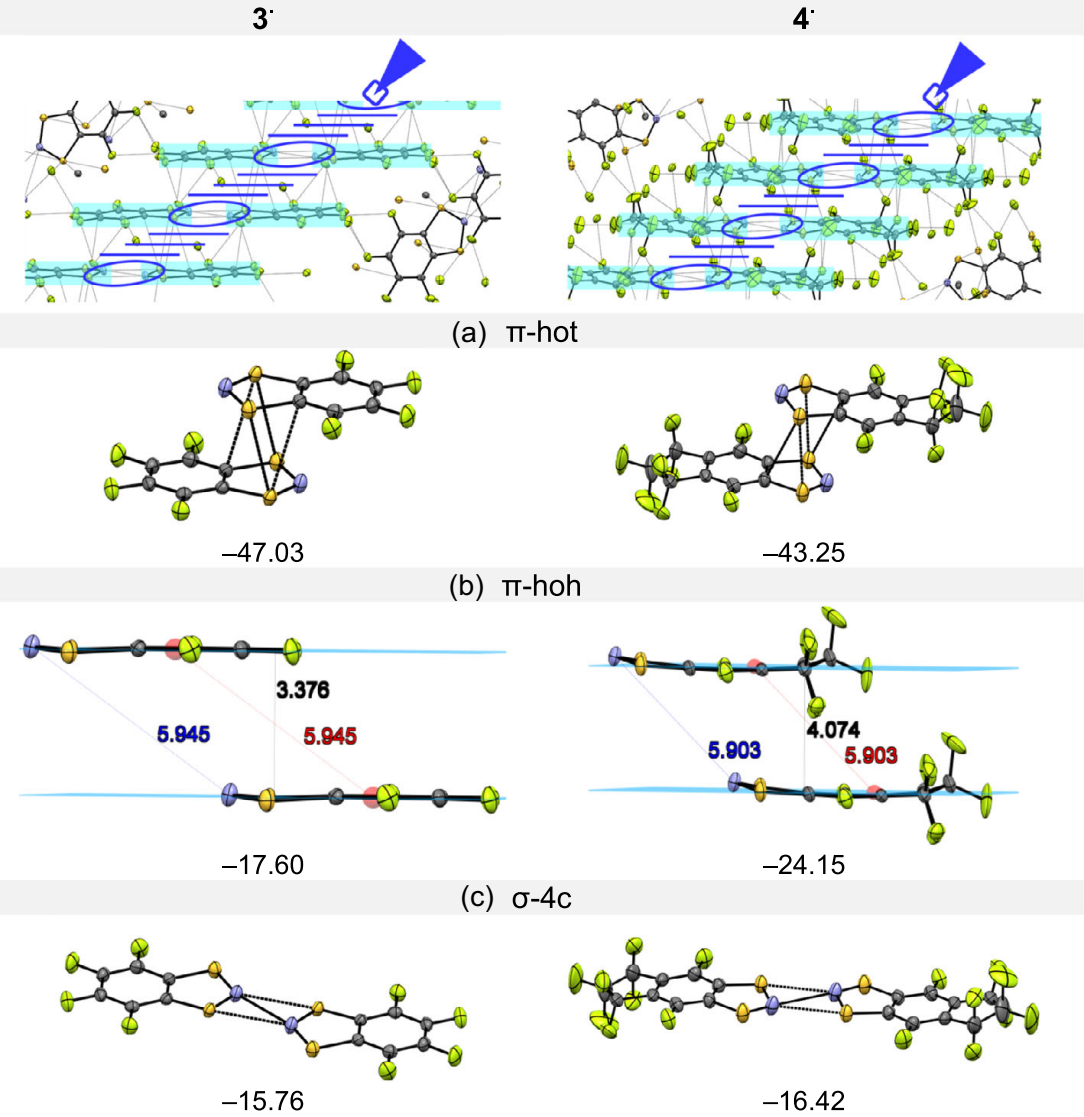
The *σ*‐4c‐mediated zip‐*π*‐stack synthon of crystalline **3** ^**·**^ and **4** ^**·**^ (coplanar *σ*‐4c‐interaction is highlighted in deep blue and *π*‐stack interaction in light blue), together with the building blocks and their interaction energies (kJ mol^–1^): (a) *π*‐stack head‐over‐tail pair (*π*‐hot), (b) *π*‐stack offset head‐over‐head pair (*π*‐hoh), and (c) coplanar 4 center‐head‐to‐tail pair (*σ*‐4c).

The supramolecular synthon of the **2** ^**·**^‐LT phase consists of a *π*‐stack of **R** ^**·**^ pairs with alternating interplanar distances, whereas that of the **2** ^**·**^‐HT phase also consists of *π*‐stacks of **R** ^**·**^ pairs but with a uniform interplanar distance. Both synthons display a lamellar array of nearly eclipsed head‐over‐head **2** ^**·**^s (Figure 9a,b; note that here the term cis‐cofacial could also be used to describe the radical arrangement). Compound **3** ^**·**^ (Figure 9d,e), structurally characterized at two distinct temperatures, 296 and 200 K (see Section S2), reveals no first‐order phase transition. Quantitative Hirshfeld surface analysis corroborates that **2** ^**·**^‐HT and **2** ^**·**^‐LT exhibit two distinct interaction patterns, whereas **3** ^**·**^ at 296 and 200 K shows no significant packing alterations in response to temperature variation, indicating the presence of a single polymorph (see Section S12). Hereafter, discussion of **3** ^**·**^ refers only to its structure at 200 K. Note that all arguments and conclusions drawn for **3** ^**·**^ are applicable to **4** ^**·**^ since the XRD crystal structures of **3** ^**·**^ and **4** ^**·**^ are similar (Figure 10; Section S11).

Both **3** ^**·**^ and **4** ^**·**^ feature lateral dimers with shortened S···N contacts of ≈3.04 and 3.07 Å, respectively (sum of VdW radii, ≈3.55 Å) [129], and shortened N···N contacts of ≈3.07 and 2.95 Å, respectively (sum of VdW radii, ≈3.32 Å) [129]. Such coplanar arrangement of contacts is referred to as a four‐center (*σ*‐4c) pair (Figure 10c) [128, 131]. The dimers are *π*‐stacked with offset by means of shortened S···S contacts of ≈3.33 and 3.27 Å, respectively (sum of VdW radii, ≈3.78 Å [129]. Note that here the terms *trans‐cofacial* and *slipped‐cis‐cofacial* could be used to describe the radical arrangement of head‐over‐tail (*π*‐hot) and head‐over‐head (*π*‐hoh) pairs in Figure 10a,b, respectively. These contacts are longer than those in the *π*‐dimer of **1** ^**·**^ (≈3.18 Å) [54], and such arrangement of **R** ^**·**^s leads to a very unique synthon: a *σ*‐4c‐mediated zip‐*π*‐stack (Figure 10). Previously, similar either lateral or stacked dimers were observed for **R** ^**·**^s but never combined in one single crystal lattice [23, 47, 51, 54, 132, 133].

To assess the stability of the discussed synthons and, in turn, of the resulting crystal packing of **2** ^**·**^ and **3** ^**·**^, identification of **R** ^**·**^ pairs belonging to different synthons and involved in different SBIs is needed. For **3** ^**·**^, the pairs with largest interaction energy are those involved in the *σ*‐4c‐mediated zip‐*π*‐stack synthon, aggregating *π*‐hot, *π*‐hoh, and *σ*‐4c pairs (Figure 10a–c; see Section S11). Each **3** ^**·**^ establishes one *π*‐hot, two *π*‐hoh and one *σ*‐4c contacts, resulting in an interaction energy per radical (*E*_int_/**R** ^**·**^) of –48.99 kJ mol^–1^ in an isolated synthon. The *E*_int_/**R** ^**·**^ becomes –84.84 kJ mol^–1^ when accounting for all SBIs, i.e., for the inter‐synthon neighboring **R** ^**·**^s, which amount for ten pairs. This means that *≈*60% of the interaction energy is due to the synthon *per se* and *≈*40% to lateral contacts between synthons. To corroborate the analysis performed using isolated **R** ^**·**^ pairs of radicals, 1) the crystal and 2) the synthon of **3** ^**·**^ were fully optimized, and their corresponding *E*_int_/**R** ^**·**^ values were found to be –76.50 and –50.41 kJ mol^–1^, respectively (66% synthon vs. 36% lateral contacts), which agree well with the previous data. It thus follows that apparently cooperative effects in the crystal lattice are not much significant for **3** ^**·**^.

**FIGURE 11:**
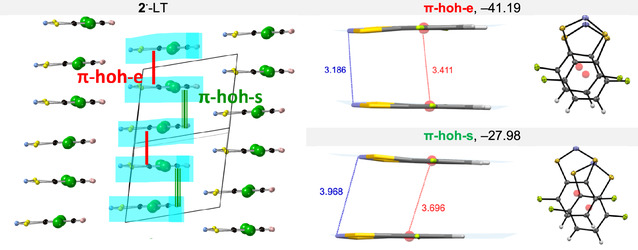
The alternant 1D *π*‐stack synthon of **2** ^**·**^‐LT (*π*‐interactions are highlighted in light blue), together with building blocks and their interaction energies (kJ mol^–1^): *π*‐stacked head‐over‐head eclipsed pair (*π*‐hoh‐e, in red), and *π*‐stacked head‐over‐head slipped pair (*π*‐hoh‐s, in green).

For **2** ^**·**^‐LT (Figure 11), the alternant 1D *π*‐stack synthon aggregates relatively well‐eclipsed *π*‐hoh **R** ^**·**^ pairs (see Section S11 for full discussion), where *π*‐hoh‐e is practically eclipsed and *π*‐hoh‐s displays certain degree of latitudinal slippage. The contacts between synthons are also found to be ten distinct pair interactions, including coplanar and interplanar pairs with varying degrees of latitudinal and longitudinal slippage. According to the synthon, any given **2** ^**·**^ in **2** ^**·**^‐LT establishes only one *π*‐hoh‐e and one *π*‐hoh‐s pairs. Therefore, due to the number of contacts, *E*_int_/**R** ^**·**^ in an isolated synthon is expected to be much smaller than when all SBIs are considered: –36.28 versus –85.65 kJ mol^–1^. These values are consistent with those obtained after optimization of synthon and crystal (–36.83 vs. –83.68 kJ mol^–1^, respectively). In this case, the contribution of lateral contacts to the interaction energy overcomes that of the synthon *per se* (42% synthon vs. 58% lateral contacts). The same trend is observed for **2** ^**·**^‐HT (see Section S11), as it also possesses a similar synthon.

Comparison of *E*_int_/**R** ^**·**^ reveals that the *σ*‐4c‐mediated zip‐*π*‐stack synthon (*σ*4c‐zip*π*) of **3** ^**·**^ is more stable per **R** ^**·**^ than the 1D *π*‐stack synthon (1D‐*ππ*) of **2** ^**·**^‐LT/HT (ca. –50.2 vs. –37.6 kJ mol^–1^, respectively). However, once all contacts are considered, the *E*_int_/**R** ^**·**^ is ≈–83.6 kJ mol^–1^ in both cases. Toward understanding why **2** ^**·**^ and **3** ^**·**^ have clearly different crystal packing, in silico modeling was employed: 1) the **3** ^**·**^ synthon was combined with **2** ^**·**^ as a radical ($E_{\sigma 4c-\mathrm{zip}\pi }^{2^{\cdot }}$) and the **2** ^**·**^‐LT synthon with **3** ^**·**^ as a radical ($E_{1D-\pi \pi }^{3^{\cdot }}$); and 2) the radicals of the entire crystal of **3** ^**·**^ were replaced by **2** ^**·**^ ($E_{3^{\cdot }}^{2^{\cdot }}$), and those of **2** ^**·**^ by radical **3** ^**·**^ ($E_{2^{\cdot }}^{3^{\cdot }}$). It was found that **3** ^**·**^ is more stable arranging in a *σ*4c‐zip*π* synthon by 4.2 kJ mol^–1^, while **2** ^**·**^ can adapt to both synthons since the interaction energy difference of 0.8 kJ mol^–1^ is almost negligible (Table 1). This finding might indicate that H···F hydrogen bonds along the *σ*4c‐zip*π* synthon may be as significant as those along the 1D‐*ππ* synthon. Regarding the solid‐state results, **3** ^**·**^ is 10.5 kJ mol^–1^ more stable using its own radical arrangement, which means that the contribution of the lateral SBIs is 6.3 kJ mol^–1^ since the zip‐*π*‐stack array accounts for the remaining 4.2 kJ mol^–1^. For **2** ^**·**^ in the solid‐state, now it becomes evident that lateral contacts also stabilize its own radical arrangement by ≈2.1 kJ mol^–1^ per **R** ^**·**^.

**TABLE 1:** Energy difference Δ(*E*_int_/**R** ^·^) (kJ mol^–1^) between the $E_{\sigma 4c-\mathrm{zip}\pi }^{3^{\cdot }}$ synthon of **3** ^·^ and in silico synthon of **3** ^·^ using the $E_{1D-\pi \pi }^{3^{\cdot }}$ synthon of **2** ^·^, between the $E_{1D-\pi \pi }^{2^{\cdot }}$ synthon of **2** ^·^ and in silico synthon of **2** ^·^ using the $E_{\sigma 4c-\mathrm{zip}\pi }^{2^{\cdot }}$ synthon of **3** ^·^; and between the entire crystals of **3** ^·^ and **2** ^·^ using solid‐state $E_{3^{\cdot }}^{3^{\cdot }}$($E_{2^{\cdot }}^{2^{\cdot }}$) and $E_{2^{\cdot }}^{3^{\cdot }}$ ($E_{3^{\cdot }}^{2^{\cdot }}$) data.

Synthons	Δ($E_{\mathrm{int}}/R^{\cdot }$)	Crystals	Δ($E_{\mathrm{int}}/R^{\cdot }$)
$E_{\sigma 4c-\mathrm{zip}\pi }^{3^{\cdot }}-E_{1D-\pi \pi }^{3^{\cdot }}$	−4.2	$E_{3^{\cdot }}^{3^{\cdot }}-E_{2^{\cdot }}^{3^{\cdot }}$	−10.5
$E_{1D-\pi \pi }^{2^{\cdot }}-E_{\sigma 4c-\mathrm{zip}\pi }^{2^{\cdot }}$	−0.8	$E_{2^{\cdot }}^{2^{\cdot }}-E_{3^{\cdot }}^{2^{\cdot }}$	−2.5

### 3.4 Computational Study of the Magnetic Response

**FIGURE 12:**
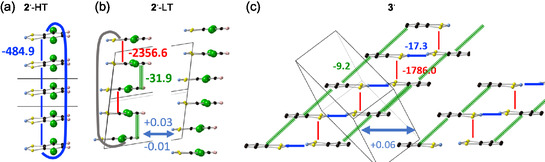
Magnetic models for (a) **2** ^**·**^‐HT, (b) **2** ^**·**^‐LT, and (c) **3** ^**·**^ featuring *J*_AB_ magnetic couplings (cm^–1^) highlighted by colors. Negative *J*_AB_'s correspond to AFM, and positive to FM, interactions. The **R** ^**·**^s interacting to generate the magnetic topology are those involved in the crystal packing synthons of the HT and LT phases of **2** ^**·**^ and **3** ^**·**^ (Table 2).

The distinct magnetic response of **2** ^**·**^ and **3** ^**·**^, i.e., bistability and diamagnetism, respectively, is evaluated following a first‐principles bottom‐up procedure [107, 108, 109]. Let us stress that the results obtained for **3** ^**·**^ can be extrapolated on **4** ^**·**^ (see Section S13). The selection of magnetically important **R** ^**·**^ pairs (in terms of **R** ^**·**^···**R** ^**·**^ distances in the crystal packing) is specified above in the context of calculating the interaction energy between a reference **R** ^**·**^ and its surrounding congeners. A systematic evaluation of *J*_AB_ magnetic interactions for all possible **R** ^**·**^ pairs, both within and between synthons, yields very few significative *J*_AB_ couplings (Table 2). Once all *J*_AB_'s are evaluated, the magnetic topology can be next defined in terms of all computed significant *J*_AB_'s to select a representative magnetic model (Figure 12) to solve the secular equation problem together with the energy spectra and corresponding spin quantum numbers.

**TABLE 2:** (U)B3LYP/def2‐tzvp‐calculated magnetic couplings *J*_AB_ (cm^–1^) for significant **R** ^·^ pair interactions.^a^

**R** ^**·**^	*J*_AB_ within synthon			*J*_AB_ between synthons	
**2** ^**·**^‐HT	*π*‐hoh‐reg				
	−484.9				
**2** ^**·**^‐LT	*π*‐hoh‐e	*π*‐hoh‐s		i‐hoh‐par‐*π* 1	co‐htt‐lat 2
	−2356.6	−31.9		+0.03	−0.01
**3** ^**·**^ at 200 K	*π*‐hot	*π*‐hoh	co‐4c‐htt	orthogonal 2	
	−1786.0	−9.2	−17.3	+0.60	
**4** ^**·**^	*π*‐hot	*π*‐hoh	co‐4c‐htt	orthogonal 2	
	−2050.3	−4.0	−33.7	−4.02	

a Negative values correspond to AFM, and positive to FM, interactions.

For **2** ^**·**^‐HT (Figure 12a, Table 2), only one non‐negligible magnetic coupling, $J_{\pi -\mathrm{hoh}-\mathrm{reg}}^{2^{\cdot }-\mathrm{HT}}=-484.9$ cm^–1^, is found, that gives rise to a magnetic topology of isolated regular AFM coupled 1D *π*‐stacks. For **2** ^**·**^‐LT (Figure 12b, Table 2), the magnetic topology consists of alternant AFM coupled 1D *π*‐stacks ($J_{\pi -\mathrm{hoh}-e}^{2^{\cdot }-\mathrm{LT}}=-2356.6$ and $J_{\pi -\mathrm{hoh}-s}^{2^{\cdot }-\mathrm{LT}}=-31.9$ cm^–1^). The stacks exhibit very weak ferromagnetic (FM) and AFM lateral interactions ($J_{i-\mathrm{hoh}-\mathrm{par}-\pi 1}^{2^{\cdot }-\mathrm{LT}}$= +0.03 and $J_{\mathrm{co}-\mathrm{htt}-\mathrm{lat}2}^{2^{\cdot }-\mathrm{LT}}$= –0.01 cm^–1^). Therefore, the *π*‐stacks can, in practice, be considered effectively to be isolated, as in **2** ^**·**^‐HT. The magnetic model for both **2** ^**·**^‐HT and **2** ^**·**^‐LT is thus formed by cyclic 1D chain models (Figure 12a,b).

For **3** ^**·**^ (Figure 12c, Table 2), the magnetic topology at 200 K involves AFM strong‐rail spin ladders with two distinct rail interactions, $J_{\pi -\mathrm{hot}}^{3^{\cdot }}$= –1786.0 and $J_{\pi -\mathrm{hoh}}^{3^{\cdot }}$= –9.2 cm^–1^, together with a rung interaction, $J_{\sigma 4c-\mathrm{htt}}^{3^{\cdot }}$= –17.3 cm^–1^, ferromagnetically interacting with nearby spin ladders via $J_{\text{orthogonal}2}^{3^{\cdot }}$= +0.06 cm^–1^. Magnetic models with and without inter‐spin‐ladder interactions are used to simulate the magnetic response, and no significant change is observed upon inclusion.

**FIGURE 13:**
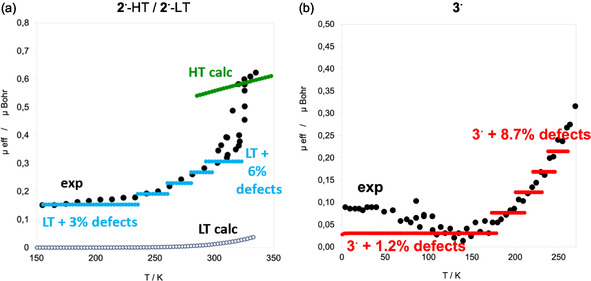
Magnetic response of (a) **2** ^**·**^ and (b) **3** ^**·**^. For **2** ^**·**^: black, experiment; green, simulation for **2** ^**·**^‐HT; blue, simulation for **2** ^**·**^‐LT with defects; empty blue, simulation for **2** ^**·**^‐LT. For **3** ^**·**^: black, experiment; red, simulation accounting for introduced paramagnetic defects in **3** ^**·**^.

For **2** ^**·**^‐LT and **2** ^**·**^‐HT, the experimental [21] magnetic data is reproduced using 16‐**R** ^**·**^‐cyclic AFM chain models (Figure 12a,b). For **2** ^**·**^‐HT, the agreement between experimental and simulated data is good; for **2** ^**·**^‐LT, however, the simulations result in magnetic silence (Figure 13a). For **2** ^**·**^ [21], it was already indicated that to reproduce the experimental data a value of $J_{\pi -\mathrm{hoh}-e}^{2^{\cdot }-\mathrm{LT}}\approx$ –500 cm^–1^ was required. The potential influence of uncoupled **2** ^**·**^ moieties is studied by simulating their presence within the cyclic AFM chain model, preventing one **2** ^**·**^ from coupling in AFM mode to adjacent **2** ^**·**^ units. Consequently, one $J_{\pi -\mathrm{hoh}-e}^{2^{\cdot }-\mathrm{LT}}$ and one $J_{\pi -\mathrm{hoh}-s}^{2^{\cdot }-\mathrm{LT}}$ magnetic couplings must be removed to create one single paramagnetic defect (see Section S13). The contribution of uncoupled **2** ^**·**^s quantitatively rationalizes the experimental magnetic response of **2** ^**·**^‐LT (Figure 13a).

For **3** ^**·**^ and **4** ^**·**^, the paramagnetic state fraction *ω*_HS_ is ≈0.17 and ≈0.22 (i.e., 17% and 22%), respectively (Figure 8b). Simulations accounting for paramagnetic defects to reproduce the experimental magnetic response: one $J_{\pi -\mathrm{hot}}^{3^{\cdot }}$, two $J_{\pi -\mathrm{hoh}}^{3^{\cdot }}$, and one $J_{\sigma -4c-\mathrm{htt}}^{3^{\cdot }}$ interactions are removed to create one paramagnetic defect (see Section S13 for **3** ^**·**^); the same applies to **4** ^**·**^) indicate that the presence of unpaired **R** ^**·**^s reproduces the experimental magnetic response (see Figure 13b). Note that the fraction of paramagnetic defects needed is smaller than 0.17 and varies depending on the temperature. This is a clear indication that thermal fluctuations play an important structural role and should be incorporated into simulations. However, this is currently out of the scope of this paper.

Overall, the magnetic response of **2** ^**·**^‐LT and **3** ^**·**^ calculated using defect‐ignoring cyclic magnetic models differs from the experimental data, whereas simulations accounting for uncoupled **R** ^**·**^s successfully reproduce the experimental magnetic response (Figure 13).

The computational analysis of **2** ^**·**^ and **3** ^**·**^ provides a sound interpretation of the structural synthons and magnetic response of the materials in terms of magnetic susceptibility. Interestingly, the structural synthons are also the **R** ^**·**^ pairs driving the magnetic couplings that define the actual magnetic topology. The 1D magnetic topology expected from direct observation of the crystal packing was corroborated for both **2** ^**·**^‐HT and **2** ^**·**^‐LT. The novel synthon consisting of *σ*‐4c‐mediated zip‐*π*‐stacked arrangement of **3** ^**·**^/**4** ^**·**^, which also governs the magnetic topology, requires careful thinking since it is not obvious in the crystal packing of **3** ^**·**^/**4** ^**·**^.

## 4 Conclusion

This work contributes to the highly‐demanded field of chemistry and materials science focused on stable main‐group radicals and radicaloids [38, 134, 135, 136]. Within the family of 1,3,2‐benzodithiazolys **R** ^**·**^s, the impact of fluorination on crystal and molecular structure, heteroatom reactivity, and solid‐state magnetic properties is studied, expanding the scope of stable open‐shell molecular materials. Through the development of a novel synthetic route involving covalent *σ*‐adducts with TCNQ, highly‐fluorinated **3** ^**·**^ and **4** ^**·**^ radicals —previously elusive— are successfully isolated and structurally characterized. Together with low‐fluorinated **2** ^**·**^, these **R** ^**·**^s exhibit unique reactivity patterns, including reversible transformations into 2H‐1‐oxo‐1,3,2‐benzodithiazoles and selective *π*‐complexation with aromatic donors (e.g., **3** ^**·**^ with naphthalene, cf. complex **12**), highlighting their Lewis ambiphilic character and potential for supramolecular engineering of **3** ^**·**^ and **4** ^**·**^.

The XRD crystal structures of **3** ^**·**^ and **4** ^**·**^ reveal a previously unreported zip‐*π*‐stack supramolecular synthon, combining one lateral *σ*‐dimer and two vertical *π*‐dimers in a single motif. Remarkably, the synthons’ building blocks are also the magnetic building blocks in **3** ^**·**^ and **4** ^**·**^. This packing arrangement leads to a magnetic topology distinct from the eclipsed antiferromagnetically‐coupled *π*‐stack in **2** ^**·**^, as confirmed by first‐principles simulations. Compounds **3** ^**·**^ and **4** ^**·**^ display temperature‐dependent paramagnetic behavior near room temperature despite strong antiferromagnetic coupling. The magnetic response of **3** ^**·**^ and **4** ^**·**^ is further modulated by paramagnetic defects, which must be considered to accurately reproduce the experimental data.

Overall, this study provides new insights into the interplay between molecular structure, supramolecular organization, and magnetic functionality in fluorinated *π*‐radicals. Fluorination is shown to influence not only the thermal stability and heteroatom reactivity of **R** ^**·**^s but also their supramolecular organization and magnetic response. In particular, the presence or absence of eclipsed *π*‐stacking structural motifs and H···F hydrogen bonds appears to play a critical role in enabling bistability in partially fluorinated derivatives. Consequently, in the context of hysteretic room‐temperature magnetic bistability, partially (mono‐, di‐, tri‐) fluorinated **R** ^**·**^s [137], exhibiting H···F hydrogen bonds, are promising candidates for further research. Importantly, partially fluorinated arenes are capable of forming arene–polyfluoroarene *π*‐stacking interactions within their own homocrystals [12, 138, 139]. In contrast, fully hydrocarbon or fully fluorocarbon arenes typically require cocrystallization with complementary species to achieve similar *π*‐stacking arrangements [3]. Heavier halogens also effectively modulate physical properties of solid *π*‐organics [140], particularly magnetic ones via spin–orbit coupling [141], and corresponding derivatives of **R** ^**·**^s are also of interest [137]. Combination of chalcogen‐nitrogen and halogen research is thus expected to be highly fruitful for main group chemistry and materials science.

## Supporting Information

Additional supporting information can be found online in the Supporting Information section. The Supporting Information for this article contains synthetic (**Section S1**), X‐Ray crystallography (**Section S2**), Electrospray Ionization Mass Spectrometry (ESI‐MS, **Section S3**), Nuclear Magnetic Resonance Spectroscopy (NMR, **Section S4**), Infrared and Attenuated Total Reflectance Spectroscopy (IR and ATR, **Section S5**), Ultraviolet‐Visible Spectroscopy (UV‐Vis, **Section S6**), Simultaneous Thermogravimetry – Differential Scanning Calorimetry (TG‐DSC, **Section S7**), electrochemistry (**Section S8**), Electron Paramagnetic Resonance Spectroscopy (EPR, **Section S9**), and SQUID magnetometry (**Section S10**) data, together with crystal packing synthon analysis (**Section S11**), quantitative Hirshfeld surface analysis (**Section S12**), and modeling of magnetic response (**Section S13**).

## Conflicts of Interest

The authors declare no conflicts of interest.

## Supporting information

Supplementary Material

## Data Availability

The data that support the findings of this study are available from the corresponding author upon reasonable request.

## References

[1] S. Dehnen , L. Schafer , T. Lectka , and A Togni , “Fluorine: A Very Special Element and Its Very Special Impact on Chemistry,” Journal of Organic Chemistry 86 (2021): 16213–16219, 10.1021/acs.joc.1c02745. 34806389

[2] R. Berger , G. Resnati , P. Metrangolo , E. Weber , and J. Hulliger , “Organic Fluorine Compounds: A Great Opportunity for Enhanced Materials Properties”, Chemical Society Reviews 40 (2011): 3496–3508, 10.1039/c0cs00221f. 21448484

[3] P. Kirsch , Modern Fluoroorganic Chemistry: Synthesis, Reactivity, Applications, (Wiley‐VCH, 2013), 1–379, 10.1002/9783527651351.

[4] M. G. Campbell , and T. Ritter , “Modern Carbon–Fluorine Bond Forming Reactions for Aryl Fluoride Synthesis”, Chemical Reviews 115 (2015): 612–633, 10.1021/cr500366b. 25474722

[5] A. Harsanyi , and G. Sandford , “Organofluorine Chemistry: Applications, Sources and Sustainability”, Green Chemistry 17 (2015): 2081–2086, 10.1039/c4gc02166e.

[6] D. O’Hagan , “Understanding Organofluorine Chemistry. An Introduction to the C–F Bond”, Chemical Society Reviews 37 (2008): 308–319, 10.1039/b711844a. 18197347

[7] M.‐H. Yoon , A. Facchetti , C. E. Stern , and T. J. Marks , “Fluorocarbon‐Modified Organic Semiconductors: Molecular Architecture, Electronic and Crystal Structure Tuning of Arene‐ versus Fluoroarene‐Thiophene Oligomer Thin‐Film Properties”, Journal of the American Chemical Society 128 (2006): 5792–5801, 10.1021/ja060016a. 16637648

[8] F. Babudri , G. M. Farinola , F. Naso , and R. Ragni , “Fluorinated Organic Materials for Electronic and Optoelectronic Applications: The role of the Fluorine Atom”, Chemical Communications (2007): 1003–1022, 10.1039/b611336b. 17325792

[9] S. Subramanian , S. K. Park , S. R. Parkin , V. Podzorov , T. N. Jackson , and J. E. Anthony , Chromophore Fluorination Enhances Crystallization and Stability of Soluble Anthradithiophene Semiconductors”, Journal of the American Chemical Society 130 (2008): 2706–2707, 10.1021/ja073235k. 18260664

[10] J.‐H. Dou , Y.‐Q. Zheng , Z.‐F. Yao , et al., “Fine‐Tuning of Crystal Packing and Charge Transport Properties of BDOPV Derivatives through Fluorine Substitution”, Journal of the American Chemical Society 137 (2015): 15947–15956, 10.1021/jacs.5b11114. 26619351

[11] R. Ragni , A. Punzi , F. Babudri , and G. M. Farinola , “Organic and Organometallic Fluorinated Materials for Electronics and Optoelectronics: A Survey on Recent Research”, European Journal of Organic Chemistry 2018 (2018): 3500–3519, 10.1002/ejoc.201800657.

[12] P. V. Nikulshin , A. Yu Makarov , I. P. Koskin , et al., “1,2,3,4‐Tetrafluorobiphenylene: A Prototype Janus‐Headed Scaffold for Ambipolar Materials”, ChemPlusChem 89 (2024): e202300692, 10.1002/cplu.202300692. 38052725

[13] C. Trapp , C.‐S. Wang , and R. Filler , “Electron Spin Resonance Absorption in the Tris(pentafluorophenyl)methyl Radical”, The Journal of Chemical Physics 45 (1966): 3472–3474, 10.1063/1.1728145.

[14] W. R. Dolbier , “Fluorinated Free Radicals”, in Topics Current Chemistry, Organofluorine Chemistry (1997), 97–163, 192 10.1007/bfb0119266.

[15] L. V. Polytanskaya , P. A. Fedyushin , T. V. Rybalova , A. S. Bogomyakov , N. V. Asanbaeva , and E. V. Tretyakov , “Fluorinated Organic Paramagnetic Building Blocks for Cross‐Coupling Reactions”, Molecules 25 (2020): 5427, 10.3390/molecules25225427. 33228185 PMC7699513

[16] A. J. Fernandez , R. Giri , K. N. Houk , and D. Katayev , “Review and Theoretical Analysis of Fluorinated Radicals in Direct C_Ar_–H Functionalization of (Hetero)arenes,” Angewandte Chemie International Edition 63 (2024): e202318377, 10.1002/anie.202318377. 38282182

[17] E. V. Tretyakov , and P. A. Fedyushin , “Polyfluorinated Organic Paramagnets,” Russian Chemical Bulletin 70 (2021): 2298–2314, 10.1007/s11172-021-3346-5.

[18] J. M. Rawson , and C. P. Constantinides , “Thiazyl Magnets,” in World Scientific Reference on Spin in Organics, ed. Z. V. Vardeny , M. Wohlgenannt , and J. S. Miller 4 (World Scientific, 2018), 95–124, 10.1142/9789813230200_0002.

[19] J. M. Rawson , and J. J. Hayward , “Reversible Spin Pairing in Crystalline Organic Radicals”, in Spin Crossover Materials: Properties and Applications, ed. M. A. Halcrow , (Wiley, 2013), 225–237, 10.1002/9781118519301.ch8.

[20] J.M. Rawson , F. Palacio , “Magnetic properties of thiazyl radicals”, in π‐Electron Magnetism. Structure and Bonding, ed. J. Veciana , D. Arcon , M. Deumal , K. Inoue , M. Kinoshita , J. J. Novoa , F. Palacio , K. Prassides , J. M. Rawson , and C. Rovira , 100 (Springer, 2001), 93–128, 10.1007/3-540-44684-2_4.

[21] A. Yu Makarov , A. A. Buravlev , G. V. Romanenko , et al., “Hysteretic Room‐Temperature Magnetic Bistability of the Crystalline 4,7‐Difluoro‐1,3,2‐Benzodithiazolyl Radical”, ChemPlusChem 89 (2024): e202300736, 10.1002/cplu.202300736. 38332534

[22] A. A. Buravlev , A. Yu Makarov , O. A. Rakitin , and A. V. Zibarev , “Wolmershäuser Radicals: Chemistry and Materials Science”, Mendeleev Communications 33 (2023): 439–447, 10.1016/j.mencom.2023.06.001.

[23] A. Alberola , D. J. Eisler , L. Harvey , and J. M. Rawson , “Molecular Tailoring of Spin‐Transition Materials: Preparation, Crystal Structure and Magnetism of Trifluoromethyl‐Pyridyl‐1,3,2‐Dithiazolyl”, Crystal Engineering Communications 13 (2011): 1794−1796, 10.1039/c0ce00893a.

[24] P. A. Koutentis , A. Yu Makarov , O. A. Rakitin , and A. V. Zibarev , “Herz Chemistry and its Applications in Small‐Molecule Functional Materials Science: Achievements, Challenges, and Prospects”, Russian Chemical Reviews 94 (2025): RCR5146.6, 10.59761/RCR5146.

[25] A. Mailman , S. M. Winter , X. Yu , et al., “Crossing the Insulator‐to‐Metal Barrier with a Thiazyl Radical Conductor”, Journal of the American Chemical Society 134 (2012): 9886–9889, 10.1021/ja303169y. 22681388

[26] R. G. Higgs , “The Synthesis and Characterization of Stable Radicals Containing the Thiazyl (SN) Fragment and their Use as Building Blocks for Advanced Functional Materials,” in Stable Radicals: Fundamentals and Applied Aspects of Odd‐Electron Compounds, ed. R. G. Higgs (Wiley, 2010), 317–380, 10.1002/9780470666975.ch9.

[27] A. J. Banister , N. Bricklebank , I. Lavender , et al., “Spontaneous Magnetization in a Sulfur‐Nitrogen Radical”, Angewandte Chemie International Edition 35 (1996): 2533–2535, 10.1002/anie.199625331.

[28] R. I. Thomson , C. M. Pask , G. O. Lloyd , M. Mito , and J. M. Rawson , “Pressure‐Induced Enhancement of Magnetic Ordering Temperature in Organic Radical to 70 K: A Magnetostructural Correlation”, Chemistry – A European Journal 18 (2012): 8629–8633, 10.1002/chem.201200760. 22689501

[29] D. A. Gulyaev , N. V. Gadimov , N. P. Gritsan , et al., “Polyfluorinated Blatter Radicals”, Mendeleev Communications 35 (2025): 169–171, 10.71267/mencom.7724.

[30] D. Gulyaev , A. Serykh , D. Gorbunov , et al., “Polyfluorophenyl‐Substituted Blatter Radicals: Synthesis and Structure–Properties Correlations”, Crystal Growth & Design 24 (2024): 5764–5774, 10.1021/acs.cgd.4c00537.

[31] E. Tretyakov , P. Fedyushin , N. Bakuleva , et al., “Series of Fluorinated Benzimidazole‐Substituted Nitronyl Nitroxides: Synthesis, Structure, Acidity, Redox Properties, and Magnetostructural Correlations”, The Journal of Organic Chemistry 88 (2023): 10355–10370, 10.1021/acs.joc.2c01793. 36198196

[32] A. Serykh , E. Tretyakov , P. Fedyushin , et al., “N‐Fluoroalkylpyrazolyl‐Substituted Nitronyl Nitroxides”, Journal of Molecular Structure 1269 (2022): 133739, 10.1016/j.molstruc.2022.133739.

[33] K. Uchida , Y. Hirao , H. Kurata , K. Kubo , S. Hatano , and K. Inoue , “Dual Association Mode of the 2,5,8‐Tris(pentafluorophenyl)phenalenyl Radical”, Chemistry – An Asian Journal 9 (2014): 1823–1829, 10.1002/asia.201402187. 24817683

[34] R. Filler , A. E. Fiebig , and B. K. Mandal , “Fluorocarbanion Chemistry. Tris(4‐nitro‐2,3,5,6‐tetrafluorophenyl) Methane and Companions”, Journal of Fluorine Chemistry 102 (2000): 185–188, 10.1016/S0022-1139(99)00242-0.

[35] C. Shu , Z. Yang , and A. Rajca , “From Stable Radicals to Thermally Robust High‐Spin Diradicals and Triradicals”, Chemical Reviews 123 (2023): 11954–12003, 10.1021/acs.chemrev.3c00406. 37831948

[36] I. Ratera , and J. Veciana , “Playing with Organic Radicals as Building Blocks for Functional Molecular Materials”, Chemical Society Reviews 41 (2012): 303–349, 10.1039/c1cs15165g. 21850355

[37] L. Ji , J. Shi , J. Wei , T. Yu , and W. Huang , “Air‐Stable Organic Radicals: New‐Generation Materials for Flexible Electronics?” Advanced Materials 32 (2020): 1908015, 10.1002/adma.201908015. 32583945

[38] Z. X. Chen , Y. Li , and F. Huang , “Persistent and Stable Organic Radicals”, Chem 7 (2021): 288–332, 10.1016/j.chempr.2020.09.024.

[39] W. Wu , “Stable Organic Radicals – A Material Platform for Developing Molecular Quantum Technologies”, Physical Chemistry Chemical Physics 27 (2025): 1214–1221, 10.1039/d4cp02405b. 39714131

[40] J. M. Rawson , A. Alberola , and A. Whalley , “Thiazyl Radicals: Old Materials for New Molecular Devices”, Journal of Materials Chemistry 16 (2006): 2560–2575, 10.1039/b603199d.

[41] K. E. Preuss , “Metal Complexes of Thiazyl Radicals”, Dalton Transactions (2007): 2357–2369, 10.1039/b704899h. 17844656

[42] D. Leckie , M. Harb , N. Mroz , et al., “A Spontaneous Magnetic Moment in Organic Radical: Synthesis and Characterization of Benzodioxepinyl‐1,3,2‐dithiazolyl”, Journal of the American Chemical Society 146 (2024): 31371–31376, 10.1021/jacs.4c09796. 39509525

[43] T. M. Barclay , A. W. Cordes , N. A. George , et al., “Redox, Magnetic, and Structural Properties of 1,3,2‐Dithiazolyl Radicals. A Case Study on the Ternary Heterocycle S_3_N_5_C_4_ ”, Journal of the American Chemical Society 120 (1998): 352–360, 10.1021/ja973338a.

[44] W. Fujita , and K. Awaga , “Room‐Temperature Organic Bistability in Organic Radical Crystals”, Science 286 (1999): 261–262, 10.1126/science.286.5438.261. 10514363

[45] W. Fujita , and K. Awaga , “Paramagnetic‐Diamagnetic Phase Transition and Magnetic Ordering in Thiazyl Radicals”, Synthetic Metals 137 (2003): 1263–1265, 10.1016/S0379-6779(02)00990-6.

[46] W. Fujita , K. Awaga , Y. Nakazawa , K. Saito , and M. Sorai , “Complex Phase Transition in Stable Thiazyl Radicals: Spin‐Gap, Antiferromagnetic Ordering and Double Melting”, Chemical Physics Letters 352 (2002): 348–352, 10.1016/S0009-2614(01)01477-4.

[47] J. L. Brusso , O. P. Clements , R. C. Haddon , et al., “Bistabilities in 1,3,2‐Dithiazolyl Radicals”, Journal of the American Chemical Society 126 (2004): 8256–8265, 10.1021/ja048618m. 15225068

[48] J. L. Brusso , O. P. Clements , R. C. Haddon , et al., “Bistability and the Phase Transition in 1,3,2‐Dithiazolo[4,5‐*b*]pyrazine‐2‐yl”, Journal of the American Chemical Society 126 (2004): 14692–14693, 10.1021/ja044979q. 15535673

[49] K. Lekin , S. M. Winter , L. E. Downie , et al., “Hysteretic Spin Crossover between a Bisdithiazolyl Radical and Its Hypervalent σ‐Dimer”, Journal of the American Chemical Society 132 (2010): 16212–16224, 10.1021/ja106768z. 20964332

[50] S. Vela , M. B. Reardon , C. E. Jakobsche , M. M. Turnbull , J. Ribas‐Ariño , and J. J. Novoa , “Bistability in Organic Magnetic Materials: A Comparative Study of the Key Differences between Hysteretic and Non‐Hysteretic Spin Transitions in Dithiazolyl Radicals”, Chemistry – A European Journal 23 (2017): 3479–3489, 10.1002/chem.201700021. 28124498

[51] D. Bates , C. M. Robertson , A. A. Leitch , P.A. Dube , and R. T. Oakley , “Magnetic Bistability in Naphtho‐1,3,2‐dithiazolyl: Solid State Interconversion of a Thiazyl π‐Radical and Its N–N σ‐Bonded Dimer”, Journal of the American Chemical Society 140 (2018): 3846–3849, 10.1021/jacs.7b13699. 29513996

[52] A. Paul , A. Gupta , and S. Konar , “Magnetic Transitions in Organic Radicals: The Crystal Engineering Aspects”, Crystal Growth & Design 21 (2021): 5473–5489, 10.1021/acs.cgd.1c00731.

[53] K. Awaga , Y. Umezono , W. Fujita , et al., “Diverse Magnetic and Electrical Properties of Molecular Solids Containing the Thiazyl Radical BDTA”, Inorganica Chimica Acta 361 (2008): 3761–3770, 10.1016/j.ica.2008.03.065.

[54] E.G. Awere , N. Burford , R.C. Haddon , S. Parsons , J. Passmore , J.V. Waszczak , and P.S. White , “X‐Ray Crystal Structures of the 1,3,2‐Benzodithiazolyl Dimer and 1,3,2‐Benzodithiazolium Chloride Sulfur Dioxide Solvate: Comparison of the Molecular and Electronic Structures of the 10‐π‐Electron C_6_H_4_S_2_N^+^ Cation and the C_6_H_4_S_2_N· Radical and Dimer and a Study of the Variable‐Temperature Magnetic Behavior of the Radical”, Inorganic Chemistry 29 (1990): 4821–4830. 10.1021/ic00348a044.

[55] E. G. Awere , N. Burford , C. Mailer , et al., “The High Yield Preparation, Characterization, and Gas Phase Structure of Thermally Stable CF_3_CSNSCCF_3_·, 4,5‐Bis(trifluoromethyl)‐1,3,2‐dithiazolyl and the X‐Ray Crystal Structure of Benzo‐1,3,2‐dithiazolyl”, Journal of the Chemical Society, Chemical Communications (1987): 66–69, 10.1039/c39870000066.

[56] K. Awaga , K. Nomura , H. Kishida , et al., “Electron‐Transfer Processes in Highly Correlated Electron Systems of Thiazyl Radicals”, Bulletin of the Chemical Society of Japan 87 (2014): 234–249. 10.1246/bcsj.20130248.

[57] M. Deumal , S. Vela , M. Fumanal , J. Ribas‐Ariño , and J. J. Novoa , “Insights into the Magnetism and Phase Transitions of Organic Radical‐Based Materials”, Journal of Materials Chemistry C 9 (2021): 10624–10646, 10.1039/D1TC01376A.

[58] G. Wolmershäuser , and R. Johann , “1,3,5‐Trithia‐2,4,6‐triazapentalenyl –a Stable Sulfur‐Nitrogen Radical”, Angewandte Chemie International Edition 28 (1989): 920–921, 10.1002/anie.198909201.

[59] G. D. McManus , J. M. Rawson , N. Feeder , et al., “Synthesis, Crystal Structures, Electronic Structure and Magnetic Behaviour of the Trithiatriazapentalenyl Radical, C_2_S_3_N_3_ ”, Journal of Materials Chemistry 11 (2001): 1992–2003, 10.1039/B103303B.

[60] H. Matsuzaki , W. Fujita , K. Awaga , and H. Okamoto , “Photoinduced Phase Transition in an Organic Radical Crystal with Room‐Temperature Optical and Magnetic Bistability”, Physical Review Letters 91 (2003): 017403, 10.1103/PhysRevLett.91.017403. 12906574

[61] P. Naumov , J. P. Hill , K. Sakurai , M. Tanaka , and K. Ariga , “Structural Study of the Thermally Induced and Photoinduced Phase Transitions of the 1,3,5‐trithia‐2,4,6‐Triazapentalenyl (TTTA) Radical”, The Journal of Physical Chemistry A 111 (2007): 6449, 10.1021/jp072807k. 17580840

[62] S. Vela , F. Mota , M. Deumal , et al., “The Key Role of Vibrational Entropy in the Phase Transitions of Dithiazolyl‐based Bistable Magnetic Materials”, Nature Communications 5 (2014): 4411, 10.1038/ncomms5411. 25014436

[63] S. Vela , M. Deumal , M. Shiga , J. J. Novoa , and J. Ribas‐Ariño , “Dynamical Effects on the Magnetic Properties of Dithiazolyl Bistable materials”, Chemical Science 6 (2015): 2371–2381, 10.1039/C4SC03930K. 29308151 PMC5645919

[64] T. Francese , F. Mota , M. Deumal , et al., “Reorganization of Intermolecular Interactions in the Polymorphic Phase Transition of a Prototypical Dithiazolyl‐Based Bistable Material”, Crystal Growth & Design 19 (2019): 2329–2339, 10.1021/acs.cgd.9b00030.

[65] J. G. Richardson , A. Mizuno , Y. Shuku , et al., “Evaluating the High‐pressure Structural Response and Crystal Lattice Interactions of the Magnetically‐bistable Organic Radical TTTA”, Crystal Engineering Communications 23 (2021): 4444–4450, 10.1039/D1CE00577D.

[66] W. Fujita , and K. Awaga , “Ferromagnetic Coordination Polymer Composed of Heterocyclic Thiazyl Radical, 1,3,5‐Trithia‐2,4,6‐triazapentalenyl (TTTA), and Bis(hexafluoroacetylacetonato) copper(II) (Cu(hfac)_2_)”, Journal of the American Chemical Society 123 (2001): 3601–3602, 10.1021/ja002873z. 11472135

[67] In this work, β‐**1**· exhibiting chiral space group P2_1_2_1_2_1_ was characterized by single‐crystal XRD (will be published elsewhere); α‐**1**· crystallizes in the space group Pbca (ref. 54). The β‐**1**· was obtained by vacuum sublimation of unreacted mixture of α‐**1**· with octafluoronaphthalene.

[68] G. R. Desiraju , “Supramolecular Synthons in Crystal Engineering –A New Organic Synthesis”, Angewandte Chemie International Edition 34 (1995): 2311–2327, 10.1002/anie.199523111.

[69] O. V. Shishkin , R. I. Zubatyuk , S. V. Shishkina , V. V. Dyakonenko , and V. V. Medviediev , “Role of Supramolecular Synthons in the Formation of the Supramolecular Architecture of Molecular Crystals Revisited from an Eenergetic Viewpoint,” Physical Chemistry Chemical Physics 16 (2014): 6773–6786, 10.1039/C3CP55390F. 24595277

[70] A. Pizzi , G. Terraneo , C. Lo Iacono , R. Beccaria , A. Dhaka , G. Resnati , “Taxonomy of Chemical Bondings: Opportunities and Challenges”, Angewandte Chemie International Edition 64 (2025): e2025065525, 10.1002/anie.202506525. PMC12207382 40401347

[71] A. G. Orpen , “Secondary Bonding as a Potential Design Tool for Crystal Engineering,” in Crystal Engineering: From Molecules to Crystals and Materials, eds. D. Braga , F. Grepioni , and A. G. Orpen (Springer, 1999), 107–127, 10.1007/978-94-011-4505-3.

[72] T. M. Barclay , A. W. Cordes , N. A. George , et al., “Molecular Materials from 1,3,2‐dithiazolyls. Solid‐State Structures and Magnetic Properties of 2,3‐naphthalene and Quinoxaline Derivatives”, Chemical Communication (1997): 873–874, 10.1039/a700310b.

[73] A. Alberola , D. Eisler , R. J. Less , E. Navarro‐Moratalla , and J. M. Rawson , “Synthesis and Characterisation of 3,4‐dialkoxy‐substituted benzo‐1,3,2‐dithiazolyl Radicals”, Chemical Communication 46 (2010): 6114–6116, 10.1039/c0cc01282c. 20657916

[74] G. D. McManus , J. M. Rawson , N. Feeder , F. Palacio , and P. Oliete , “Structure and Magnetic Properties of a Sulfur‐Nitrogen Radical, Methylbenzodithiazolyl”, Journal of Materials Chemistry 10 (2000): 2001–2003, 10.1039/b004992l.

[75] T. M. Barclay , A. W. Cordes , and R. H. de Laat , , “The Heterocyclic Diradical Benzo‐1,2:4,5‐bis(1,3,2‐dithiazolyl). Electronic, Molecular and Solid State Structure”, Journal of the American Chemical Society 119 (1997): 2633–2641, 10.1021/ja9636294.

[76] P. A. Champagne , J. Desroches , and J.‐F. Paquin , “Organic Fluorine as a Hydrogen‐Bond Acceptor: Recent Examples and Applications“, Synthesis 47 (2015): 306–322, 10.1055/s-0034-1379537

[77] H.‐F. Schneider , “Hydrogen bonds with fluorine. Studies in solution, in gas phase and by computations, conflicting conclusions from crystallographic analyses”, Chemical Science 3 (2012): 1381–1394, 10.1039/c2sc00764a.

[78] J. D. Dunitz , and R. Taylor , “Organic Fluorine Hardly Ever Accepts Hydrogen Bonds”, ChemistryA Europen Journal 3 (1997): 89–98, 10.1002/chem.19970030115.

[79] J. A. K. Howard , V. J. Hoy , D. O’Hagan , and G. T. Smith , “How good is fluorine as a hydrogen bond acceptor?”, Tetrahedron 52 (1996): 12613–12622, 10.1016/0040-4020(96)00749-1.

[80] T.V. Rybalova , and I. Yu. Bagryanskaya , “C‐F···π, F···H, and F···F intermolecular interactions and F‐aggregation: Role in crystal engineering of fluoroorganic compounds”, Journal of Structural Chemistry 50 (2009): 741–753, 10.1007/s10947-009-0113-0.

[81] P. R. Spackman , M. J. Turner , J. J. McKinnon , et al., “CrystalExplorer: a program for Hirshfeld surface analysis, visualization and quantitative analysis of molecular crystals”, Journal of Applied Crystallography 54 (2021): 1006–1011, 10.1107/S1600576721002910. 34188619 PMC8202033

[82] C. F. Mackenzie , P. R. Spackman , D. Jayatilaka , and M. A. Spackman , “CrystalExplorer model energies and energy frameworks: extension to metal coordination compounds, organic salts, solvates and open‐shell systems”, IUCrJ 4 (2017): 575–587, 10.1107/S205225251700848X. PMC5600021 28932404

[83] S. L. Tan , M. M. Jotani , and E. R. T. Tiekink , “Utilizing Hirshfeld Surface Calculations, Non‐covalent Interaction (NCI) Plots and the Calculation of Interaction Energies in the Analysis of Molecular Packing”, Acta Crystallographic E 75 (2019): 308–318, 10.1107/S2056989019001129. PMC6399703 30867939

[84] T. Lu , “Visualization Analysis of Covalent and Noncovalent Interactions in Real Space”, Angewandte Chemie Internation Edition 64 (2025): e202504895, 10.1002/anie.202504895. 40323713

[85] I. Yu. Bagryanskaya , H. Bock , Yu. V. Gatilov , et al., “Cyclic Aryleneazachalcogenes. 10. Synthesis, Molecular Structure and Photoelectron Spectrum of 6,7,8,9‐tetrafluoro‐1,3,5,2,4‐benzotrithiazepine and Attempted Syntheses of Related Larger Size Heterocycles”, Chemische Berichte/Recueil 130 (1997): 247–253, 10.1002/cber.19971300218.

[86] G. Wolmershäuser , M. Schnauber , and T. Wilhelm , “Highly Conducting Charge‐Transfer Complexes of Benzo‐1,3,2‐dithiazol‐2‐yl and its derivatives with Tetracyanoquinodimethane”, Journal of Chemical Society, Chemical Communication (1984): 573–574, 10.1039/c39840000573.

[87] V. D. Tikhova , V. P. Fadeeva , O. N. Nikulicheva , T. A. Dobinskaya , and Yu. M. Deryabina , “Determination of Fluorine in Organic Functional Materials”, Chemistry for Sustainable Development 30 (2022): 640–653, 10.15372/CSD2022427.

[88] A. Alberola , G. D. McManus , and J. M. Rawson , “Synthesis and characterisation of tetrafluorobenzo‐1,3,2‐dithiazolyl”, Phosphorus Sulfur Silicon Related Elements 179 (2004): 979–980, 10.1080/10426500490429572.

[89] A. A. Buravlev , A. Yu. Makarov , G. E. Salnikov , et al., “Synthesis, structural peculiarities, and photosensitivity of fluorinated dibenzo‐1,2,5,6‐tetrathiocines”, New Journal of Chemistry 48 (2024): 12807–12816, 10.1039/d4nj02284j.

[90] T. Chivers , M. Parvez , I. Vargas‐Baca , and G. Schatte , “Conformational isomers of 1,2,5,6‐tetrathiocins and the photoisomerization of a 1,2,5,6‐tetrathiocin into a 1,2,3,6‐tetrathiocin: X‐ray structures of (C_6_X_4_S_2_)_2_ (X = F, Cl) and C_6_F_4_SSSC_6_F_4_S”, Canadian Journal of Chemistry 76 (1998): 1093–1101, 10.1139/cjc-76-7-1093.

[91] E. A. Radiush , H. Wang , E. A. Chulanova , et al., “Recognition and sensing of Lewis bases by 1,2,5‐chalcogenadiazoles”, Mendeleev Communications 34 (2024): 297–306, 10.1016/j.mencom.2024.04.001.

[92] G. M. Sheldrick , ”Crystal structure refinement with *SHELXL*”, Acta Crystallographica C 71 (2015): 3–8, 10.1107/S2053229614024218. PMC4294323 25567568

[93] G. M. Sheldrick , SADABS, v. 2008‐1; Bruker AXS Area Detector Scaling and Absorption Correction, (Bruker AXS, 2008).

[94] C. F. Macrae , P. R. Edgington , P. McCabe , et al., “ *Mercury*: visualization and analysis of crystal structures”, Journal of Applied Crystallography 39 (2006): 453–457, 10.1107/S002188980600731X.

[95] *X‐AREA*; STOE & Cie GmbH, 2013.

[96] R. D. Duling , “Simulation of Multiple Isotropic Spin‐Trap EPR‐Spectra”, Journal of Magnetic Resonance 104 (1994): 105–110, 10.1006/jmrb.1994.1062. 8049862

[97] F. Neese , “Software update: The ORCA program system‐Version 5.0”, Wiley Interdisciplinary Reviews: Computational Molecular Science 12 (2022): e1606, 10.1002/wcms.1606.

[98] M. J. Frisch , G. W. Trucks , H. B. Schlegel , et al., Gaussian 09, (Gaussian Inc., 2009).

[99] A. D. Laurent , and D. Jacquemin , “TD‐DFT Benchmarks: A Review”, International Journal of Quantum Chemistry 113 (2013): 2019–2039, 10.1002/qua.24438.

[100] M. L. Laury , S. E. Boesch , I. Haken , P. Sinha , R. A. Wheeler , and A. K. Wilson , “Harmonic Vibrational Frequencies: Scale Factors for Pure, Hybrid, Hybrid Meta, and Double‐Hybrid Functionals in Conjunction with Correlation Consistent Basis Sets”, Journal of Computation Chemistry 32 (2011): 2339–2347, 10.1002/jcc.21811. 21598273

[101] J. Tomasi , B. Mennucci , and R. Cammi , “Quantum mechanical continuum solvation models”, Chemical Reviews 105 (2005): 2999–3093, 10.1021/cr9904009. 16092826

[102] I. Sandler , J. Chen , M. Taylor , S. Sharma , and J. Ho , “Accuracy of DLPNO‐CCSD(T): Effect of Basis Set and System Size”, Journal of Physical Chemistry A 125 (2021): 1553–1563, 10.1021/acs.jpca.0c11270. 33560853

[103] P. Giannozzi , O. Andreussi , T. Brumme , et al., “Advanced capabilities for materials modelling with QUANTUM ESPRESSO”, Journal of Physics: Condensed Matter 29 (2017): 465901, 10.1088/1361-648X/aa8f79. 29064822

[104] S. Grimme , J. Antony , S. Ehrlich , and H. Krieg , “A consistent and accurate ab initio parametrization of density functional dispersion correction (DFT‐D) for the 94 elements H‐Pu”, Journal of Chemical Physics 132 (2010): 154104, 10.1063/1.3382344. 20423165

[105] L. Goerigk , “A Comprehensive Overview of the DFT‐D3 London‐Dispersion Correction,” in Non‐Covalent Interactions in Quantum Chemistry and Physics, eds. A. Oterode la Roza and G. A. DiLabio (Elsevier, 2017), 195–219, 10.1016/C2015-0-06383-3.

[106] D. Vanderbilt , “Soft Self‐Consistent Pseudopotentials in a Generalized Eigenvalue Formalism”, Physical Review B 41 (1990): 7892–7895, 10.1103/PhysRevB.41.7892. 9993096

[107] M. Deumal , M. J. Bearpark , J. J. Novoa , and M. A. Robb , “Magnetic Properties of Organic Molecular Crystals Via an Algebraic Heisenberg Hamiltonian. Applications to WILVIW, TOLKEK, and KAXHAS Nitronyl Nitroxide Crystals”, Journal of Physical Chemistry A 106 (2002): 1299‐1315, 10.1021/jp015512u.

[108] J. J. Novoa , M. Deumal , and J. Jornet‐Somoza , “Calculation of microscopic exchange interactions and modelling of macroscopic magnetic properties in molecule‐based magnets”, Chemical Society Reviews 40 (2011): 3182–3212, 10.1039/c0cs00112k. 21321725

[109] J. Jornet , M. A. Robb , M. Deumal , and J. J. Novoa , “A First‐Principles Bottom‐up Study of the Magnetic Interaction Mechanism in the Bulk Ferromagnet p‐O_2_N‐C_6_F_4_‐CNSSN”, Inorganica Chimica Acta 361 (2008): 3586–3592, 10.1016/j.ica.2008.03.062.

[110] F. Neese , “Definition of Corresponding Orbitals and the Diradical Character in Broken Symmetry DFT Calculations on Spin Coupled Systems”, Journal of Physics and Chemistry of Solids 65 (2004): 781–785, 10.1016/j.jpcs.2003.11.015.

[111] J. P. Malrieu , R. Caballol , C. J. Calzado , C. de Graaf , and N. Guihèry , “Magnetic interactions in molecules and highly correlated materials: physical content, analytical derivation, and rigorous extraction of magnetic Hamiltonians”, Chem. Rev. 114 (2014): 429–492, 10.1021/cr300500z. 24102410

[112] N. Iwahara , Z. Huang , A. Mansikkamäki , and L. F. Chibotaru , “Breakdown of broken‐symmetry approach to exchange interaction”, Journal of Chemical Physics 162 (2025): 164701, 10.1063/5.0255897. 40260816

[113] M. Fumanal , J. Jornet‐Somoza , S. Vela , J. J. Novoa , J. Ribas‐Ariño , and M. Deumal , “Pitfalls on Evaluating Pair Exchange Interactions for Modelling Molecule‐Based Magnetism”, Jounal of Materials Chemistry C 9 (2021): 10647–10660, 10.1039/D1TC01083B.

[114] R.T. Boerè , and T.L. Roemmele , “Electrochemistry of Redox‐Active Group 15/16 Heterocyles”, Coordination Chemistry Reviews 210 (2000): 369–445, 10.1016/S0010-8545(00)00349-0.

[115] T. M. Barclay , A. W. Cordes , R. C. Haddon , et al., “Preparation and Characterization of a Neutral π‐radical Molecular Conductor”, Journal of the American Chemical Society 121 (1999): 969–976, 10.1021/ja983490s.

[116] V. A. Starodub , and T. N. Starodub , “Radical Anion Salts and Charge Transfer Complexes Based on Tetracyanoquinodimethane and other Potent π‐Electron Acceptors”, Russian Chemical Reviews 83 (2014): 391–438, 10.1070/RC2014v083n05ABEH004299.

[117] K. Andersson , “Molecular Properties of TCNQ and Anions”, Theoretical Chemistry Accounts 142 (2023): 58, 10.1007/s00214-023-02998-7.

[118] A. Taponen , A. Ayadi , M. K. Lahtinen , et al., “Room‐Temperature Magnetic Bistability in a Salt of Organic Radical Ions”, Journal of the American Chemical Society 143 (2021): 15912–15917, 10.1021/jacs.1c07468. 34547207

[119] J. F. Liebman , P. Politzer , and D. C. Rosen , “The π‐Fluoro Effect: An Empirical Use of Atomic Electrostatic Potentials,” in Chemical Applications of Atomic and Molecular Electrostatic Potentials, eds. P. Politzer , D. G. Truhlar , (Springer, 1981), 295–308, 10.1007/978-1-4757-9634-6.

[120] B. P. Bespalov , and V. V. Titov , “7,7,8,8‐Tetracyanoquinodimethane in Addition, Substitution, and Complex Formation Reactions”, Russian Chemical Reviews 44 (1975): 1091–1108, 10.1070/RC1975v044n12ABEH002559.

[121] E. A. Chulanova , E. A. Pritchina , L. A. Malaspina , et al., “New Charge‐Transfer Complexes with 1,2,5‐Thiadiazoles as Both Electron Acceptors and Donors Featuring an Unprecedented Addition Reaction”, Chemistry–A European Journal 23 (2017): 852–864, 10.1002/chem.201604121 27958639

[122] F. H. Allen , O. Kennard , D. G. Watson , L. Brammer , A. G. Orpen , and R. Taylor , “Tables of bond lengths determined by X‐ray and neutron diffraction. Part 1. Bond lengths in organic compounds”, Jounral of the Chemical Society, Perkin Transaction 2 (1987): S1–S19, 10.1039/P298700000S1.

[123] G. Wolmershäuser , and G. Kraft , “N‐Substituted 1,3,2‐Dithiazoles – Precursors for 1,3,2‐Dithiazol‐2‐yl Radicals”, Chemische Berichte 122 (1989): 385–387, 10.1002/cber.19891220230.

[124] G. Wolmershäuser , W. Kaim , G. Heckmann , and A. Lichtblau , “Benzo‐1,3,2‐diselenazolylium Salts –Synthesis and Reactivity”, Zeitschrift für Naturforschung B 47 (1992): 675–679, 10.1515/znb-1992-0511.

[125] L. L. Klein , C. M. Yeung , D. E. Weissing , P. A. Lartey , S. K. Tanaka , J. J. Plattner , D. Mulford , “Synthesis and Antifungal Activity of 1,3,2‐Benzodithiazole S‐Oxide”, Journal of Medicinal Chemistry 37 (1994): 572–578, 10.1021/jm00031a005. 8126696

[126] E. A. Chulanova , E. A. Radiush , I. K. Shundrina , et al., “Lewis Ambiphilicity of 1,2,5‐Chalcogenadiazoles for Crystal Engineering: Complexes with Crown Ethers”, Crystal Growth & Design 20 (2020): 5868–5879, 10.1021/acs.cgd.0c00536.

[127] O. A. Rakitin , and A. V. Zibarev , “Synthesis and Applications of 5‐Membered Chalcogen‐Nitrogen π‐Heterocycles with Three Heteroatoms”, Asian Jounal of Organic Chemistry 7 (2018): 2397–2416, 10.1002/ajoc.201800536.

[128] A. F. Cozzolino , P. J. W. Elder , L. M. Lee , and I. Vargas‐Baca , “The Role of the Lewis Acid−Base Properties in the Supramolecular Association of 1,2,5‐Chalcogenadiazoles”, Canadian Journal of Chemistry 91 (2013): 338–347, 10.1139/cjc-2012-0323.

[129] S. Alvarez , “A cartography of the Van Der Waals Territories”, Dalton Transication 42 (2013): 8617–8636, 10.1039/C3DT50599E. 23632803

[130] The ΔH values were determined using only spin contents assuming that the volume of samples did not change significantly in the 270‐310 K range. Therefore, these ΔH values are not exact thermodynamic since the corresponding activity coefficients are unknown.

[131] P. Ho , J. Z. Wang , F. Meloni , and I. Vargas‐Baca , “Chalcogen Bonding in Materials Chemistry”, Coordination Chemistry Reviews 422 (2020): 213464, 10.1016/j.ccr.2020.213464.

[132] A. Alberola , R. J. Collis , S. M. Humphrey , R. J. Less , and J. M. Rawson , “Spin Transitions in a Dithiazolyl Radical: Preparation, Crystal Structures and Magnetic Properties of 3‐Cyanobenzo‐1,2,3‐dithiazolyl, C_7_H_3_S_2_N_2_•”, Inorganic Chemistry 45 (2006): 1903–1905, 10.1021/ic051935p. 16499348

[133] A. Decken , A. Mailman , J. Passmore , J. M. Rautiainen , W. Scherer , and E.‐W. Scheidt , “A Prototype Hybrid 7π Quinone‐Fused 1,3,2‐Dithiazolyl Radical”, Dalton Transactions 40 (2011): 868–879, 10.1039/c0dt00967a. 21152519

[134] Z. Feng , S. Tang , and W. Wang , “Recent Advances in Stable Main Group Element Radicals: Preparation and Characterization”, Chemical Society Review 51 (2022): 5930–5973, 10.1039/D2CS00288D. 35770612

[135] A. Hinz , J. Bresein , F. Beher , and A. Schulz , “Heteroatom‐Based Diradical(oid)s”, Chemical Review 123 (2023): 10468–10526, 10.1021/acs.chemrev.3c00255. 37556842

[136] P. P. Power , “Persistent and Stable Radicals of the Heavier Main Group Elements and Related Species”, Chemical Review 103 (2003): 789–810, 10.1021/cr020406p. 12630853

[137] Building on this work, 4‐F and 5‐Cl derivatives of 1· have been synthesized and characterized by EPR and XRD; details will be published elsewhere.

[138] I. Yu. Bagryanskaya , Yu. V. Gatilov , A. M. Maksimov , V. E. Platonov , and A. V. Zibarev , “Supramolecular Synthons in Crystals of Partially Fluorinated Fused Aromatics: 1,2,3,4‐Tetrafluoronaphthalene and its Aza‐Analogue 1,3,4‐Trifluoroisoquinoline”, Journal of Fluorine Chemical 126 (2005): 1281–1287, 10.1016/j.jfluchem.2005.06.011.

[139] F. Cozzi , S. Bacchi , G. Filippini , T. Pilati , and A. Gavezzotti , “Synthesis, X‐ray Diffraction and Computational Study of the Crystal Packing of Polycyclic Hydrocarbons Featuring Aromatic and Perfluoroaromatic Rings Condensed in the Same Molecule: 1,2,3,4‐Tetrafluoronaphthalene, ‐anthracene and ‐phenanthrene”, Chemistry‐A European Journal 13 (2007): 7177–7184, 10.1002/chem.200700267. 17568459

[140] M. L. Tang , and Z. Bao , “Halogenated Materials as Organic Semiconductors”, Chemistry of Materials 23 (2011): 446–455, 10.1021/cm102182x.

[141] J. Marbey , A. Mailman , R. T. Oakley , S. Hill , and S. M. Winter , “Substituent Effects on Exchange Anisotropy in Single‐ and Multiorbital Organic Radical Magnets”, Physical Review Materials 8 (2024): 044406, 10.1103/PhysRevMaterials.8.044406.

