# Supplementary material for: Stable 1,3,2‐Benzodithiazolyl Radicals: Modification of Reactivity, Crystal Packing, and Solid State Magnetic Properties by Fluorination
Source: ChemistryOpen. 2026 Feb 26;15(3):e202500561. doi: 10.1002/open.202500561 (PMC12936988; doi:10.1002/open.202500561)
Supplement: Supplementary file 1 — Supplementary Material [file OPEN-15-e202500561-s001.pdf]

Supporting Information

Stable 1,3,2-Benzodithiazolyl Radicals: Modification of Reactivity, Crystal Packing and Solid State Magnetic Properties by Fluorination

Alexander A. Buravlev,[a,d] Alexander Yu. Makarov,*[a] Jordi Ribas-Ariño,[b] M.Àngels Carvajal,[b] Mercè Deumal,*[b] Yaser Balmohammady,[c] Simon Grabowsky,*[c] Inna K. Shundrina,[a] Boris A. Zakharov,[d,g] Irina G. Irtegova,[a] Mikhail N. Uvarov,[e] Artem S. Bogomyakov,[f] Irina Yu. Bagryanskaya,[a] Leonid A. Shundrin,[a] and Andrey V. Zibarev*[a]

* e-mail: makarov@nioch.nsc.ru, merce.deumal@ub.edu, simon.grabowsky@unibe.ch, zibarev@nioch.nsc.ru

a *Institute of Organic Chemistry, Siberian Branch of the Russian Academy of Sciences, 630090 Novosibirsk, Russia*

b *Departament de Ciència de Materials i Química Física & Institut de Química Teòrica i Computacional (IQTC), Universitat de Barcelona, c/ Martí i Franquès 1-11, 08028 Barcelona, Spain*

c *Department of Chemistry, Biochemistry, and Pharmaceutical Sciences, University of Bern, 3012 Bern, Switzerland*

d *Department of Natural Sciences, National Research University – Novosibirsk State University, 630090 Novosibirsk, Russia*

e *Institute of Chemical Kinetics and Combustion, Siberian Branch of the Russian Academy of Sciences, 630090 Novosibirsk, Russia*

f *International Tomography Center, Siberian Branch of the Russian Academy of Sciences, 630090 Novosibirsk, Russia*

g *Synchrotron Radiation Facility – Siberian Circular Source of Photons (SRF «SKIF»), Siberian Branch of the Russian Academy of Sciences, 630055 Koltsovo*

Contents

S1. Chemistry

S2. X-Ray Crystallography

S3. Electrospray Ionization Mass Spectrometry (ESI-MS)

S4. Nuclear Magnetic Resonance (NMR) Spectroscopy

S5. Infrared (IR) and Attenuated Total Reflectance (ATR) Spectroscopy

S6. Ultraviolet-Visible (UV-Vis) Spectroscopy

S7. Simultaneous Thermogravimetry – Differential Scanning Calorimetry (TG-DSC)

S8. Electrochemistry

S9. Electron Paramagnetic Resonance (EPR) Spectroscopy

S10. Magnetometry

S11. Crystal Packing Synthon Analysis

S12. Hirshfeld Surface Analysis

S13. Magnetic response

S14. References and notes

S1. CHEMISTRY

Synthesis of 3,4,5,6-Tetrafuorobenzene-1,2-bis(sulfenyl chloride).

At –70 °C and under argon, 7.6 mL of 2.6 M solution of BuLi in hexane were added to a solution of 2.96 g (19.7 mmol) of 1,2,3,4-tetrafuorobenzene in 75 mL of THF, and the reaction mixture was kept at –65 °C for 30 min. Then 0.63 g (19.7 mmol) of finely powdered sulfur was added, and the reaction mixture was gradually warmed-up to –30 °C during 40 min. After that, it was repeatedly cooled to –70 °C, and 7.6 mL of 2.6 M solution of BuLi in hexane were added. The mixture was kept at –65 °C for 30 min. and 25 mL of THF were added following by 0.63 g (19.7 mmol) of sulfur powder. The reaction mixture was gradually warmed-up to room temperature and quenched with 20 mL of H_2O following by 30 mL of a 4N aqueous HCl. The aqueous layer was siphoned off with a pipette, and the organic layer washed with 2 × 15 mL of H_2O. The combined aqueous solution was extracted with 100 mL of Et_2O, and combined organic solution dried over $MgSO_4$ and evaporated. The residue was distilled at 80 °C / 10 Torr, the product was dissolved in 25 mL of CCl_4, and chlorine gas was passed through the solution for 3.5 h. The solvent was distilled off, and 3,4,5,6-tetrafuorobenzene-1,2-bis(sulfenyl chloride) (3.11 g, 56%) was obtained as orange-red oil. ^{19}F NMR ($CDCl_3$), δ, ppm: 41.6 (m, 2F), 16.9 (m, 2F); (CCl_4): 41.4, 16.5.[S1]

S2. X-RAY CRYSTALLOGRAPHY

Table S1. Crystallographic data of compounds **3**$^{\cdot}$, **4**$^{\cdot}$, **6,** and **9–12**

Compound	**3**$^{\cdot}$	**4**$^{\cdot}$	**6**
Empirical formula	$C_6F_4NS_2$	$C_9F_8NS_2$	$C_{24}H_8F_4N_6S_4$
Formula weight	226.19	338.22	584.60
Temperature K	200(2)	200(2)	296(2)
Wavelength Å	0.71073	0.71073	0.71073
Crystal system	Monoclinic	Monoclinic	Triclinic
Space group	$P2_1/c$	$P2_1/c$	P–1
Unit cell dimensions *a* Å	5.9453(6)	5.9027(6)	7.736(5)
b Å	9.1546(6)	12.9581(9)	7.757(5)
c Å	13.5171(11)	14.6026(13)	11.390(9)
α °	90	90	90.54(3)
β °	90.911(3)	92.527(5)	95.59(3)
γ °	90	90	119.34(2)
Volume $Å^3$	735.60(11)	1115.83(17)	591.7(7)
Z	4	4	1
Density (calcd.) Mg m^{-3}	2.042	2.013	1.641
Abs. coefficient mm^{-1}	0.739	0.573	0.462
F(000)	444	660	294
Crystal size mm^3	0.10 × 0.20 × 0.60	0.05 × 0.10 × 0.20	0.02 × 0.05 × 0.20
Θ range for data collection °	3.0–27.5	3.2–26.0	3.0–25.6
Index ranges	$-7 \le h \le 7, -11 \le k \le 11, -17 \le l \le 17$	$-7 \le h \le 7, -15 \le k \le 10, -18 \le l \le 18$	$-9 \le h \le 8, -9 \le k \le 9, -13 \le l \le 13$
Reflections collected	9971	11470	6174
Independent reflections	1692 R(int) = 0.025	2188 R(int)= 0.070	2179 R(int) = 0.053
Completeness to θ %	99.8	99.7	99.7
Data / restraints / parameters	1692 / 0 / 118	2188 / 15 / 245	2179/ 0 / 172
Goodness-of-fit on F^2	1.03	1.03	1.02
Final R indices $I > 2\sigma(I)$	R_1 = 0.0294, wR_2 = 0.0760	R_1 = 0.0409, wR_2 = 0.0864	R_1 = 0.0439, wR_2 = 0.0848
Final R indices (all data)	R_1 = 0.0367, wR_2 = 0.0840	R_1 = 0.0735, wR_2 = 0.1000	R_1 = 0.0853, wR_2 = 0.0956
Largest diff. peak / hole e $Å^{-3}$	0.36 / –0.27	0.28 / –0.28	0.28 / –0.27
**CCDC**	**2452853**	**2452854**	**2452855**

Table S1 *(continued)*

Compound	**9**	**10**	**11**	**12**
Empirical formula	$C_6H_3F_2NOS_2$	$C_6HF_4NOS_2$	$C_9HF_8NOS_2$	$C_{16}H_8F_4NS_2$
Formula weight	207.21	243.20	355.23	354.35
Temperature K	296(2)	296(2)	296(2)	200(2)
Wavelength Å	0.71073	0.71073	0.71073	0.71073
Crystal system	Monoclinic	Orthorhombic	Monoclinic	Triclinic
Space group	$P2_1/n$	Pccn	$P2_1/c$	P–1
Unit cell dimensions *a* Å	5.0698(3)	13.5764(13)	10.1704(12)	6.824(4)
b Å	15.4404(10)	14.8231(11)	8.0977(7)	7.921(4)
c Å	9.4798(6)	8.1621(10)	14.2508(16)	14.063(7)
α °	90	90	90	85.093(16)
β °	97.502(3)	90	101.763(4)	88.720(16)
γ °	90	90	90	76.749(16)
Volume $Å^3$	735.72(8)	1642.6(3)	1149.0(2)	737.1(7)
Z	4	8	4	2
Density (calcd.) Mg m^{-3}	1.871	1.967	2.054	1.596
Abs. coefficient mm^{-1}	0.701	0.678	0.568	0.401
F(000)	416	960	696	358
Crystal size mm^3	0.04 × 0.10 × 0.50	0.20 × 0.1 × 0.05	0.01 × 0.06 × 0.50	0.02 × 0.05 × 0.15
Θ range for data collection °	3.4–26.1	3.2–26.1	2.9–25.1	1.5–25.1
Index ranges	$-6 \le h \le 5, -19 \le k \le 18, -10 \le l \le 11$	$-13 \le h \le 16, -17 \le k \le 18, -7 \le l \le 10$	$-12 \le h \le 12, -9 \le k \le 7, -16 \le l \le 16$	$-8 \le h \le 8, -9 \le k \le 9, -16 \le l \le 16$
Reflections collected	5327	7096	10047	7534
Independent reflections	1458 R(int) = 0.050	1622 R(int) = 0.065	2042 R(int) = 0.096	2596 R(int) = 0.143
Completeness to θ %	99.8	99.8	99.6	99.9
Data / restraints / parameters	1458 / 0 / 112	1622 / 0 / 130	2042 / 0 / 212	2596 / 0 / 208
Goodness-of-fit on F^2	1.03	1.01	1.04	1.06
Final R indices $I > 2\sigma(I)$	R_1 = 0.0385, wR_2 = 0.0900	R_1 = 0.0430, wR_2 = 0.0955	R_1 = 0.0599, wR_2 = 0.1190	R_1 = 0.1043, wR_2 = 0.2448
Final R indices (all data)	R_1 = 0.0516, wR_2 = 0.0971	R_1 = 0.0727, wR_2 = 0.1092	R_1 = 0.1155, wR_2 = 0.1408	R_1 = 0.2501, wR_2 = 0.2991
Largest diff. peak / hole e $Å^{-3}$	0.31 / –0.24	0.32 / –0.24	0.62 / –0.57	0.67 / –0.40
**CCDC**	**2452857**	**2452858**	**2452859**	**2452856**

Powder XRD indicates the thermally-induced amorphization / irreversible disintegration of the crystal lattices of **3**$^{\cdot}$ and **4**$^{\cdot}$ (see Figure S1).

3$^{\cdot}$ **4**$^{\cdot}$

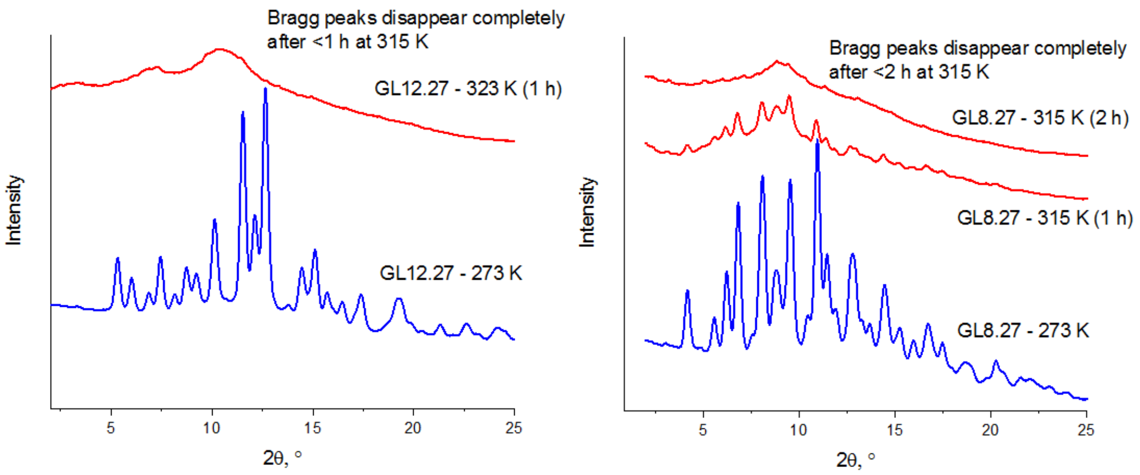

Figure S1. Thermally-induced amorphization of **3**$^{\cdot}$ and **4**$^{\cdot}$ according to powder XRD

S3. ELECTROSPRAY IONIZATION MASS SPECTROMETRY (ESI-MS)

Adduct **6** ($C_{24}H_8F_4N_6S_4$, formula mass 583.962 Da). A sample was prepared directly before measurements as solution in CH_3CN with concentration ~2.2 mg mL^{-1}. Ions **6**$^+$, [**6**-Na]$^+$ or [**6**-K]$^+$, and **6**$^-$ were not observed. Instead, intense peaks with m/z = 189.959 corresponding with the error lesser than 0.001 Da (5.2 ppm) to **2**$^+$ ($C_6H_2F_2NS_2$), and m/z = 204.044 corresponding with the error lesser than 0.001 Da (4.9 ppm) to TCNQ$^{\cdot-}$ ($C_{12}H_4N_4$), respectively, were detected, both exhibiting correct isotopic distributions (Figures S2 and S3).

(a)

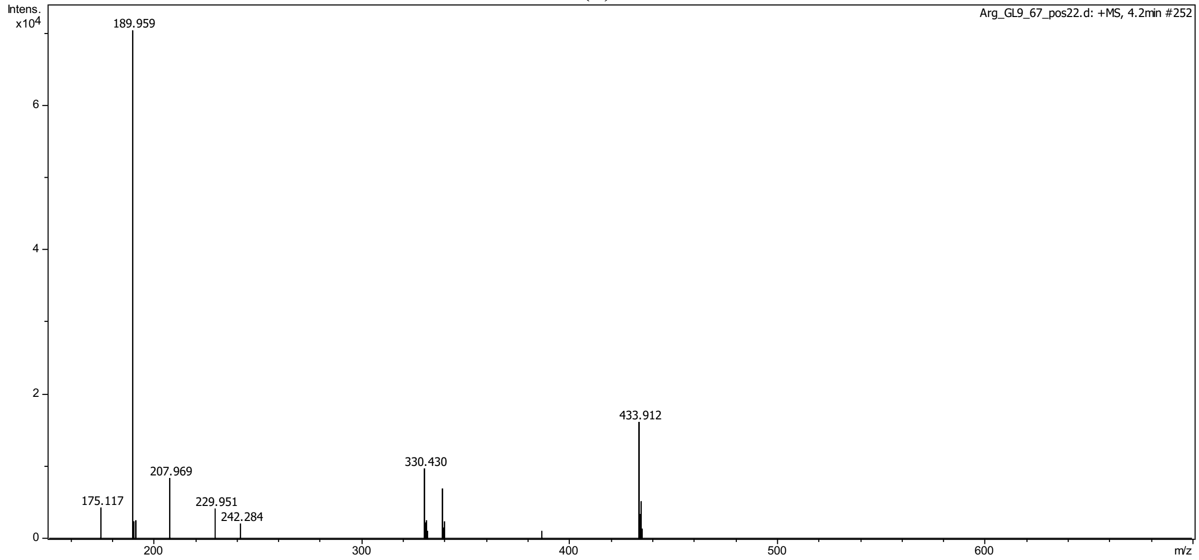

(b)

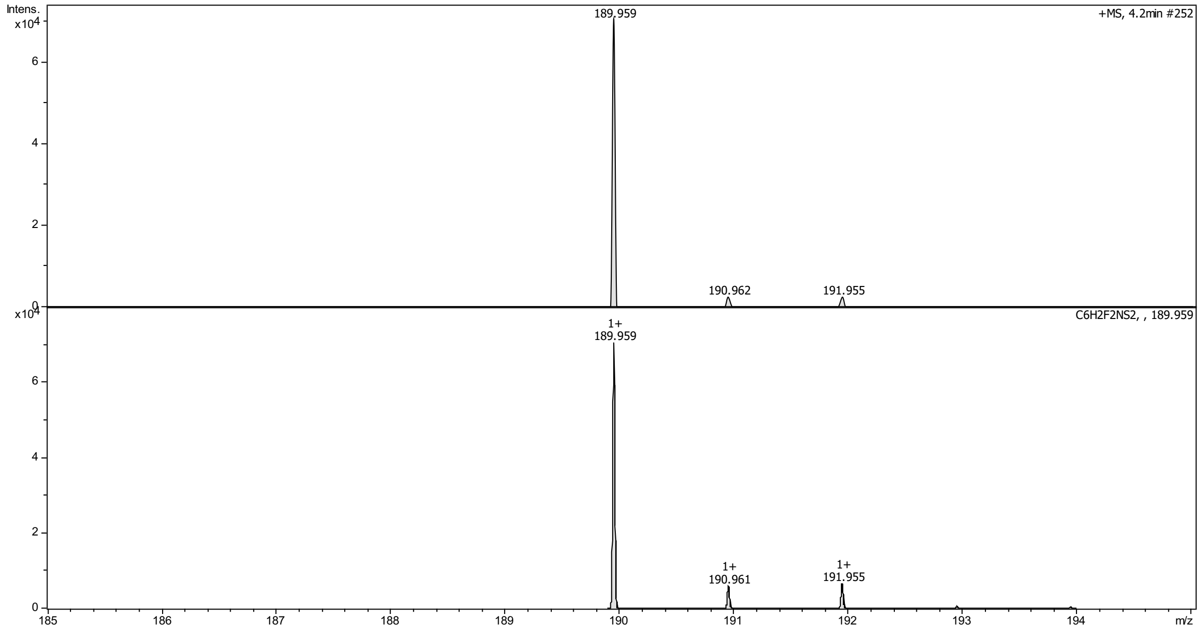

Figure S2. ESI-MS spectrum of **6** measured in the positive-ion mode. (a) 150-700 Da range, and (b) 185-195 Da range with experiment (above) and simulation (below).

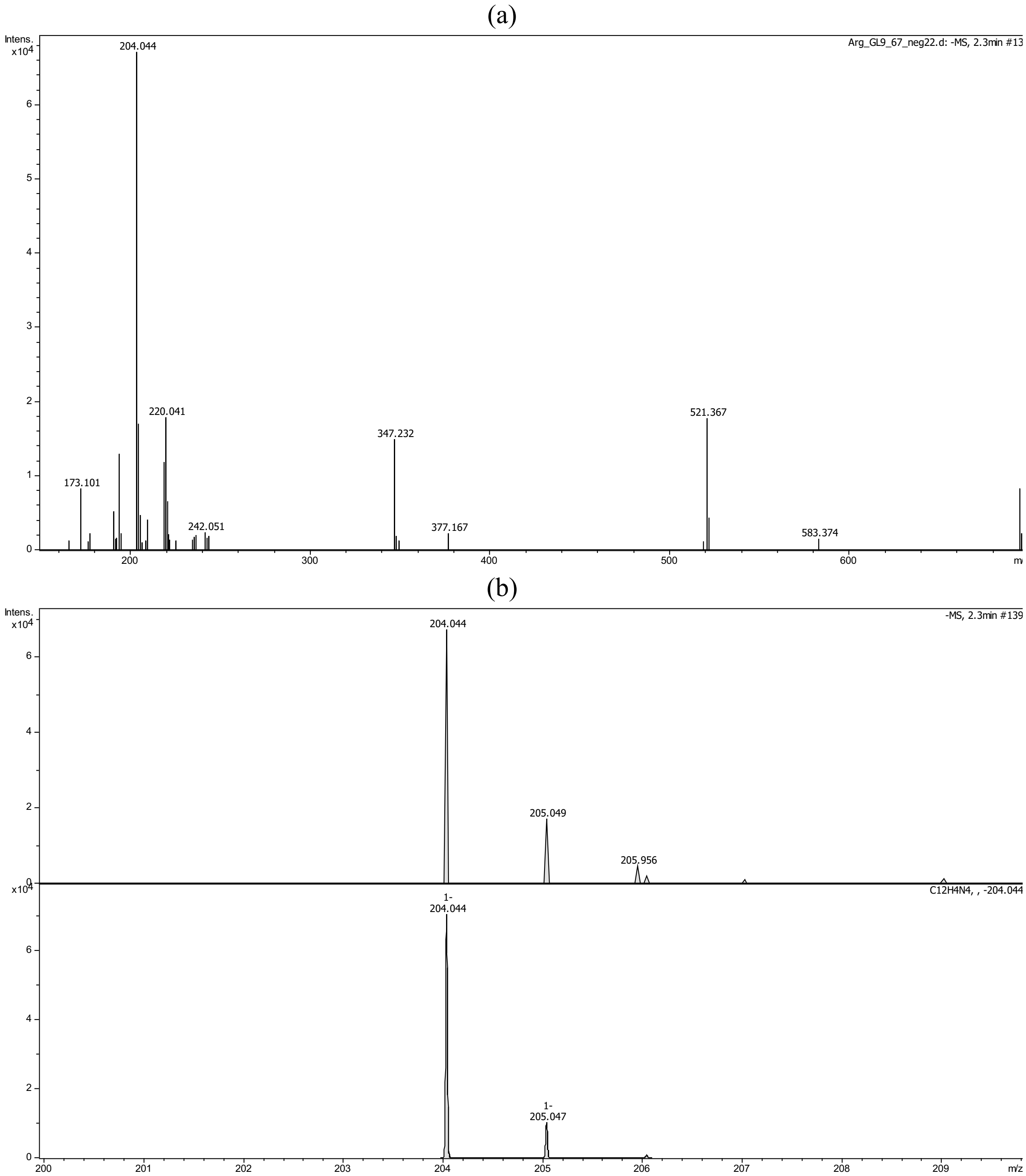

Figure S3. ESI-MS spectrum of **6** measured in the negative-ion mode. (a) 150-700 Da range, and (b) 200-210 Da range with experiment (above) and simulation (below).

Adduct **7** ($C_{24}H_4F_8N_6S_4$, formula mass 655.925 Da). A sample was prepared directly before measurements as solution in CH_3CN with concentration ~2.7 mg mL^{-1}. Ions **7**$^+$, [**7**-Na]$^+$ or [**7**-K]$^+$, and **7**$^-$ were not observed. Instead, intense peaks with m/z = 225.941 corresponding with the error lesser than 0.001 Da (4.4 ppm) to **3**$^+$ ($C_6F_4NS_2$), and m/z = 204.044 with the error lesser than 0.001

Da (4.9 ppm) corresponding to TCNQ˙⁻ ($C_{12}H_4N_4$), respectively, were detected, both exhibiting correct isotopic distributions (Figures S4 and S5).

(a)

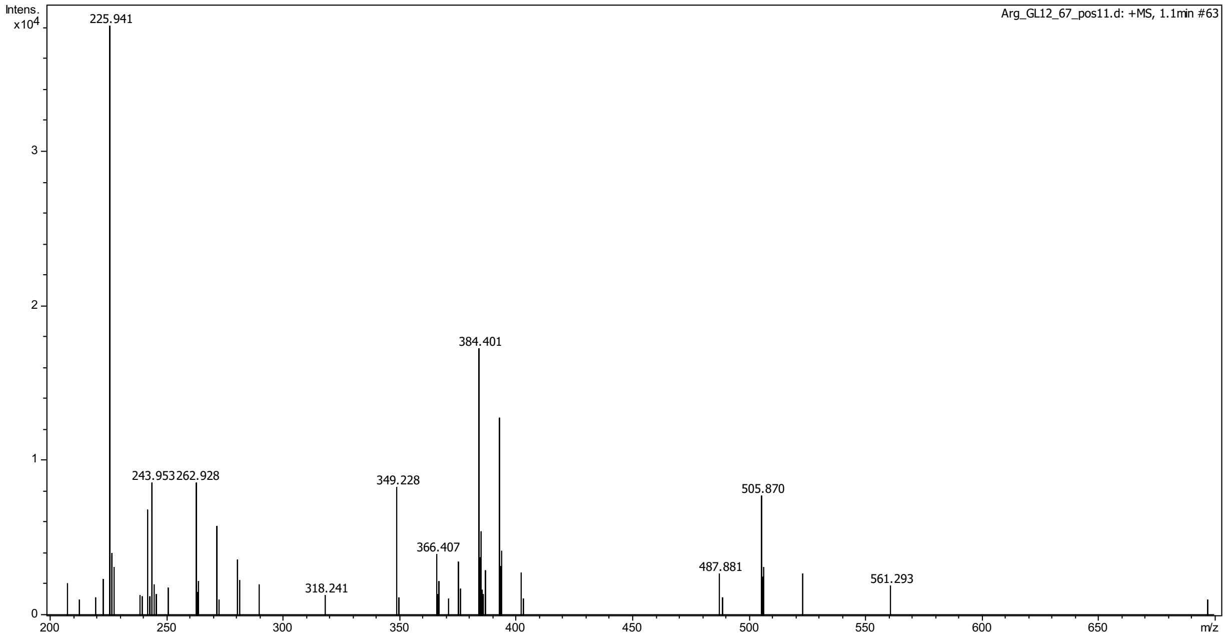

(b)

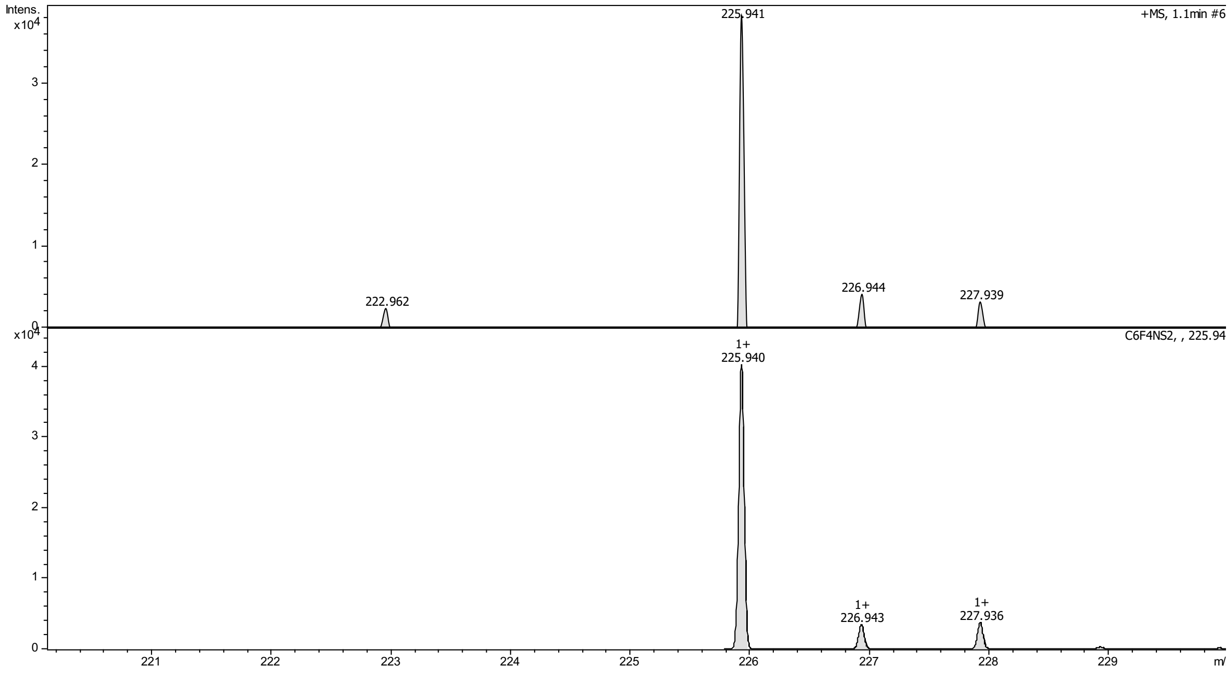

Figure S4. ESI-MS spectrum of **7** measured in the positive-ion mode. (a) 200-700 Da range, and (b) 220-230 Da range with experiment (above) and simulation (below).

(a)

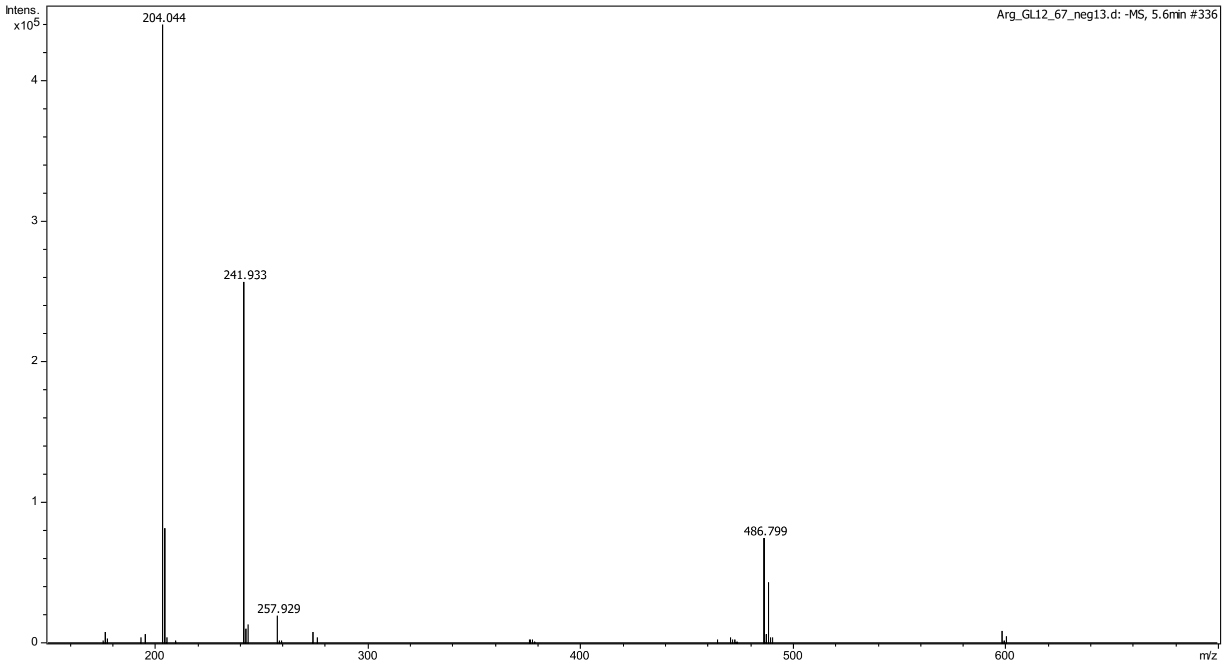

(b)

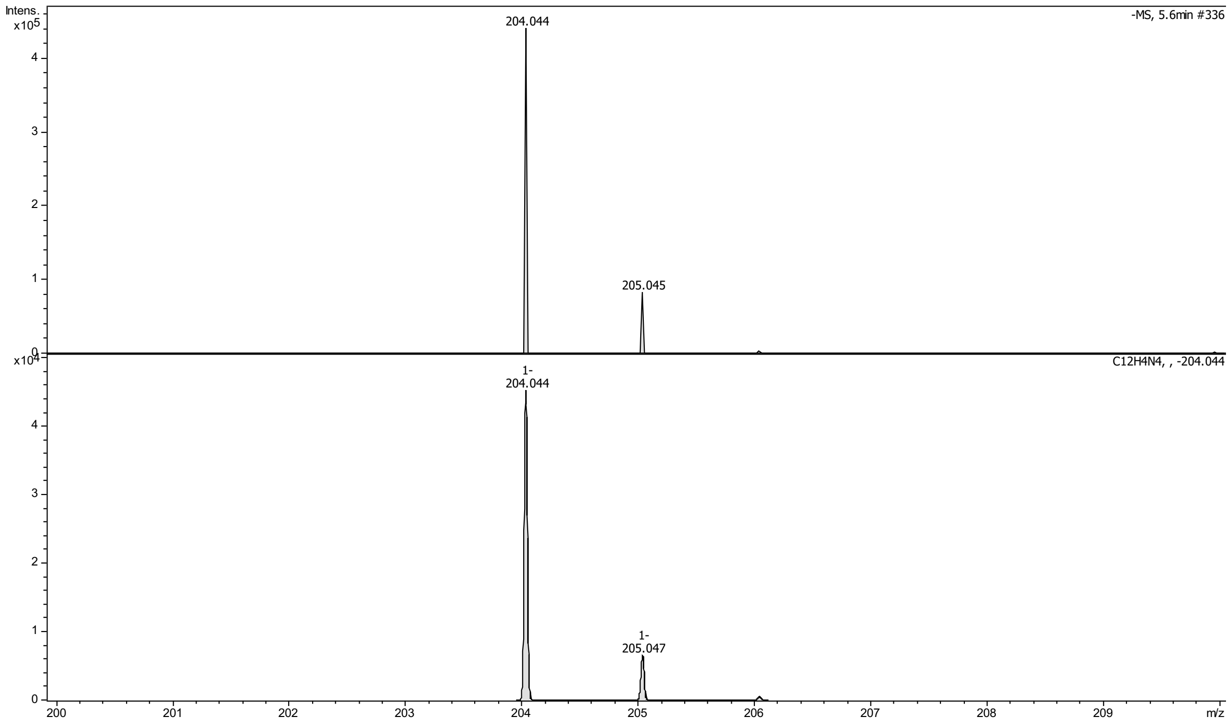

Figure S5. ESI-MS spectrum of **7** measured in negative-ion mode. (a) 150-700 Da range, and (b) 200-210 Da range with experiment (above) and simulation (below).

S4. NUCLEAR MAGNETIC RESONANCE (NMR) SPECTROSCOPY

^{19}F NMR spectra (282.4 MHz) of **3**$^{+}$Cl^{-} and **4**$^{+}$Cl^{-} are shown in Figure S6.

(a) **3**$^{+}$Cl$^{–}$

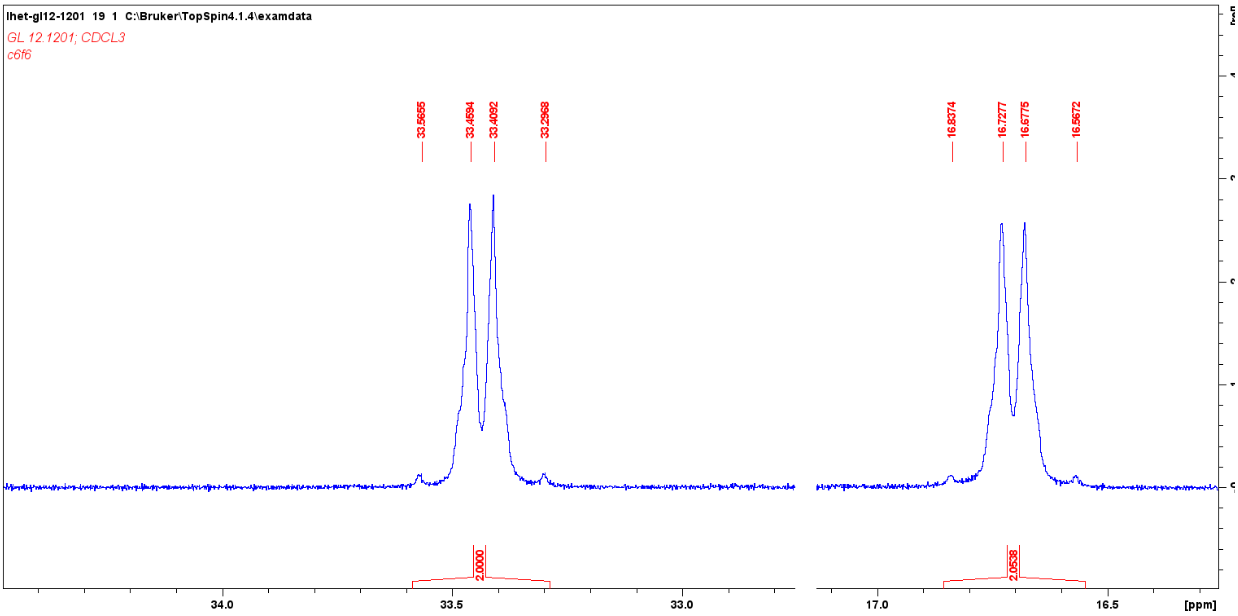

(b) **4**$^{+}$Cl$^{–}$ i

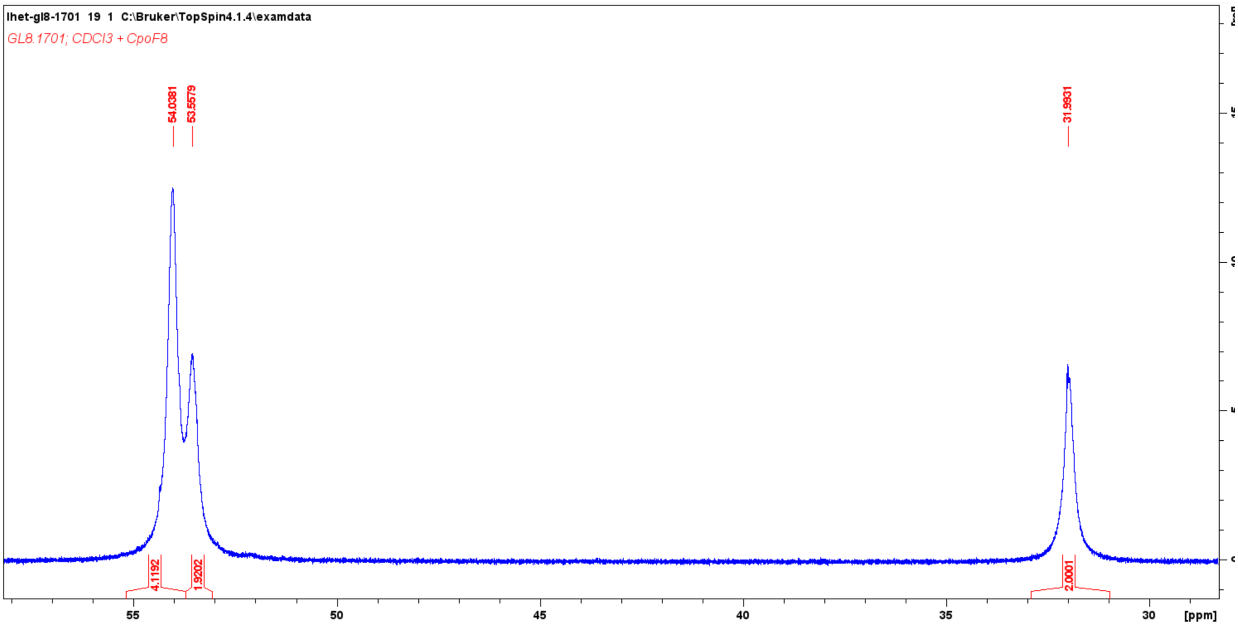

Figure S6. ^{19}F NMR spectrum of (a) **3**$^{+}$Cl$^{–}$ and (b) **4**$^{+}$Cl$^{–}$ in $CDCl_3$.

^{1}H and ^{19}F NMR spectra of **9**, **10**, and **11** in solution are shown in Figures S7, S8 and S9, respectively.

(a) ^{1}H

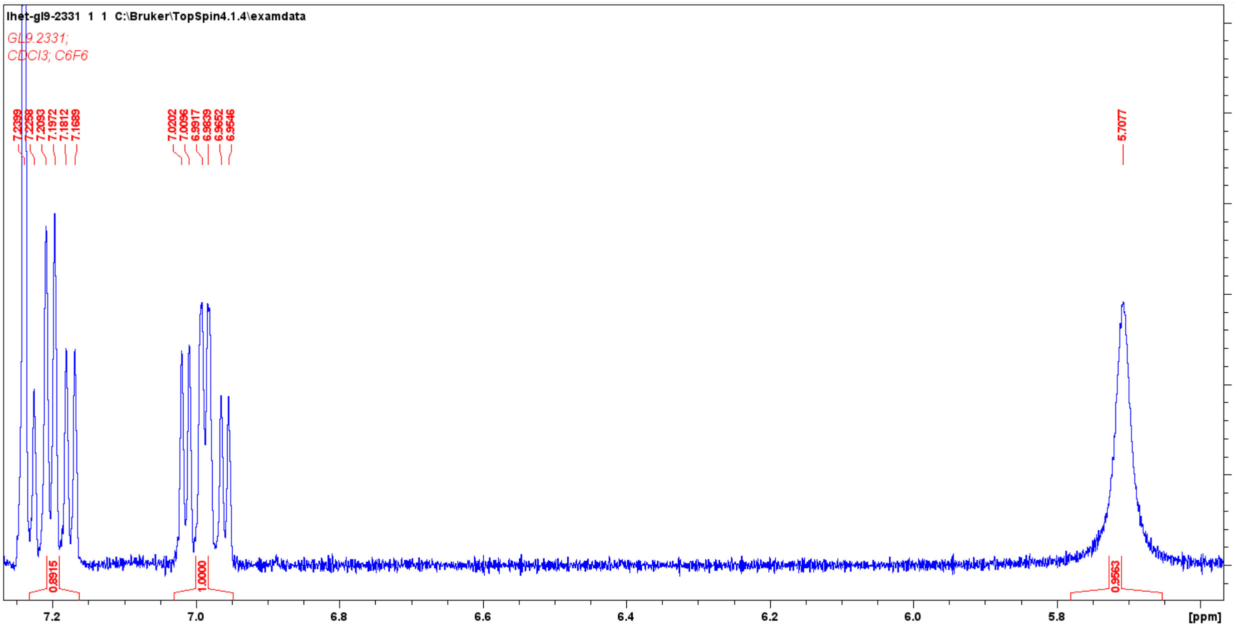

(b) ^{19}F

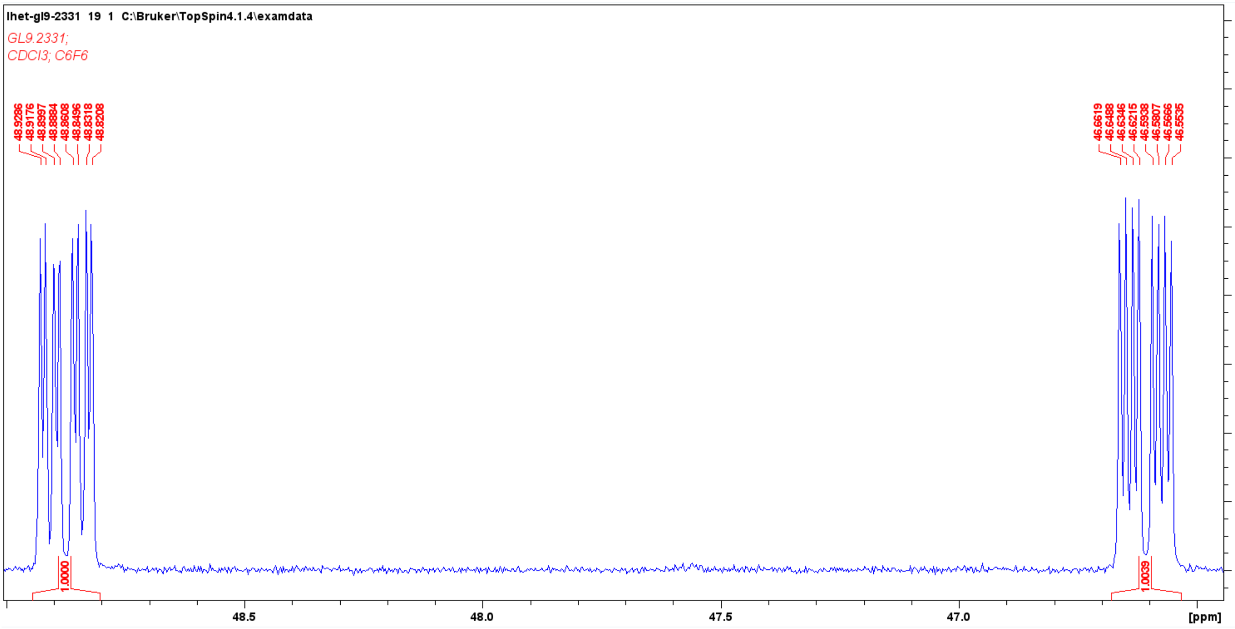

Figure S7. (a) ^{1}H and (b) ^{19}F NMR spectra of **9** in $CDCl_3$.

(a) ^{1}H

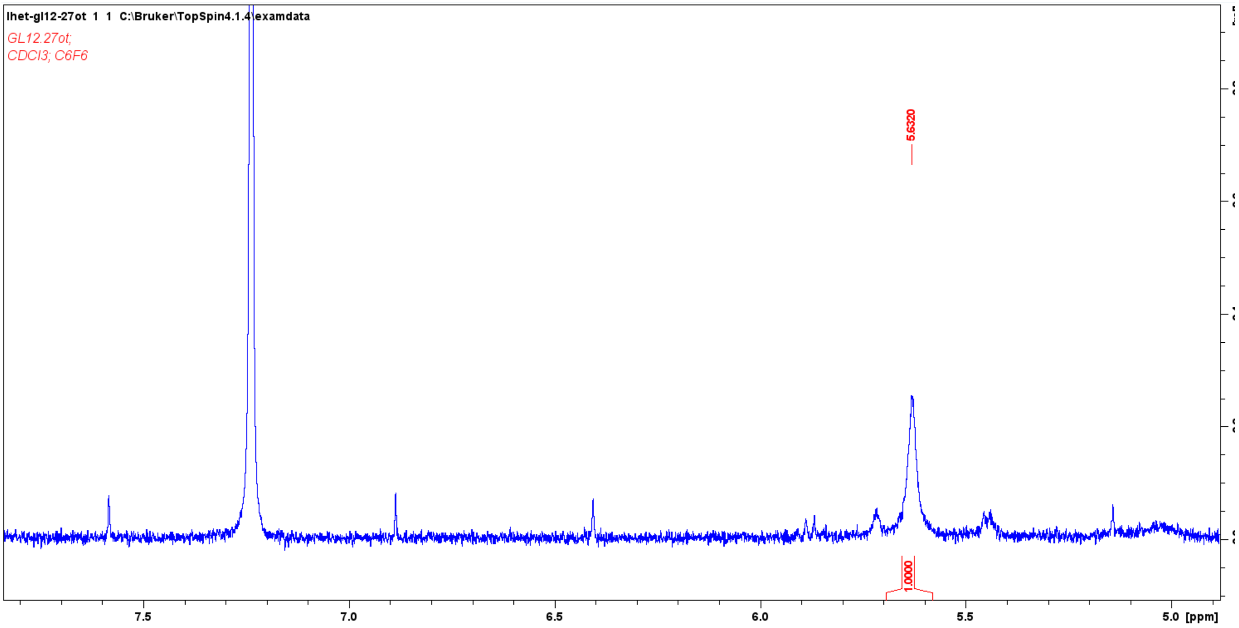

(b) ^{19}F

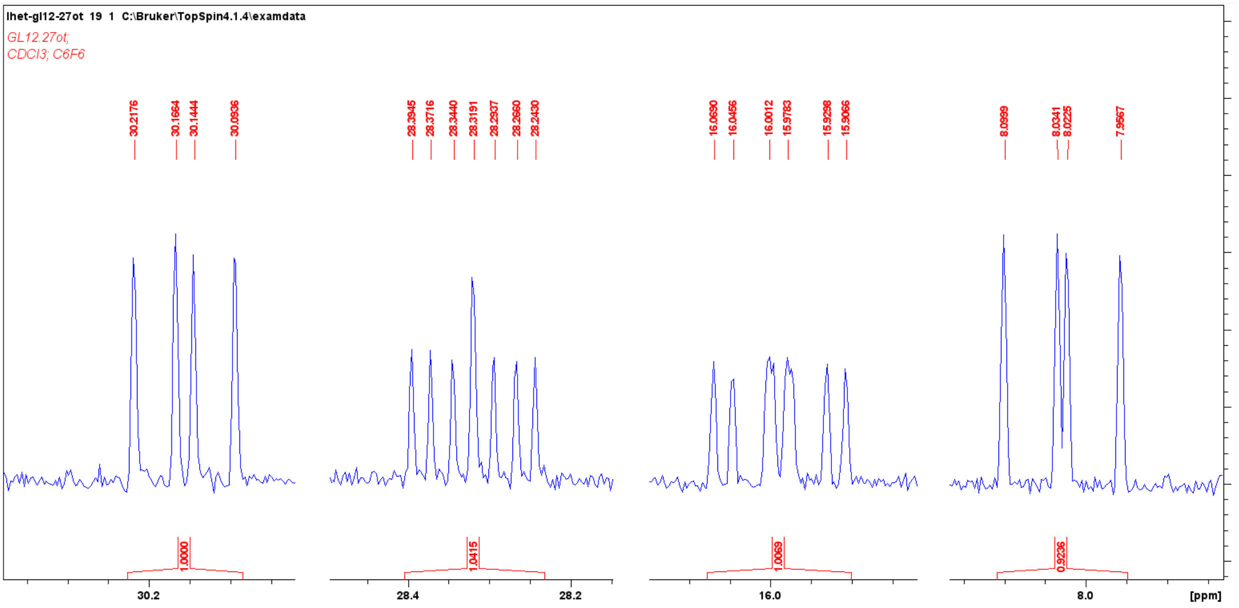

Figure S8. (a) ^{1}H and (b) ^{19}F NMR spectra of **10** in $CDCl_3$.

(a) ^{1}H

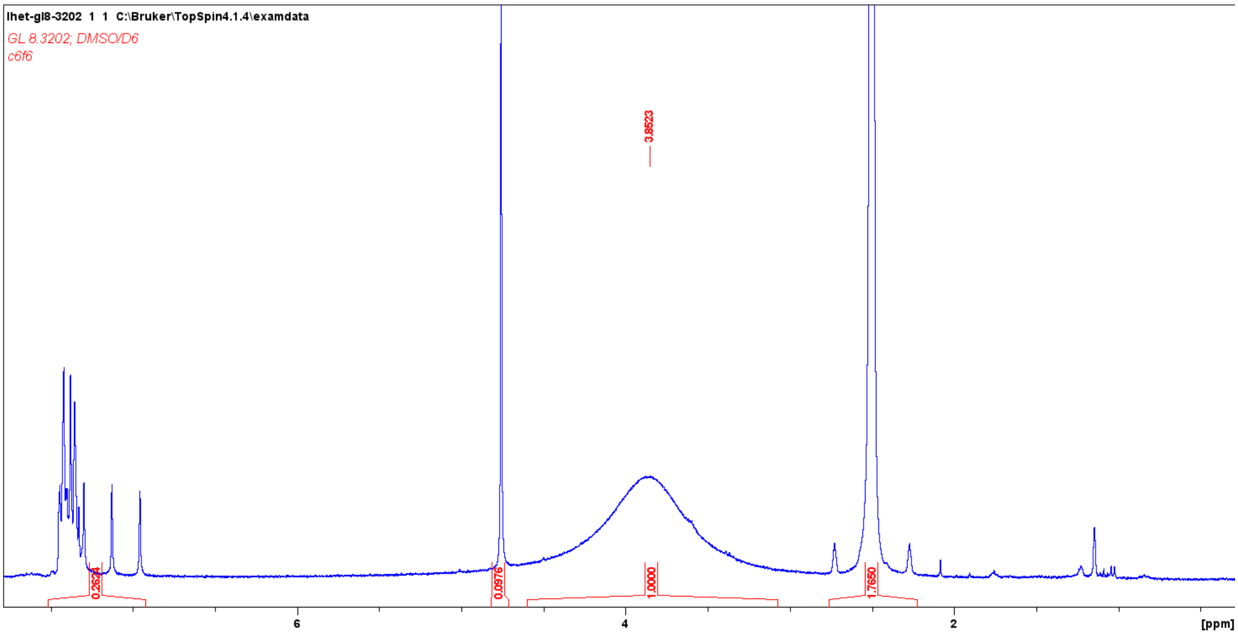

(b) ^{19}F

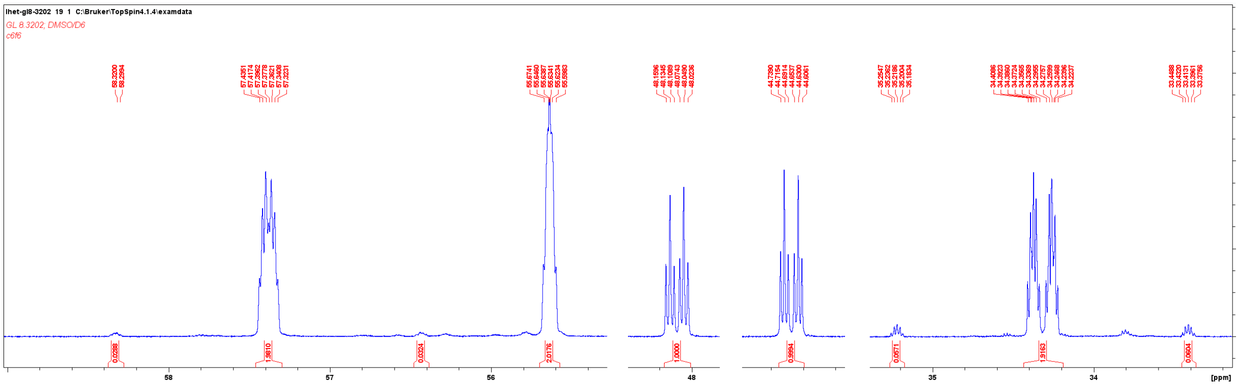

Figure S9. (a) ^{1}H and (b) ^{19}F NMR spectra of **11** in $(D_3C)_2S{=}O$.

^{1}H and ^{19}F NMR spectra of $\mathbf{2}^{+}CF_3CO_2^{-}$ in CF_3CO_2H and $\mathbf{2}^{+}Cl^{-}$ in $CDCl_3$ / CF_3CO_2H are shown in Figures S10 and S11, respectively.

(a) $\mathbf{2}^{+}CF_3CO_2^{-}$

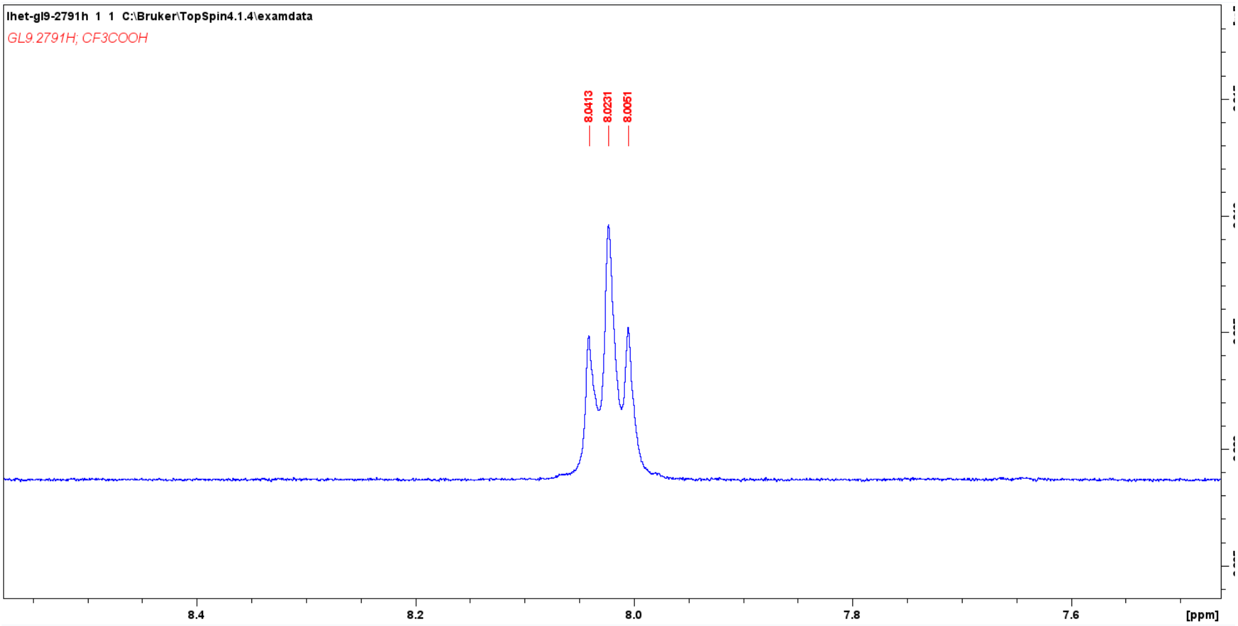

(b) $\mathbf{2}^{+}Cl^{-}$

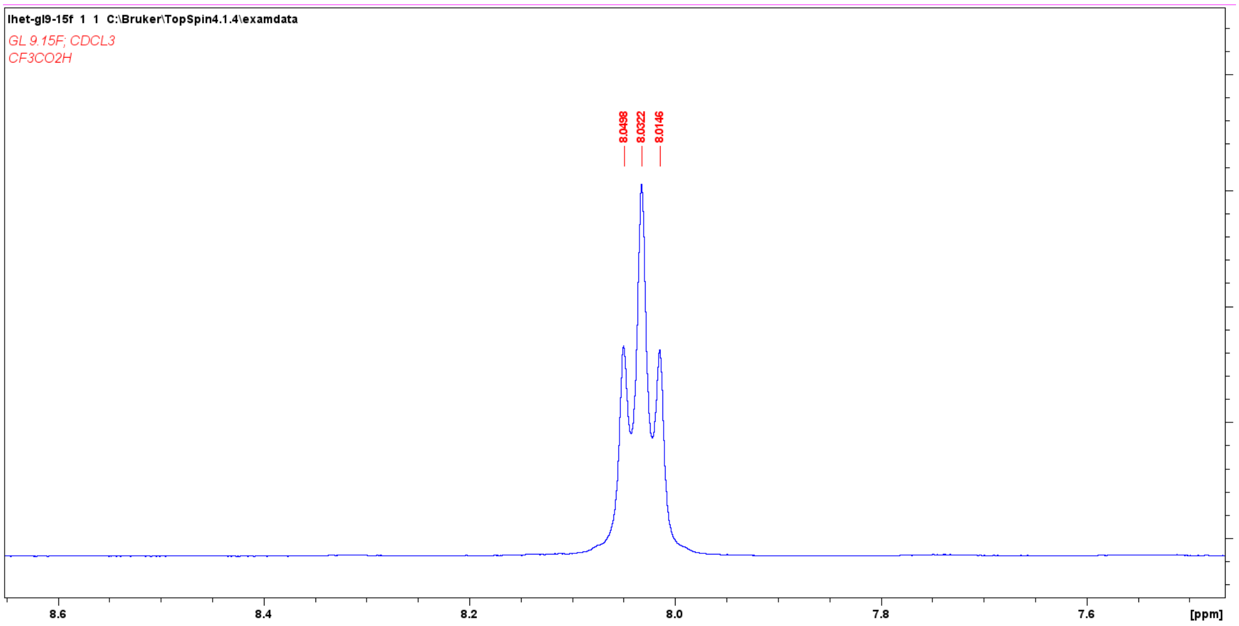

Figure S10. ^{1}H NMR spectra of (a) $\mathbf{2}^{+}CF_3CO_2^{-}$ in CF_3CO_2H and (b) $\mathbf{2}^{+}Cl^{-}$ in $CDCl_3$ / CF_3CO_2H.

(a) **2**$^+CF_3CO_2^-$

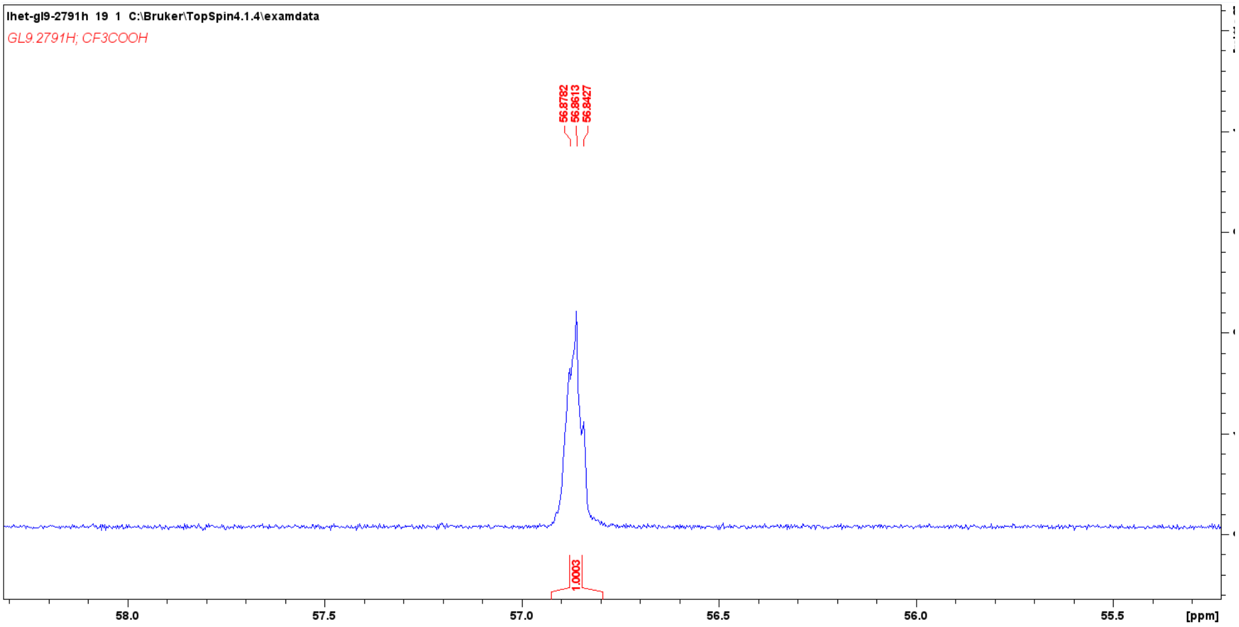

(b) **2**$^+Cl^-$

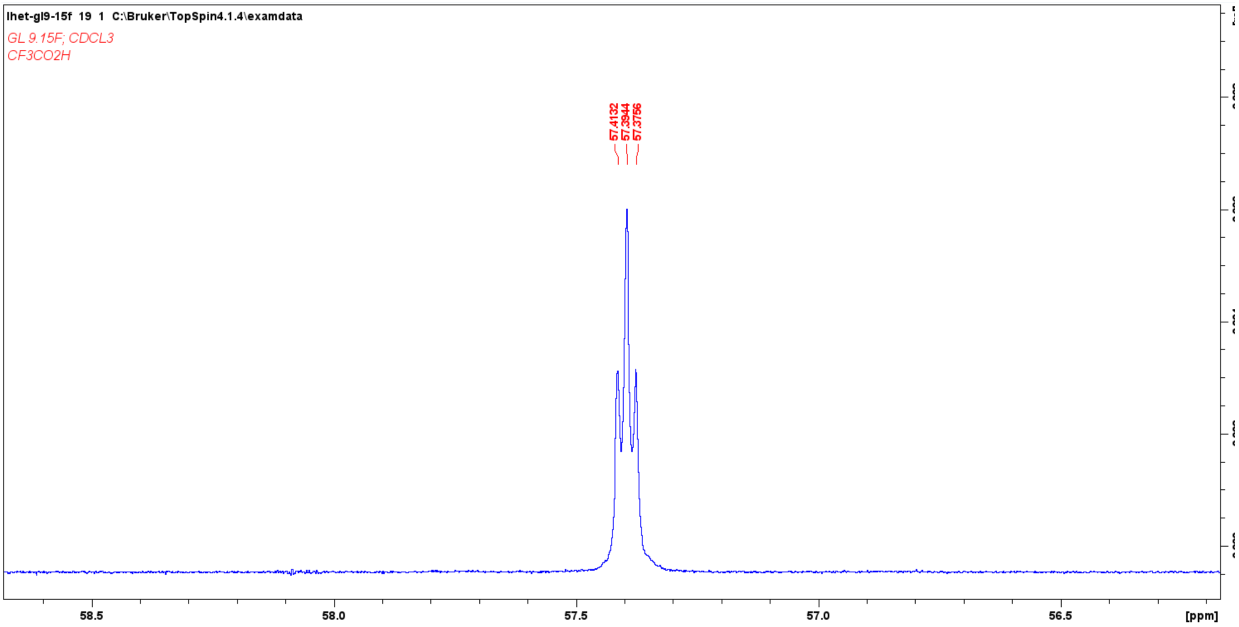

Figure S11. ^{19}F NMR spectra of (a) **2**$^+CF_3CO_2^-$ in CF_3CO_2H and (b) **2**$^+Cl^-$ in $CDCl_3$ / CF_3CO_2H.

Figures S12, S13 and S14 show ^{19}F NMR spectra of **3**$^+CF_3CO_2^-$ / **3**$^+Cl^-$, **4**$^+CF_3CO_2^-$ / **4**$^+Cl^-$, and 3,4,5,6-tetrafuorobenzene-1,2-bis(sulfenyl chloride), respectively.

(a) **3**$^+CF_3CO_2^-$

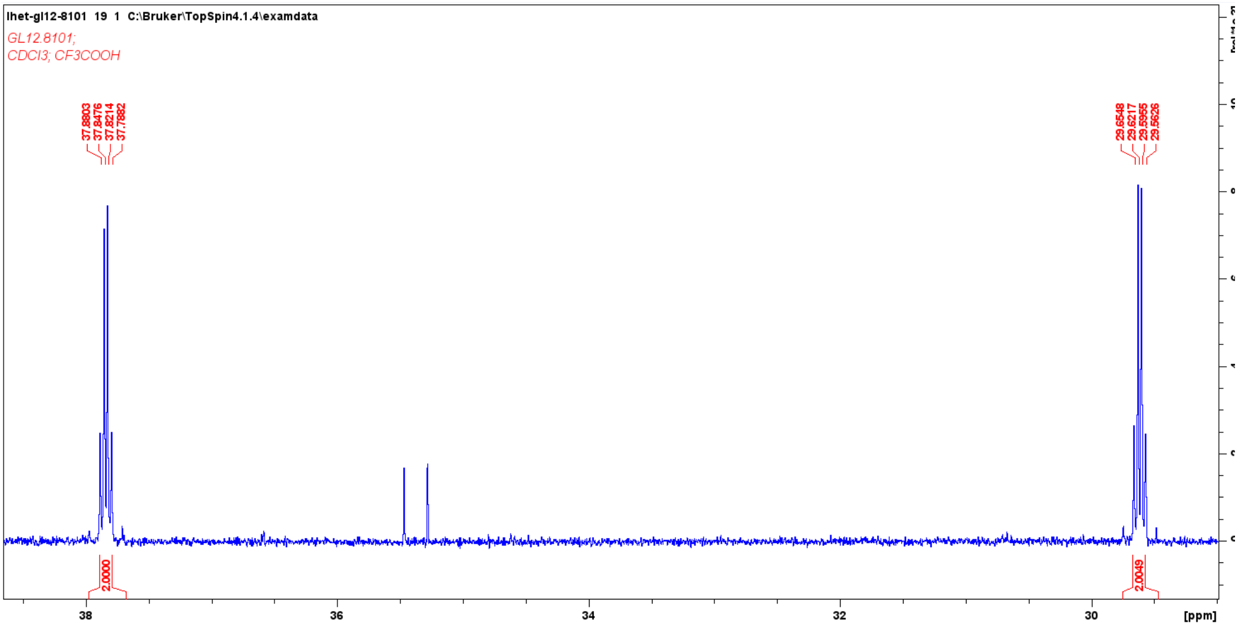

(b) **3**$^+Cl^-$

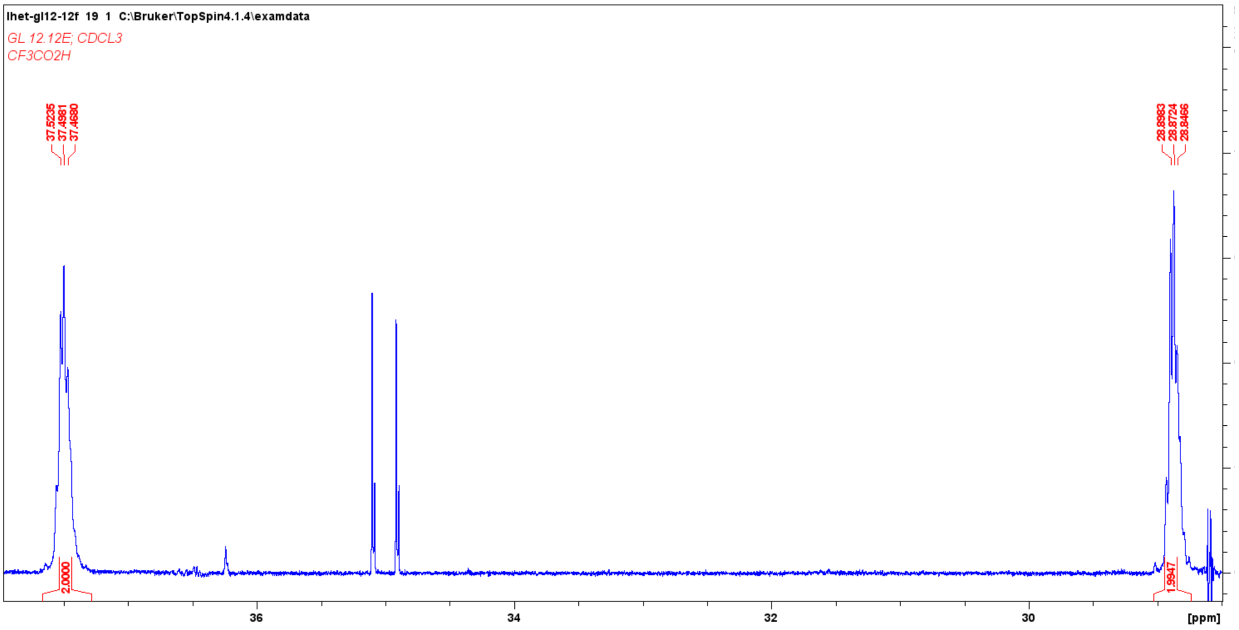

Figure S12. ^{19}F NMR spectra of (a) **3**$^+CF_3CO_2^-$ in CF_3CO_2H, and (b) **3**$^+Cl^-$ in $CDCl_3$ / CF_3CO_2H; doublet at ~35 ppm belongs to CHF_2CO_2H admixture in CF_3CO_2H.

(a) **4**$^{+}CF_3CO_2^{-}$

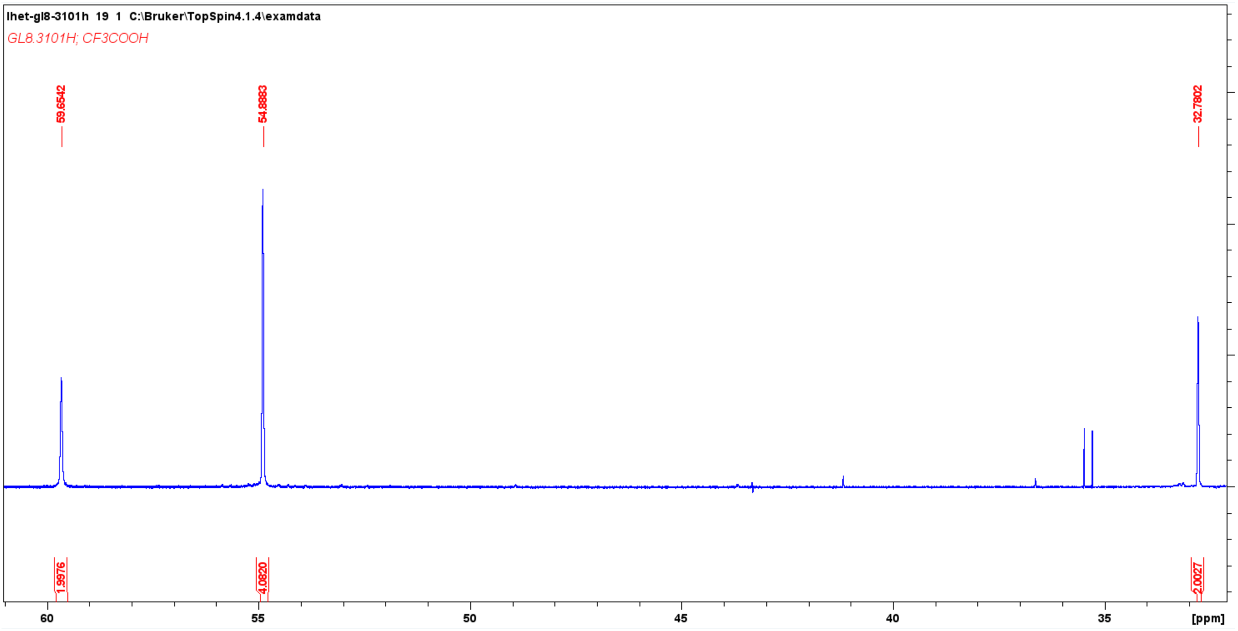

(b) **4**$^{+}Cl^{-}$

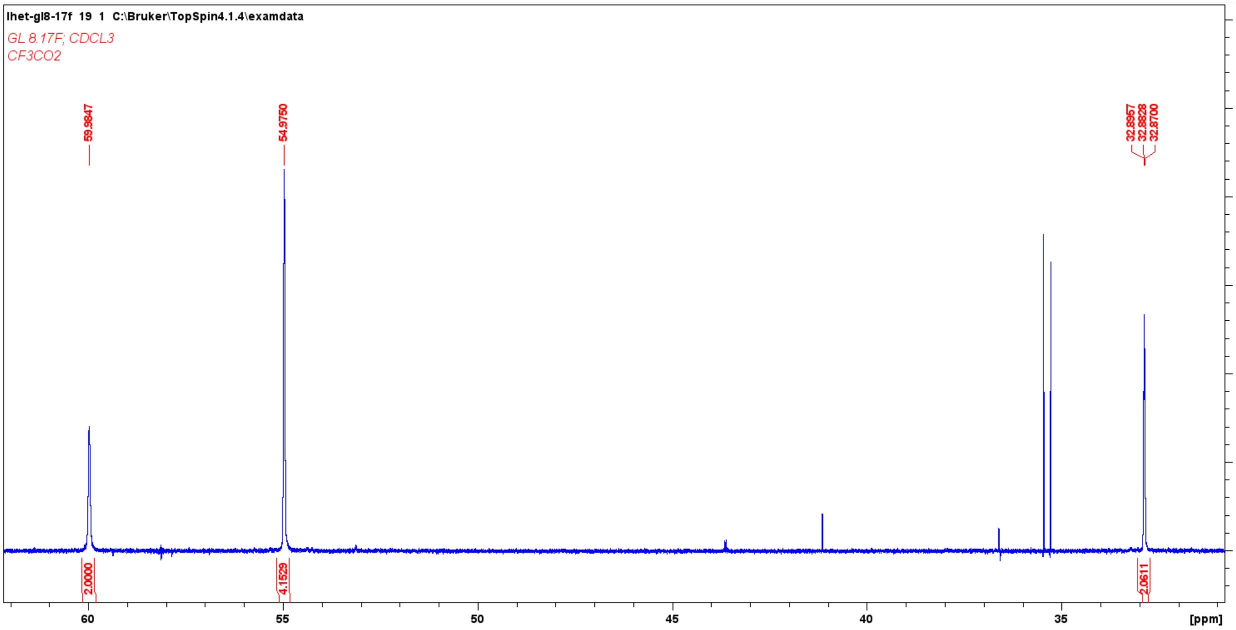

Figure S13. ^{19}F NMR spectra of (a) **4**$^{+}CF_3CO_2^{-}$ in CF_3CO_2H, and (b) **4**$^{+}Cl^{-}$ in $CDCl_3$ / CF_3CO_2H; doublet at ~35 ppm belongs to CHF_2CO_2H admixture in CF_3CO_2H.

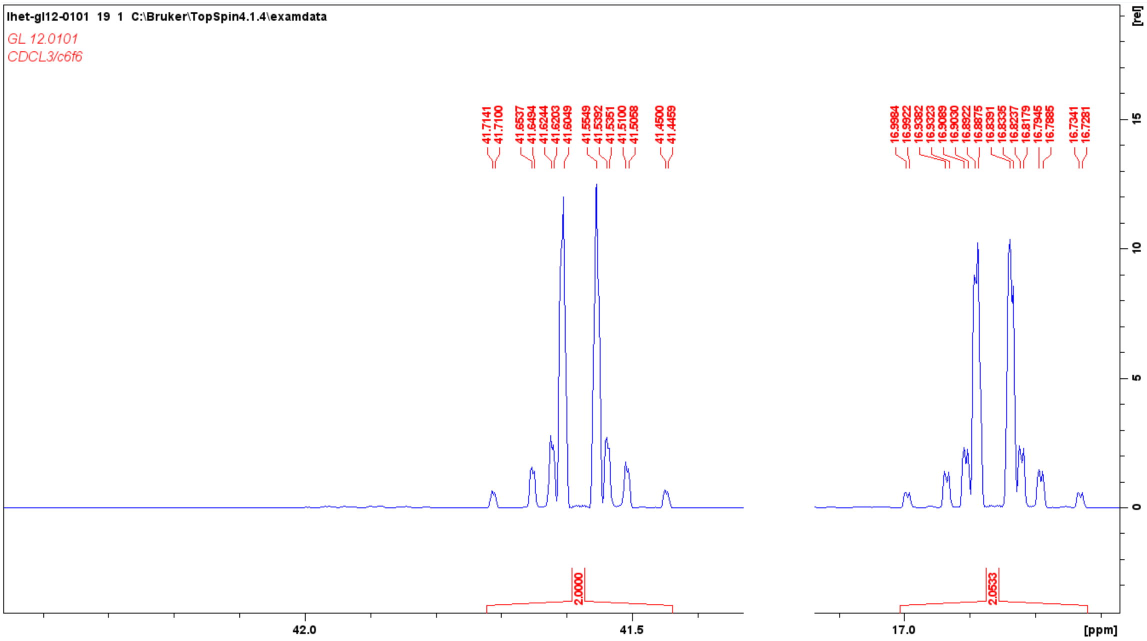

Figure S14. ^{19}F NMR spectrum of 3,4,5,6-tetrafuorobenzene-1,2-bis(sulfenyl chloride) in $CDCl_3$.

S5. INFRARED (IR) and ATTENUATED TOTAL REFLECTANCE (ATR) SPECTROSCOPY

Measured infrared (IR) and attenuated total reflectance (ATR) spectra were obtained for **3**$^{+}$Cl^{-}, **4**$^{+}$Cl^{-}, **3**$^{\cdot}$, **4**$^{\cdot}$, **6–8,** TCNQ, **9–11** (see Figures S15 –S18). IR spectra of **3**$^{\cdot}$, **4**$^{\cdot}$, **6–8**, and TCNQ were calculated for fully optimized geometries at the BP86/def2-qzvppd level of theory using scale factors.[S2]

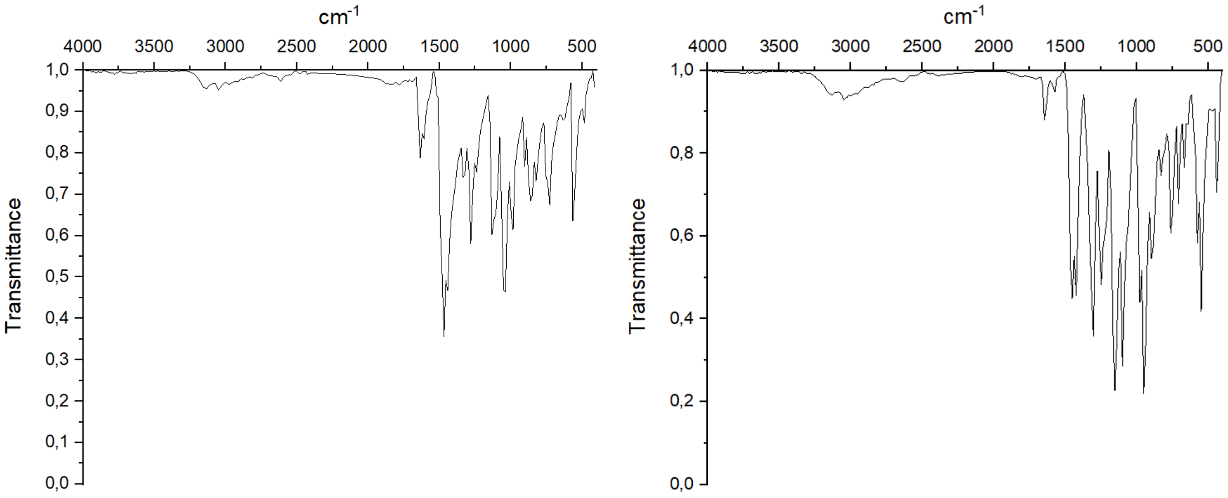

3^{+}Cl^{-} **4**$^{+}$Cl^{-}

Figure S15. Experimental ATR IR spectrum of **3**$^{+}$Cl^{-}. and **4**$^{+}$Cl^{-}.

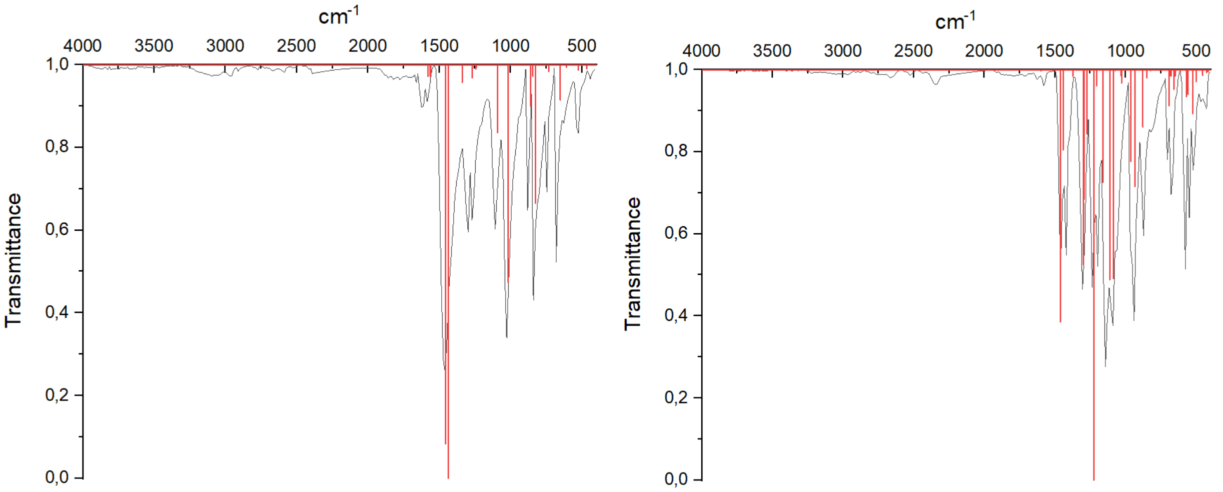

3$^{\cdot}$ **4**$^{\cdot}$

Figure S16. Experimental ATR IR (black line) and BP86/def2-qzvppd-calculated (red bars: positions and relative intensities of transitions) spectra of **3**$^{\cdot}$ (scaling factor 1.05) and **4**$^{\cdot}$ (scaling factor 1.0).

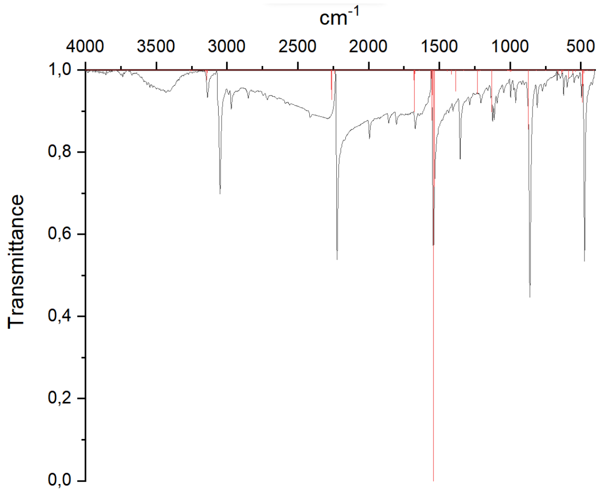

TCNQ

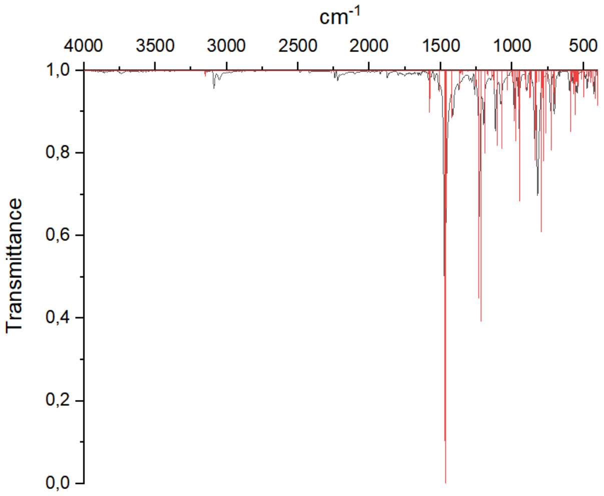

6

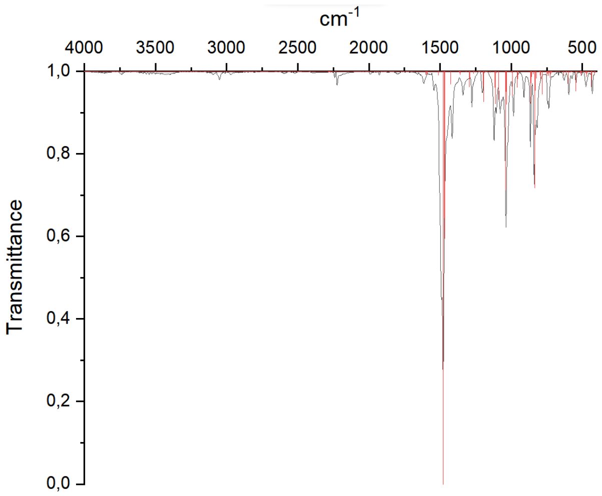

7

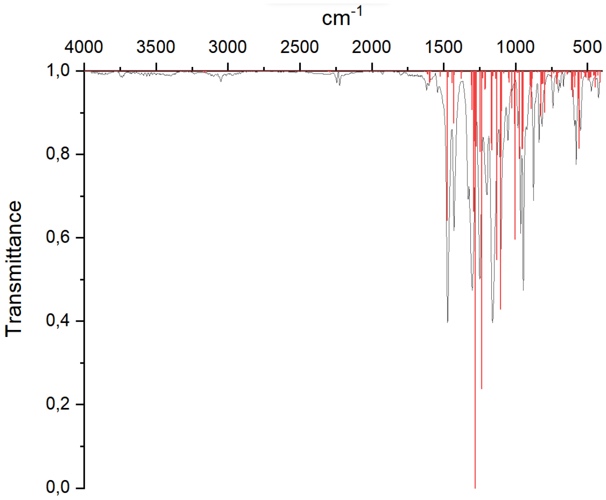

8

Figure S17. Experimental in KBr (black line) and BP86/def2-qzvppd-calculated (red bars: positions and relative intensities of transitions) IR spectrum of TCNQ (scaling factor 1.0) and **6-8** (scaling factor 0.96).

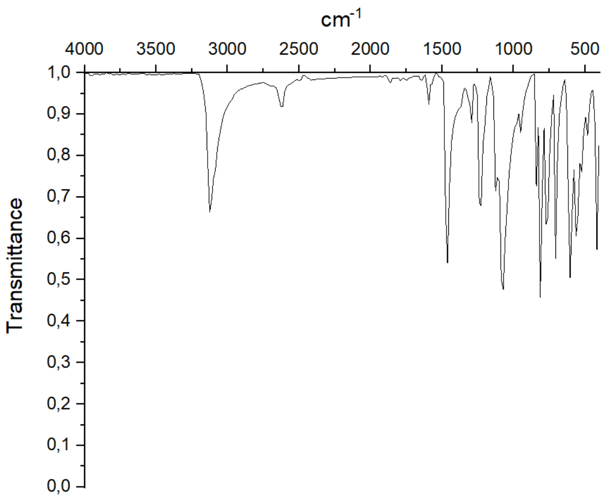

9

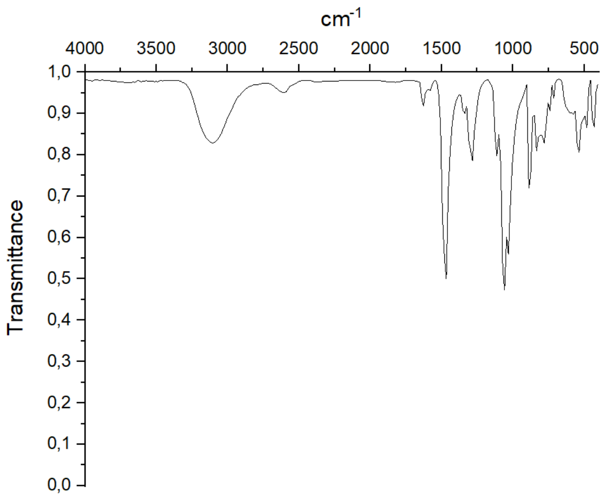

10

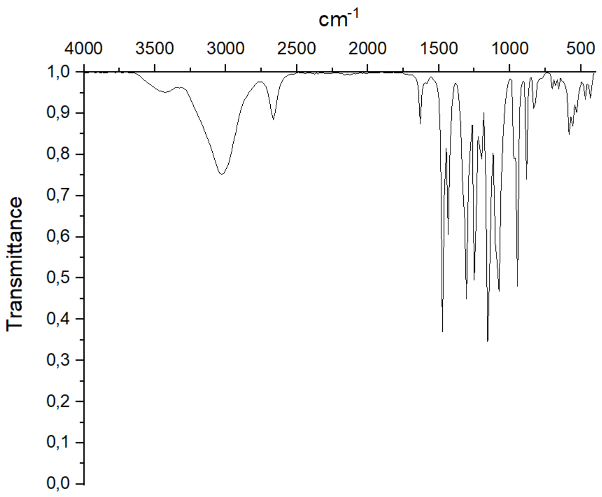

11

Figure S18. Experimental ATR IR spectrum of **9-11**.

S6. ULTRAVIOLET-VISIBLE (UV-Vis) SPECTROSCOPY

Measured UV-Vis spectra of **3**$^{+}$Cl^{-}, **4**$^{+}$Cl^{-}, **2**$^{\cdot}$, **3**$^{\cdot}$, **4**$^{\cdot}$, and **6–11** is shown in Figures S19 – S21. For **3**$^{\cdot}$ and **4**$^{\cdot}$, UV-Vis spectra were calculated for fully optimized geometries by TD-DFT[S3] at the (U)B3LYP/def2-qzvppd level of theory. Solvent CH_2Cl_2 was accounted with conductor-like polarizable continuum model (CPCM).[S4]

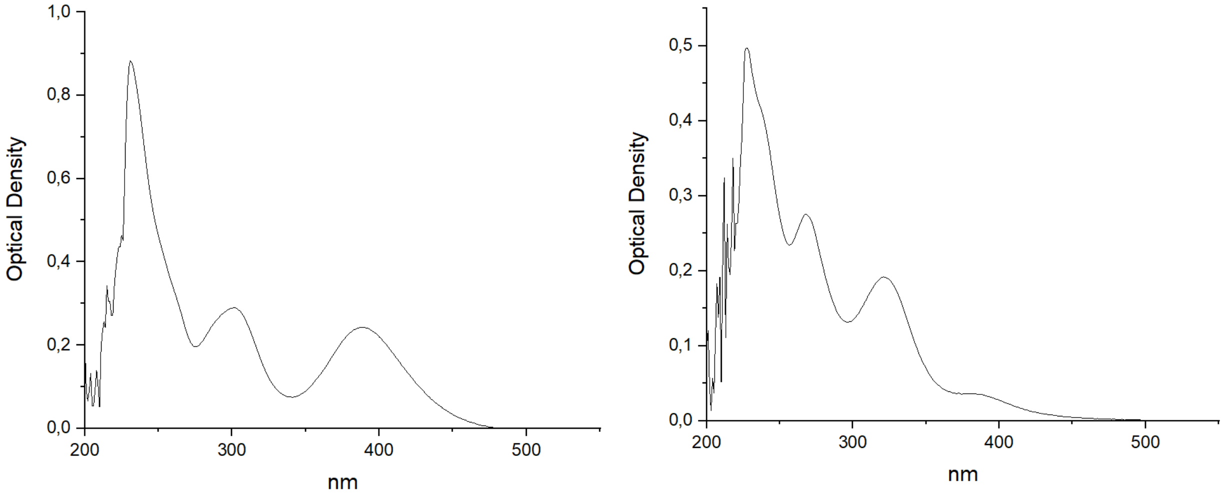

3^{+}Cl^{-} **4**$^{+}$Cl^{-}

Figure S19. UV-Vis spectra of **3**$^{+}$Cl^{-} and **4**$^{+}$Cl^{-} in CH_2Cl_2 solution.

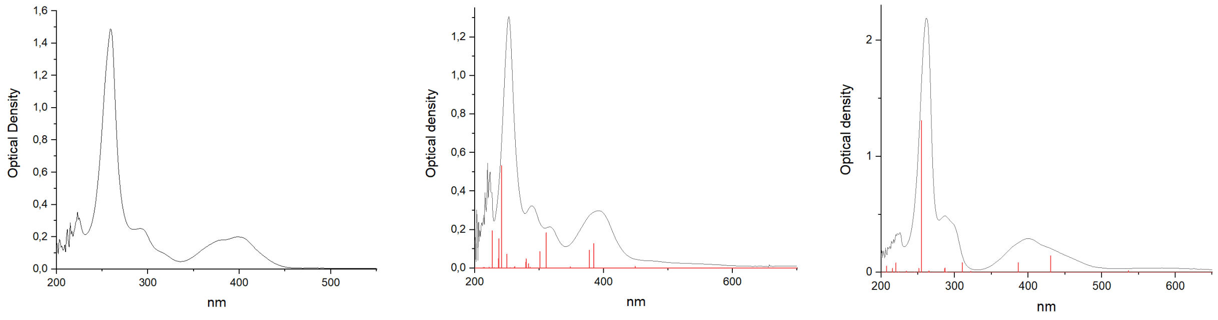

2$^{\cdot}$ **3**$^{\cdot}$ **4**$^{\cdot}$

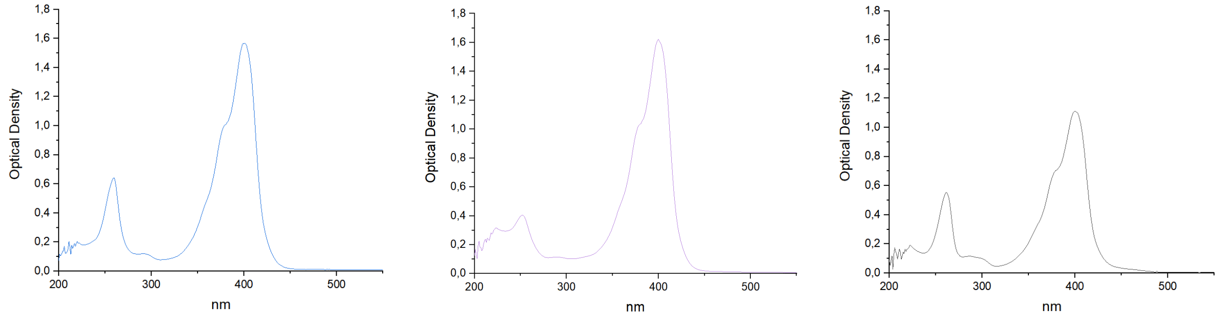

6 **7** **8**

Figure S20. Experimental UV-Vis spectra (black line) of **2**$^{\cdot}$, **3**$^{\cdot}$, **4**$^{\cdot}$, and **6–8** in CH_2Cl_2 solution. Calculated positions and relative intensities of electronic transitions (red bars) at TD-(U)B3LYP/def2-qzvppd-level of **3**$^{\cdot}$ and **4**$^{\cdot}$.

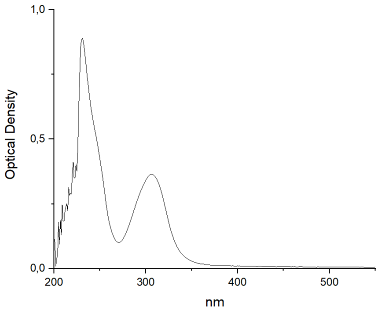

9

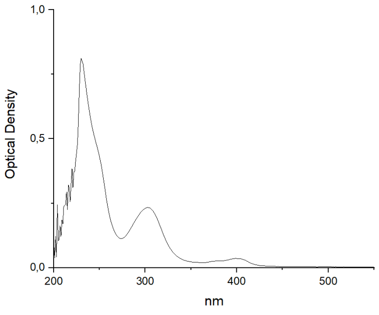

10

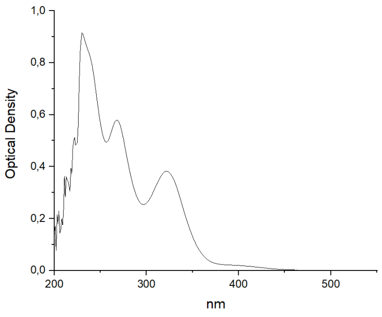

11

Figure S21. UV-Vis spectra of **9-11** in CH_2Cl_2 solution.

S7. SIMULTANEOUS THERMOGRAVIMETRY – DIFFERENTIAL SCANNING CALORIMETRY

Simultaneous thermogravimetry-differential scanning calorimetry (TG-DSC) measurements were performed for **3**$^+$Cl$^-$, **4**$^+$Cl$^-$ (see Figure S22) and **6**–**8** (see Figure S23).

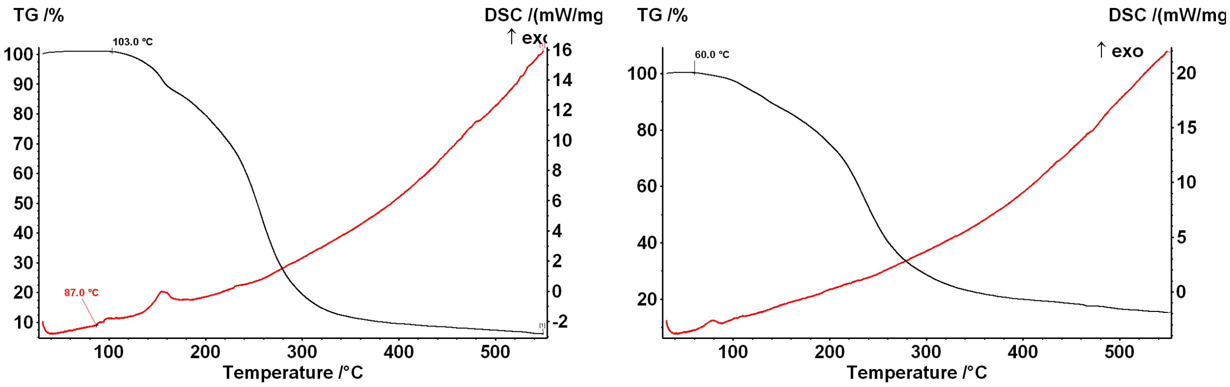

3$^+$Cl$^-$ **4**$^+$Cl$^-$

Figure S40. TG-DSC of **3**$^+$Cl$^-$ and **4**$^+$Cl$^-$ (TG, black; DSC, red).

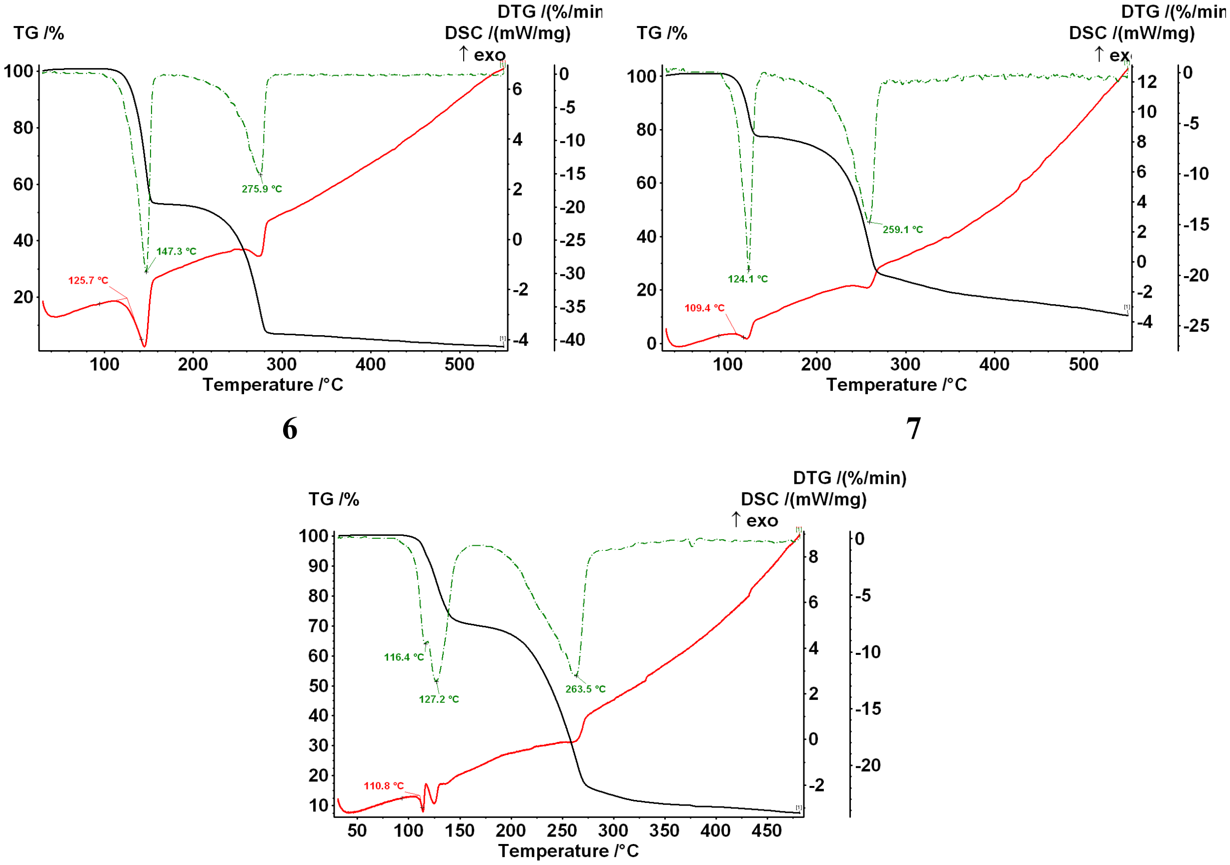

6 **7**

8

Figure S23. Ambient-pressure TG-DSC of **6-8** (TG, black; DTG, green; DSC, red). Under these conditions, **3**˙ and **4**˙ decompose and only **2**˙ sublimes.

S8. ELECTROCHEMISTRY

Experimental peak potentials in CVs are referenced to SCE. The first adiabatic ionization energy ($a\mathrm{EI}_1$) of **1**$^{\cdot}$-**4**$^{\cdot}$ are calculated at the DLPNO-CCSD(T)/aug-cc-pvtz level of theory[S4] as the energy difference of PBE0/aug-cc-pvtz and (U)PBE0/aug-cc-pvtz fully optimized geometries of **R**$^{+}$ and **R**$^{\cdot}$ states, respectively (**R** = **1**-**4**). Solvents CH_3CN and CH_2Cl_2 are accounted with CPCM.[S5] The hfc constants of **3**$^{\cdot}$ and **4**$^{\cdot}$ are calculated at the (U)PBE0 level of theory with DKH-def2-qzvpp and saug-ANO-pvtz basis sets, respectively, using fully optimized geometries.

3^{+}Cl^{-} in CH_3CN

The peak 1C (Figure S24*a*,*d*) is one-electron and reversible, and corresponds to reduction of **3**$^{+}$ into **3**$^{\cdot}$. The linear dependence of 1C current on $v^{0.5}$ indicates the diffusion control of the electrode process (Figure S25). Peak 2C most likely corresponds to reduction of **3**$^{\cdot}$ into **3**$^{-}$. Peaks 2C and 3C of reduction of **3**$^{+}$ (Figure S24*b*,*c*,*e*,*f*) are irreversible for v = 0.1 – 1.5 V s^{-1}. The ratio of peak currents of to that of peak 1C is ~1 and ~2, respectively, at v = 0.1 V s^{-1}. With v = 1.5 V s^{-1}, the ratio decreases to 0.75 and 1.47, respectively (Figure S24*f*). Half-height width of peaks 2C and 3C at 1.5 V s^{-1} is increased to ~0.14 and ~0.19 V, respectively, which is a markedly higher calculated value $|E_p - E_{p/2}|$ = 95.4 mV for irreversible one-electron transfer ($|E_p - E_{p/2}| = 47.7/(n\alpha)$ mV, where n = 1 and α = 0.5). These findings suggest further chemical transformations of reduction products of **3**$^{\cdot}$. Without solution stirring between CV cycles (Figure S26), increased rate of electrode polarization does not increase peak currents of 1C and 2C in the case that the potential sweep covers peak 3C's area. It implies decrease of concentrations of **3**$^{+}$ and **3**$^{\cdot}$ in near-electrode space caused by irreversible chemical transformations.

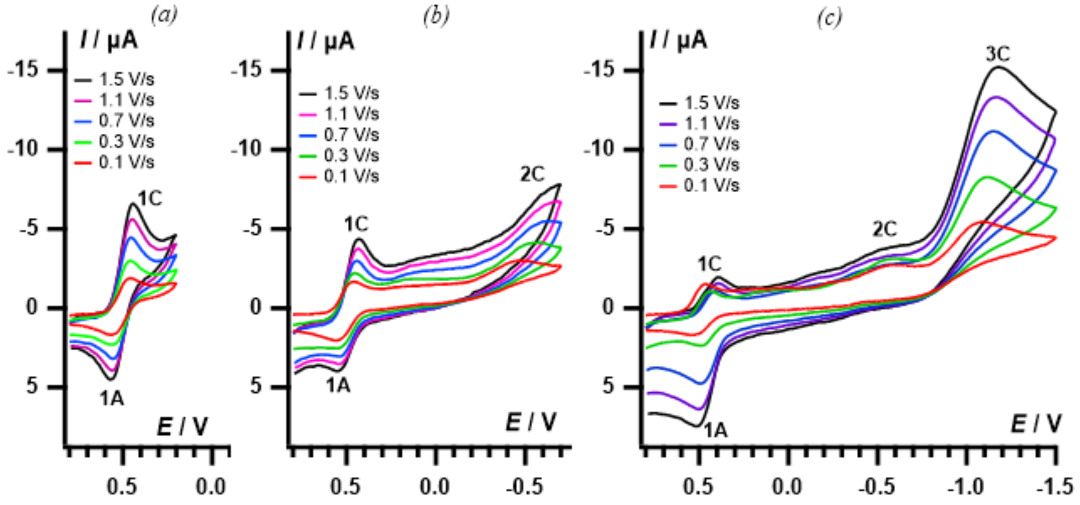

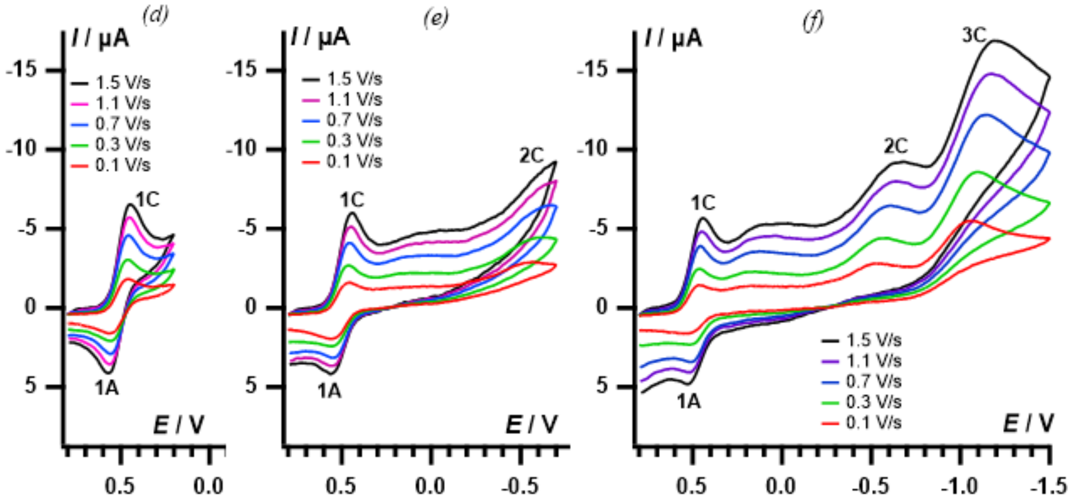

Figure S24. CVs of reduction of $\mathbf{3}^{+}Cl^{-}$ at Pt electrode in CH_3CN with color-indicated potential sweep rates v = 0.1-1.5 V s^{-1}: (*a-c*) without of solution stirring by argon stream between CV cycles, and (*d-f*) with stirring. The potential range (V): (*a, d*) 0.8 > E > 0.2 ; (*b, e*) 0.8 > E > –0.7 ; and (*c, f*) 0.8 > E > –1.5. The peak potentials (V, with respect to SCE; v = 0.1 V s^{-1}): E_p^{1C} = 0.46, E_p^{1A} = 0.53, E_p^{2C} = –0.42, E_p^{3C} = –1.04.

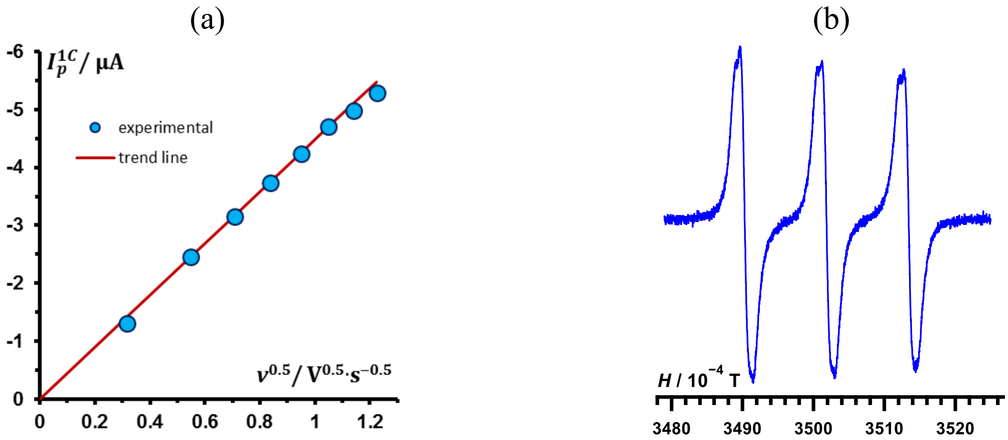

Figure S25. (a) Linear dependence of 1C peak's cathode current upon reduction of $\mathbf{3}^{+}Cl^{-}$ in CH_3CN on $v^{0.5}$. (b) EPR spectrum of $\mathbf{3}^{\cdot}$ obtained by reduction of $\mathbf{3}^{+}Cl^{-}$ at the potential of peak 1C. Hfc constant $a^{14}{}_{N}$ = 11.55 G; hfc constants $a^{19}{}_{F}$ are manifested only by minor splitting at the lines' extremes and not evaluated by modeling due to increased linewidths of 0.6-0.7 G.

CVs of electrochemical oxidation of $\mathbf{3}^{+}Cl^{-}$ in the potential range 0.8-1.8 V exhibit irreversible peaks $1A^{(+)}$, whose height is considerable with that of one-electron reduction peak $1C^{(-)}$, and many-electron peak $2A^{(+)}$.The peak potentials (V, with respect to SCE; v = 0.1 V s^{-1}): $E_p^{1A(+)}$ = 1.12, $E_p^{2A(+)}$ = 1.50 V.

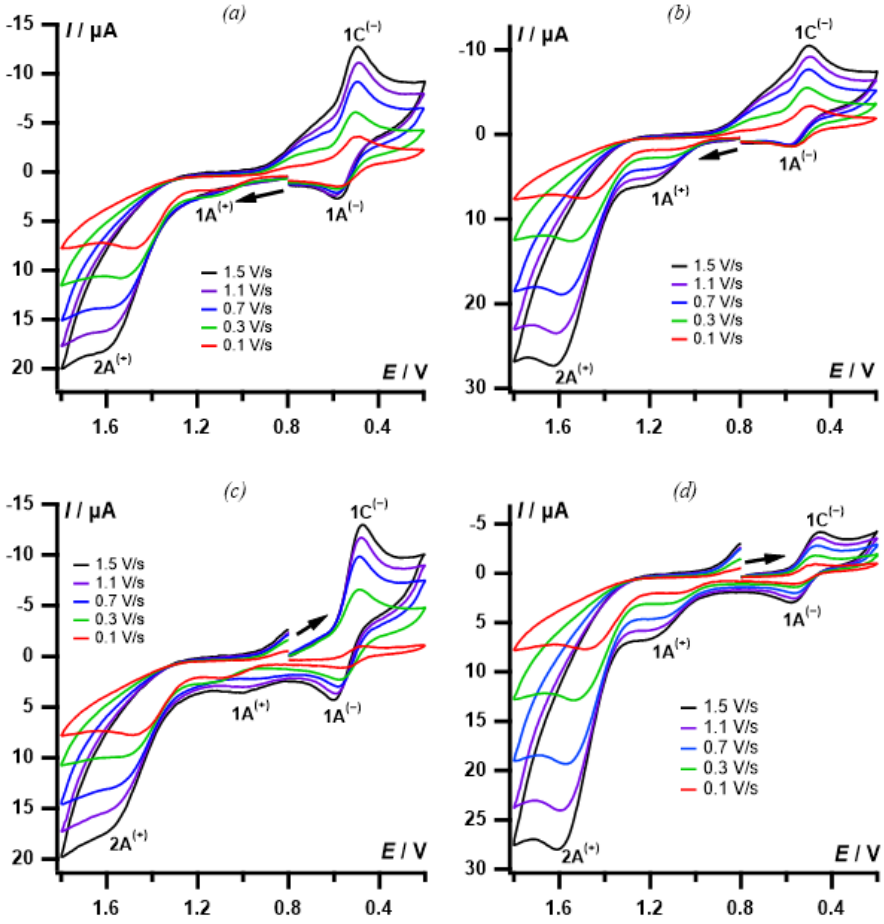

Figure S26. CVs of reduction and oxidation of $\mathbf{3}^{+}Cl^{-}$ at Pt electrode in CH_3CN with color-indicated $v = 0.1 – 1.5$ V s^{-1}: (*a, c*) without solution stirring between CV cycles, and (*b, d*) with stirring; arrow indicates the starting point and direction of potential sweep.

$\mathbf{4}^{+}Cl^{-}$ in CH_3CN

The peak 1C (Figure S27*a,d*) is one-electron and reversible, and corresponds to the reduction of $\mathbf{4}^{+}$ to $\mathbf{4}^{\cdot}$. The linear dependence of the 1C current on $v^{0.5}$ indicates diffusion control of the electrode process (Figure S28).

Peak 2C most likely corresponds to reduction of $\mathbf{4}^{\cdot}$ into $\mathbf{4}^{-}$. Peaks 2C and 3C of reduction of $\mathbf{4}^{+}$ (Figure S27*b*,*c*,*e*,*f*) are irreversible for $v = 0.1 – 2.2$ V s^{-1}. Without solution stirring between CV cycles (Figure S29), increased rate of electrode polarization does not increase peak currents of 1C and 2C in the case that the potential sweep covers peak 3C's area. It implies decrease of concentrations of $\mathbf{4}^{+}$ and $\mathbf{4}^{\cdot}$ in near-electrode space caused by irreversible chemical transformations.

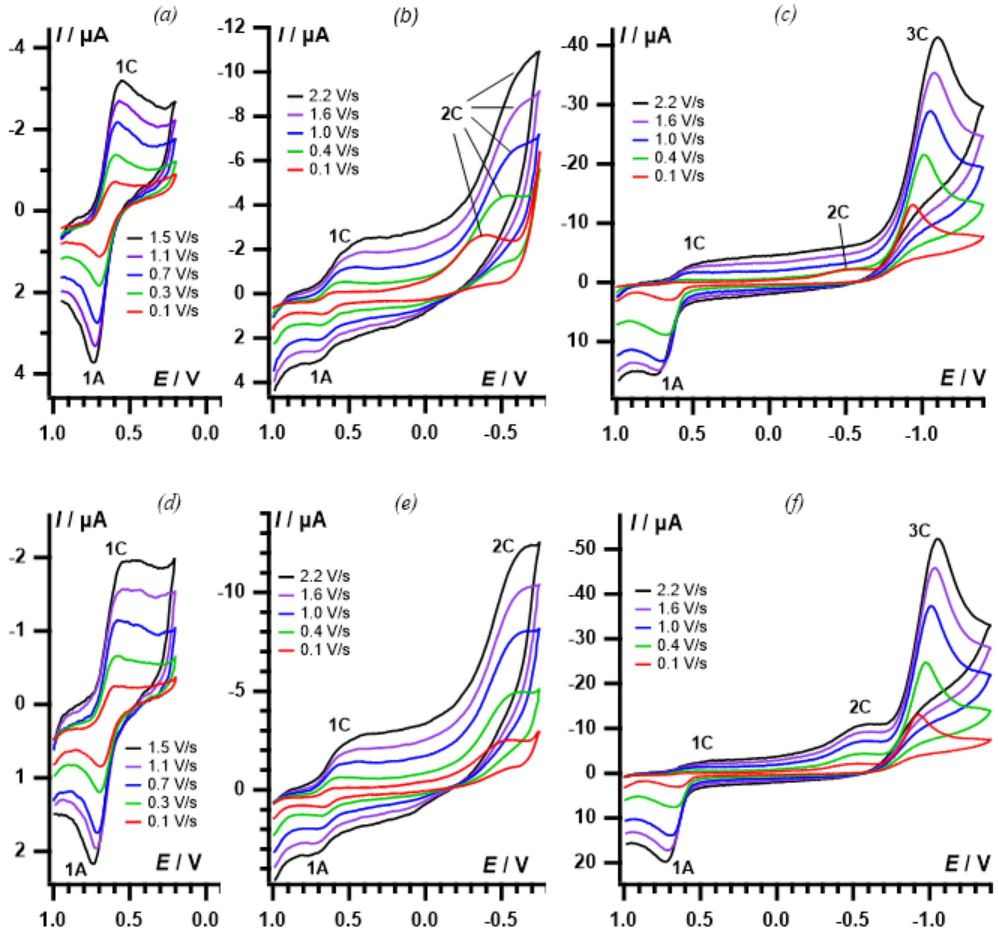

Figure S27. CVs of electrochemical reduction of **4**$^{+}$Cl^{-} at Pt electrode in CH_3CN with color-indicated potential sweep rates v = 0.1 – 2.2 V s^{-1}: (*a-c*) without solution stirring between CV cycles by argon stream, and (*d-f*) with the stirring. The potential range (V): (*a, d*) 1.0 > E > 0.2, (*b, e*) 1.0 > E > –0.8, and (*c, f*) 1.0 > E > –1.4 V. The peak potentials (V, with respect to SCE; v = 0.1 V s^{-1}): E_p^{1C} = 0.60, E_p^{1A} = 0.70, E_p^{2C} = –0.54, E_p^{3C} = –0.94.

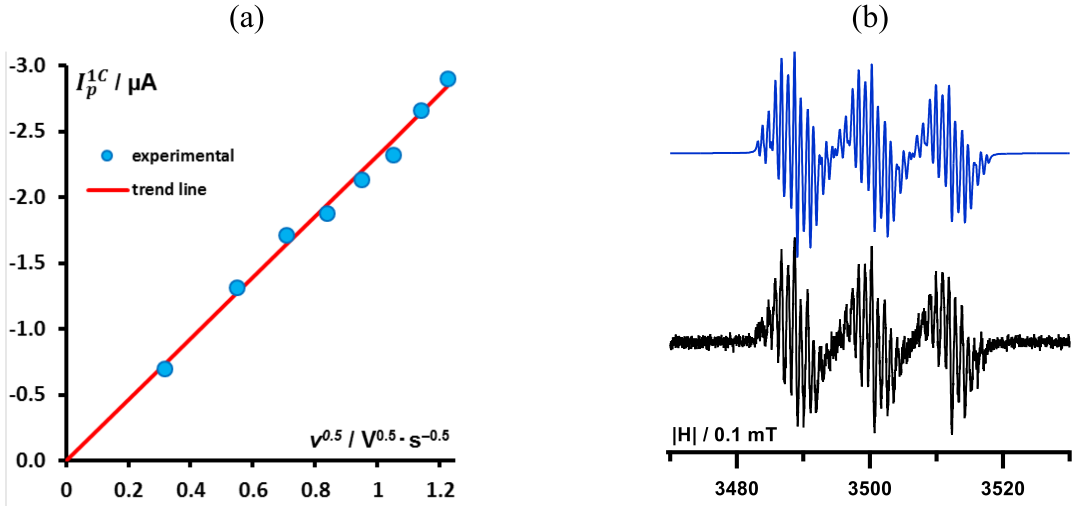

Figure S28. (a) Linear dependence of 1C peak's cathode current upon reduction of **4**$^{+}$Cl^{-} in CH_3CN on $v^{0.5}$. (b) Experimental EPR spectrum of **4**$^{\cdot}$ obtained by reduction of **4**$^{+}$Cl^{-} at the potential of peak 1C (black) and its simulation (blue). Hfc constants (G): $a^{14}{}_{N}$ = 11.60, $a^{19}{}_{F}$ = 2.02 (2F), $a^{19}{}_{F}$ = 1.94 (2F), $a^{19}{}_{F}$ = 0.89 (2F), $a^{19}{}_{F}$ = 0.75 (2F).

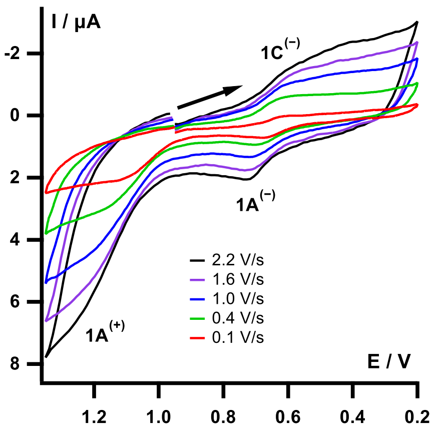

Figure S29. CVs of reduction and oxidation of $\mathbf{4}^{+}Cl^{-}$ at Pt electrode in CH_3CN with color-indicated v = 0.1 – 2.2 V s^{-1} without solution stirring between CV cycles arrow indicates the starting point and direction of potential sweep.

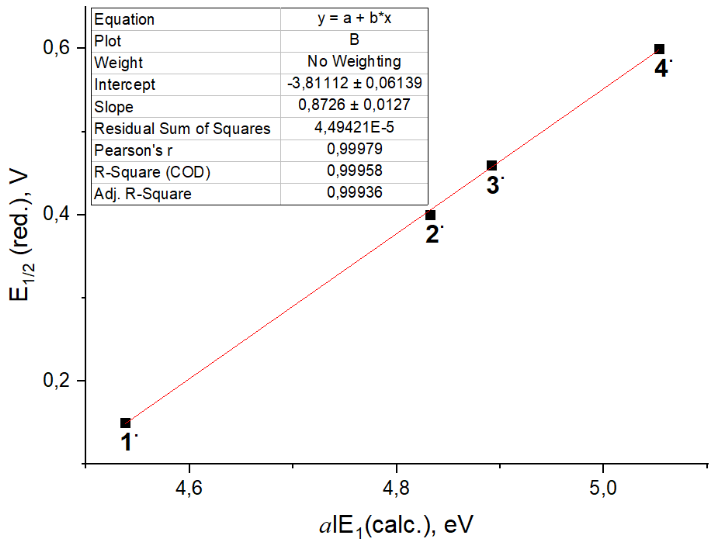

Figure S30. Linear correlation of experimental ECR $E_{1/2}$ potentials of $\mathbf{1}^{+}$-$\mathbf{4}^{+}$ in CH_3CN with calculated aIE$_1$ of **1**˙-**4**˙.

CVs of electrochemical oxidation of $\mathbf{4}^{+}Cl^{-}$ in the potential range 0.8-1.35 V exhibit irreversible peaks $1A^{(+)}$ with $E_p^{1A(+)}$ = 1.13 V; with increased v, the potential shifts in anode area suggesting slower electron transfer.

Correlation of reduction potentials with ionization energies

The $E_{1/2}$ electrochemical reduction potentials of $\mathbf{1}^{+}$-$\mathbf{4}^{+}$ in CH_3CN linearly correlate with aIE$_1$ of **1**˙-**4**˙ calculated at the DLPNO-CCSD(T)/aug-cc-pvtz level of theory, with CPCM accounting for the solvent (Figure S30).

S9. ELECTRON PARAMAGNETIC RESONANCE

Solution

Adducts **6-8** are weakly EPR-active in CH_2Cl_2 solution. Simulated EPR spectra of **6-8** [(U)PBE0 with DKH-def2-qzvpp and saug-ANO-pvtz basis sets for fully optimized geometries] correspond to those of **2**$^{\cdot}$[S6], mixture of **3**$^{\cdot}$ (41%) and **X**$^{\cdot}$ (presumably a product of **3**$^{\cdot}$ mono-defluorination; 59%), and **4**$^{\cdot}$, respectively (see Figure S31 caption for hfc constants in G).

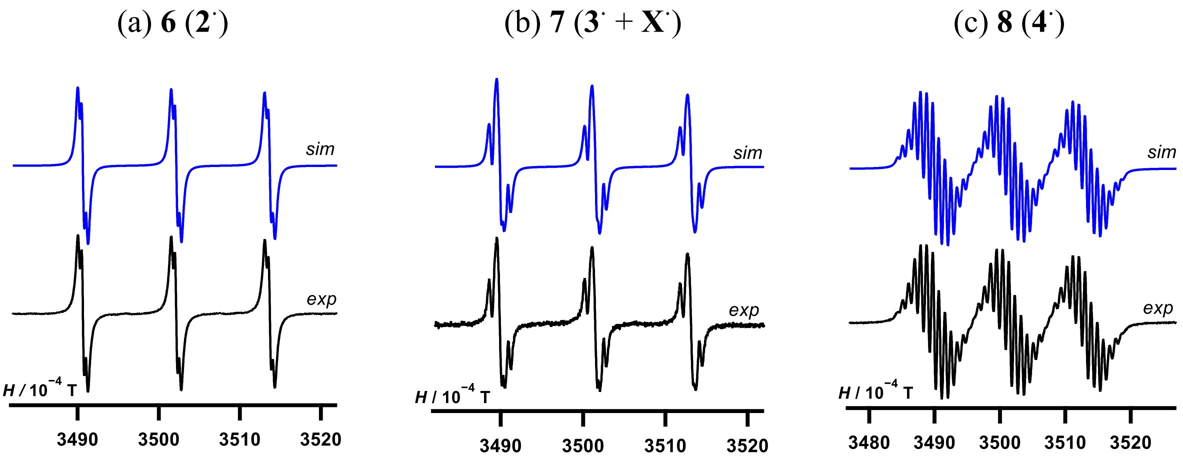

Figure S31. EPR spectra of adducts **6-8** in CH_2Cl_2 solution [hfc constants *a* are given in G] corresponding, respectively, to: (a) **2**$^{\cdot}$ [$a^{14}{}_{N}$ = 11.52, $a^{19}{}_{F}$ = 0.10 (2F), $a^{1}{}_{H}$ = 0.47 (2F)]; (b) **3**$^{\cdot}$ [41%, $a^{14}{}_{N}$ = 11.61, $a^{19}{}_{F}$ = 1.12 (2F), $a^{19}{}_{F}$ = 0.15 (2F)] and **X**$^{\cdot}$ [59%, $a^{14}{}_{N}$ = 11.58; $a^{19}{}_{F}$ = 0.69 (1F), $a^{19}{}_{F}$ = 0.27 (1F), $a^{19}{}_{F}$ = 0.20 (2F)]; and (c) **4**$^{\cdot}$ [$a^{14}{}_{N}$ = 11.60, $a^{19}{}_{F}$ = 2.02 (2F), $a^{19}{}_{F}$ = 1.94 (2F), $a^{19}{}_{F}$ = 0.89 (2F), $a^{19}{}_{F}$ = 0.75 (2F)].

Solid state

According to variable-temperature powder EPR, **3**$^{\cdot}$ is practically silent in the range 195 K < T < 240 K (see Figure S32a,b), except for an anisotropic signal of low intensity, which is less than 0.01% of the intensity of signals above 240 K and can be associated with paramagnetic defects. In the range 240 K < T < 330 K, **3**$^{\cdot}$ exhibits a very rapid increase in the intensity of the EPR signal characterized by an exchange narrowing with increasing temperature suggesting $\mathbf{3}_2$ ↔ 2 **3**$^{\cdot}$ equilibrium of diamagnetic and paramagnetic states (see Figure S32b,c). As can be observed, at 330 K, the intensity reaches its absolute maximum.

In the 260 < T < 315 K range, the temperature dependence of the spin content of **3**$^{\cdot}$ is reversible (see Figure S32c) and the solid-state $\mathbf{3}_2$ ↔ 2 **3**$^{\cdot}$ diamagnetic ↔ paramagnetic equilibrium can be quantitatively described by van't Hoff isobars (see Figure S33; cf. **2**$^{\cdot}$ [S6]). The isobars correspond to an endothermic process with ΔH = 31.8 kJ mol^{-1}. In the 315 K < T < 325 K range, a

noticeable deviation from the isobar occurs. Particularly, at 320 K, a time-dependent increase in the intensity of the EPR signal is observed together with its narrowing. The signal reaches its maximum after ~10 min at this temperature and then decreases. Subsequent cooling down to 210 K practically does not affect the intensity (see Figure S34a), which indicates an irreversible disintegration of the crystal structure of **3**˙. After heating in EPR spectrometer to 330 K, the crystalline solid visually turns out to be amorphous and exhibits gas-phase transfer into upper / colder parts of the EPR tube out of the EPR cavity (see Figure S34b). Subsequent cooling to 315 K leads to restoration of equilibrium. On further cooling to 210 K, the signal form remains unchanged whereas the signal intensity increases (see Figure S34a), most likely due to back gas-phase transfer of the compound from upper parts of the EPR tube to lower ones. These observations agree with irreversible disintegration of the crystal lattice of **3**˙ observed with variable-temperature powder XRD suggesting that crystalline **3**˙ becomes amorphous at 323 K (see Section S2, Figure S1).

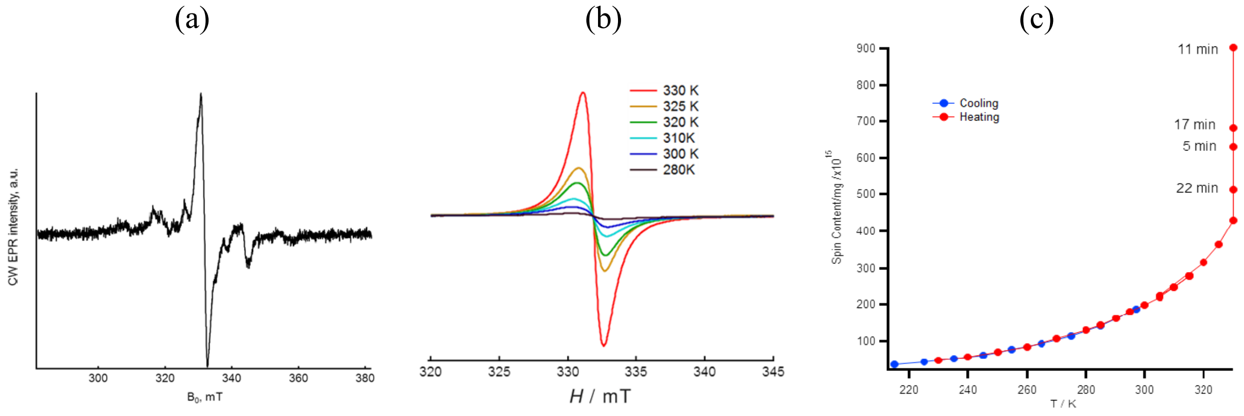

Figure S32. (*a*) Anisotropic EPR signal of **3**˙ at 195 K. (b) Color-indicated variable-temperature powder EPR spectra of **3**˙. (*c*) Temperature dependence of the spin content N_T in the sample of **3**˙ (weight 1.251 mg) in the range 185 K < T < 330 K determined by the double resonator method[S7] using calibration with deuterated 3-(piperid-1-yl)-2,2,5,5-tetramethyl-pyrroline-1-oxyl stable radical[S8] with a known number of spins (241×10^{15}) as a standard. At 295 K, the exact N_T of **3**˙ is 165×10^{15}. The double integral of the etalon EPR signal was assigned to the number of spins in the etalon. Since the Q-factor of the resonator does not change significantly in the 220 < T < 330 K range, the temperature dependence of the observed double integrals relates to the corresponding temperature change in N_T.

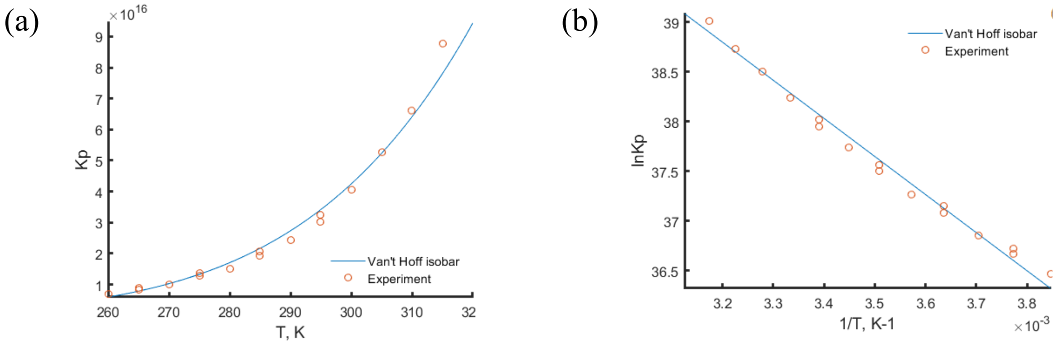

Figure S33. Van’t Hoff isobars of $\mathbf{3}_2 \leftrightarrow 2\ \mathbf{3}^{\cdot}$ process in the 260 < T < 315 K range as (a) K_p–T coordinate and (b) $\ln K_p$–T^{-1} coordinate.

(a) (b)

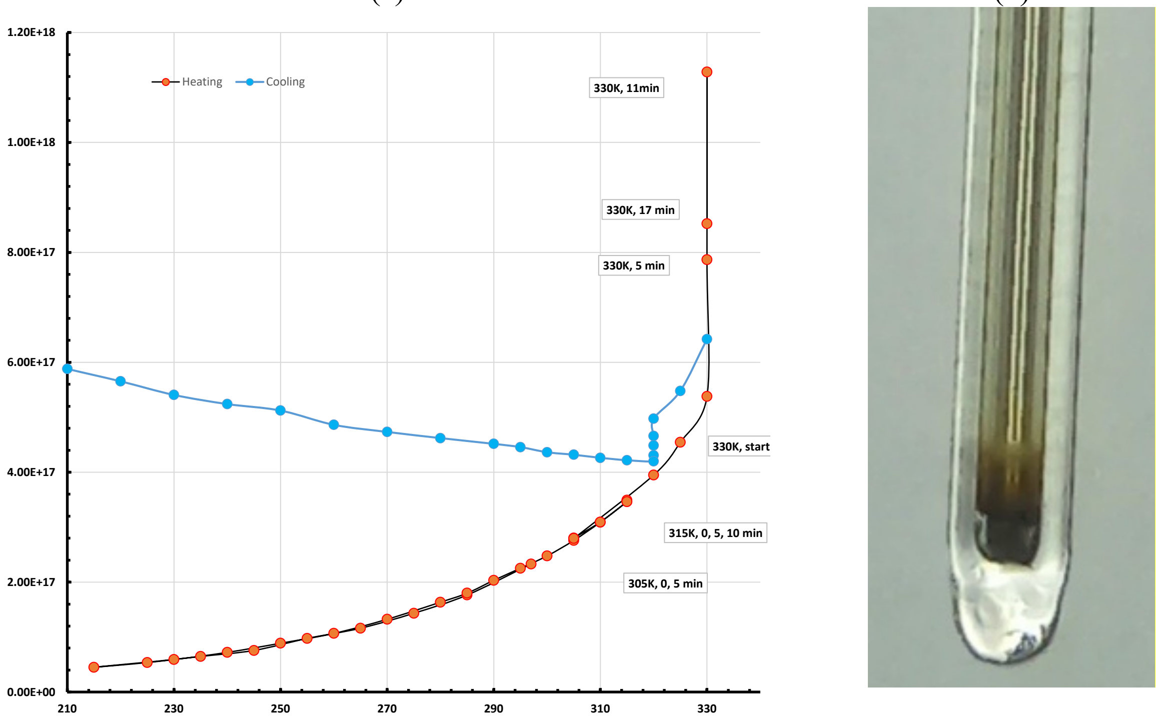

Figure S34. (a) Full version of the temperature dependence of the spin content of **3**˙ (sample weight 1.251 mg) in the 185 < T < 330 K range; red – heating, blue – cooling. (b) Image of the sample of **3**˙ after heating in EPR spectrometer to 330 K exhibiting transformation of the initial crystalline solid into amorphous one.

Variable-temperature powder EPR spectra of **4**˙ (Figures S35-S37) were measured simultaneously with those of Mn^{2+} in MgO standard in one-day experiment. In all measurements, powder **4**˙ (sample weight 0.908 mg) was sealed in an inert atmosphere into glass tube with inner diameter of 1 mm. The measurements were started at 295 K, and then the sample was cooled to 195 K and, after that, heated to 310 K, at which spectra were recorded after 5 min and 1 h storage. The EPR lines of **4**˙ were normalized with the intensity of the second EPR line of Mn^{2+} having in the measurements the magnetic field position $H_{(2)}$ = 321 mT. Compared with EPR spectrum of $CuCl_2{\cdot}2H_2O$ standard with known weight, the intensity of the second EPR line of Mn^{2+} corresponded to 469×10^{12} radicals S = 1/2, which was used to calculate the concentration of spins in the sample of **4**˙. The spectra were approximated by the sum of two Lorentzian functions $L_n(H)$ and $L_b(H)$ associated with narrow and broad components (see Figure S35a), whose contributions were characterized by their integral intensities $N_n \sim \int L_n(H)dH$ and $N_b \sim \int L_b(H)dH$, respectively (for parameters $dL_n(H)/dH$ and $dL_b(H)/dH$, see Table S2):

$$I(H) \sim L_n(H) + L_b(H) = A_1 \cdot \frac{1}{1+\frac{4}{3}\left(\frac{H-H_c}{\Delta H_n}\right)^2} + A_2 \cdot \frac{1}{1+\frac{4}{3}\left(\frac{H-H_c}{\Delta H_b}\right)^2}$$

where H_c is the magnetic field of the center of the EPR line, the value corresponds to g-value of Mn^{2+}. The values ΔH_n and ΔH_b are peak-to-peak widths of the Lorentzian functions for narrow and broad components, correspondingly. This gives the lineshape approximation as follows:

$$I_{EPR}(H) \sim \frac{dL_n(H)}{dH} + \frac{dL_b(H)}{dH}$$

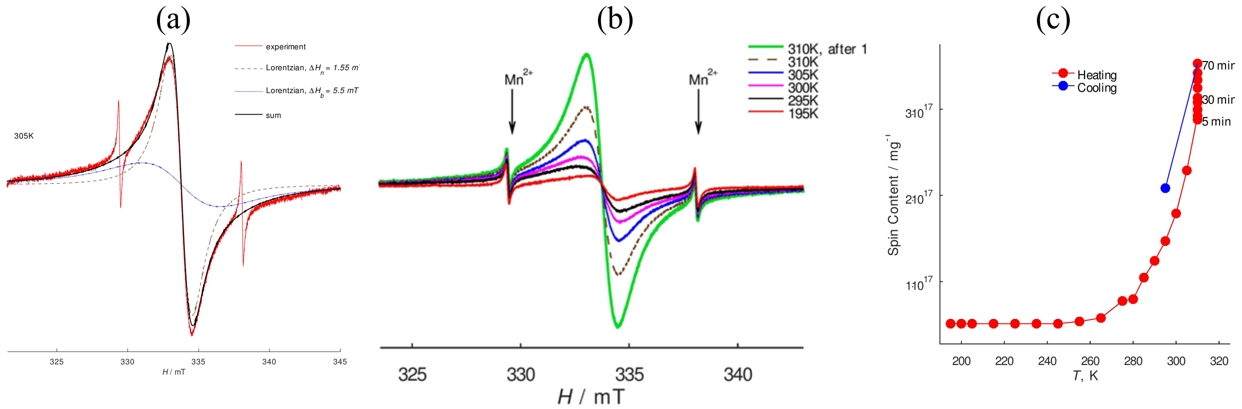

Figure S35. (a) Experimental powder EPR spectrum of **4**˙ (red) at 305 K and its Lorentzian approximation; the ΔH of the broad and narrow components are taken as 5.50 and 1.55 mT, respectively. (b) Color-indicated variable-temperature powder EPR spectra of **4**˙ in 195 < T < 310 K range, arrows indicate the third and fourth EPR lines of the Mn^{2+} standard. (c) Temperature dependence of the spin content of **4**˙ in the range 185 < T < 310 K (sample weight 0.908 mg).

Table S2. Simulation parameters for powder EPR spectra of 4˙ in the 195-310 K range

Temperature, K	Storage time at the temperature, min	ΔH_n, mT[a]	N_b/N_n
195			
205			
215			
225	5	1.35	3.6
235			
245			
255			2.9
265			2.3
275			3.1
280		1.6	2.8
285			3.9
290	10		4.2
295		1.7	3.9
295[b]		–	0.92
300		1.7	2.9
305		1.55	2.7
	5		2.3
310	30	1.45	1.6
	70		1.3

[a] ΔH_b of the broad component was set as 5.5 mT.
[b] For the spectrum obtained at 295 K after cooling from 70-min storage at 310 K

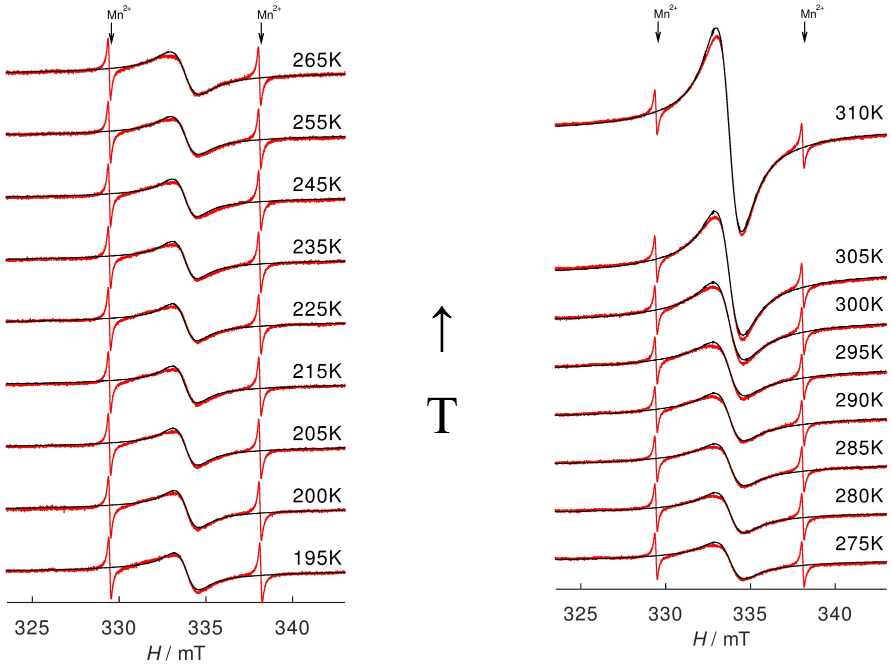

Figure S36. Experimental powder EPR spectra of **4**$^{\cdot}$ within 195 < T < 310 K (red, collected in one-day experiment), and their numerical simulations (black; for parameters, see Table S2). Initial spectrum was obtained at 195 K after the sample cooling from 295 K. Each next spectrum was measured after gradual heating to specified temperatures; that at 310 K was obtained after 5 min storage at this temperature.

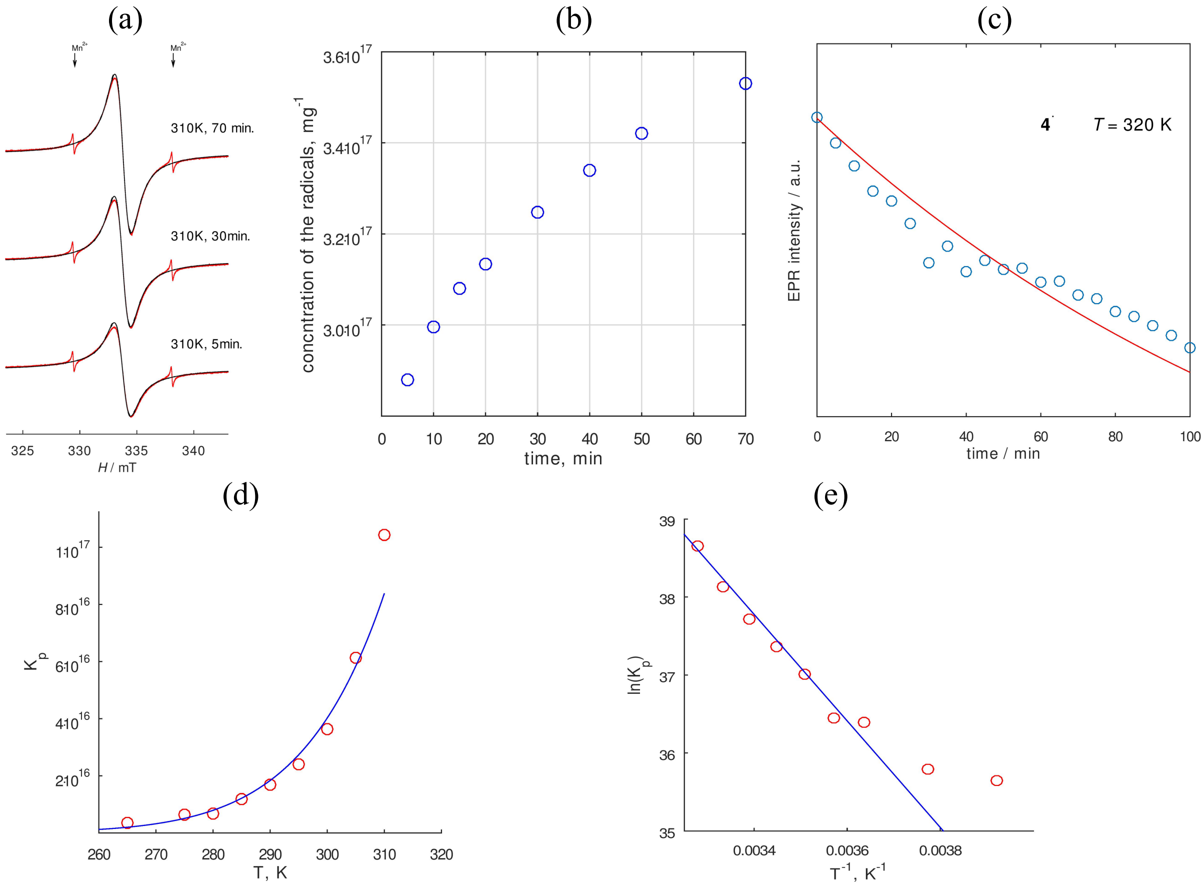

Figure S37. (a) Transformations of the powder EPR spectrum of **4**$^{\cdot}$ during storage at 310 K (red) and their approximations (black). (b) Time-dependent increase of spin concentration upon storage of **4**$^{\cdot}$ at 310 K. (c) Time-dependent decrease of spin concentration upon storage of **4**$^{\cdot}$ at 320 K. Van't Hoff isobar of $\mathbf{4}_2 \leftrightarrow 2\,\mathbf{4}^{\cdot}$ process within 260 < T < 310 K as (d) $K_p - T$ and (e) $ln\ K_p - T^{-1}$ coordinates.

Radical **4**$^{\cdot}$ is EPR-silent below 275 K but EPR-active at higher temperatures displaying a rapid increase of signal intensity between 300 and 310 K (see Figures S35b,c and S36). When stored at 310 K, spin concentration in the sample increases (Figure S37b) up to 3.54×10^{17} mg^{-1} after 70 min. At 320 K, decomposition of **4**$^{\cdot}$ occurs evidenced by slowly decreasing of the intensity of EPR signal with time (see Figure S37c). This agrees with variable-temperature powder XRD data suggesting that crystalline **4**$^{\cdot}$ becomes amorphous on keeping at 315 K (see Section S2, Figure S1). In the $260 < T < 310$ K range, the solid-state $\mathbf{4}_2 \leftrightarrow 2\,\mathbf{4}^{\cdot}$ diamagnetic ↔ paramagnetic equilibrium can be described by the van't Hoff isobar with $\Delta H = 56.8$ kJ mol^{-1} (Figure S37d,e; cf. **3**$^{\cdot}$ in Figure S33).

S10. MAGNETOMETRY.

The magnetic susceptibility of the polycrystalline samples of **3**˙ and **4**˙ is measured with a Quantum Design MPMS*XL* SQUID magnetometer in the 2 > T > 300 K range with magnetic field of up to 5 kOe; temperature sweep rate was 10 K min^{-1}. Diamagnetic corrections were made using the Pascal's constants.[S9] Below 200 K, **3**˙ and **4**˙ are diamagnetic and analysis of the $\chi(T)$ dependencies allows to estimate the diamagnetic susceptibility (χ_D) as –80×10^{-6} cm^3 mol^{-1} for **3**˙ and –108×10^{-6} cm^3 mol^{-1} for **4**˙. To analyze the $\chi(T)$ dependencies (Figure S38), equation $\chi(T) = p \cdot 0.375/T + \chi_D$ is used. Effective magnetic moment values are calculated as: $\mu_{\text{eff}}(T) = \left[\frac{3k_B}{N_A \mu_B^2}\,\chi T\right]^{1/2} \sim (8\,\chi T)^{1/2}$.

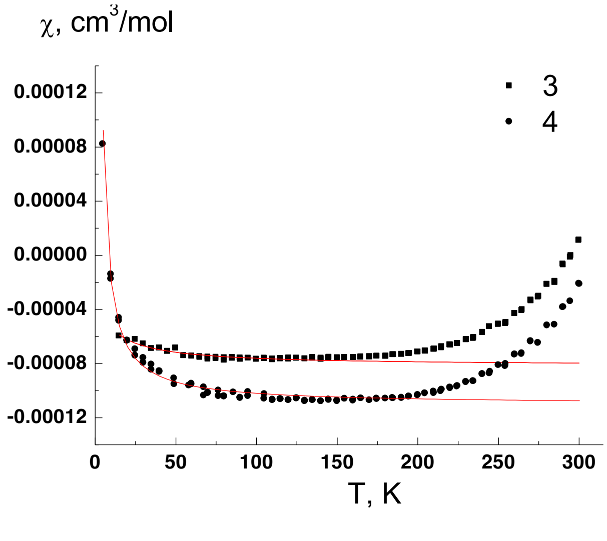

Figure S38. The χ(T) dependencies of **3**˙ (squares) and **4**˙ (circles).

Analysis of the $\mu_{\text{eff}}(T)$ for **3**˙ and **4**˙ (see Figure S39a) using the equation $\mu_{\text{eff}}^2 = p \cdot \mu_{\text{eff},Adm}^2 + \omega_{HS} \cdot \mu_{\text{eff},\boldsymbol{R}^\cdot}^2$ allows to estimate a fraction of the paramagnetic state ω_{HS} (see Figure S39b). Here, p is the number of paramagnetic defects; $\mu_{\text{eff},Adm}$ refers to admixture, and $\mu_{\text{eff},\boldsymbol{R}^\cdot}$ to **R**˙. Note that $\mu_{\text{eff},Adm} = \mu_{\text{eff},\boldsymbol{R}^\cdot} = 1.73$ μB is the theoretical μ_{eff} value for a monoradical. The residual values of the μ_{eff} below 200 K are attributed to paramagnetic defects, whose amounts are estimated as 17% for **3**˙ and 22% for **4**˙. At higher temperatures up to 300 K, effective magnetic moment increases almost identically for both **3**˙ and **4**˙. Changes of the μ_{eff} in the 200 < T < 300 K range are reversible, and the heating and cooling $\mu_{\text{eff}}(T)$ curves practically coincide (Figure S39a). Thus, both **3**˙ and **4**˙ are stable up to 300 K, and changes in the $\mu_{\text{eff}}(T)$ curves are indicative of temperature-dependent shift between diamagnetic and paramagnetic states. Similarly to **2**˙,[S6] at

300 K, μ_{eff} reaches values corresponding to 7% of the paramagnetic state of **3**$^{\cdot}$ and **4**$^{\cdot}$ in the samples. Bearing in mind that ω_{HS} is the paramagnetic state fraction (Figure S39b), the temperature-dependent solid-state diamagnetic ↔ paramagnetic equilibrium for both **3**$^{\cdot}$ and **4**$^{\cdot}$ may be described by equilibrium constants $K_{eq} = \omega_{HS}/(1 - \omega_{HS})$, that obey the van't Hoff isobar $\ln(K_{eq}) = -\Delta H/RT + \Delta S/R$ in the $200 < T < 300$ range (Figure S39c). The best fit values are $\Delta H = 17.51$ kJ mol^{-1} and $\Delta S = 36.1$ kJ K^{-1} mol^{-1} for **3**$^{\cdot}$, and $\Delta H = 14.88$ kJ mol^{-1} and $\Delta S = 26.8$ kJ K^{-1} mol^{-1} for **4**$^{\cdot}$. For comparison, **5**$^{\cdot}$: $\Delta H\downarrow = 1.41$ kJ mol^{-1}, $\Delta S\downarrow = 6.0$ kJ K^{-1} mol^{-1}; $\Delta H\uparrow = 1.86$ kJ mol^{-1}, $\Delta S\uparrow = 5.8$ kJ K^{-1} mol^{-1}.[S10]

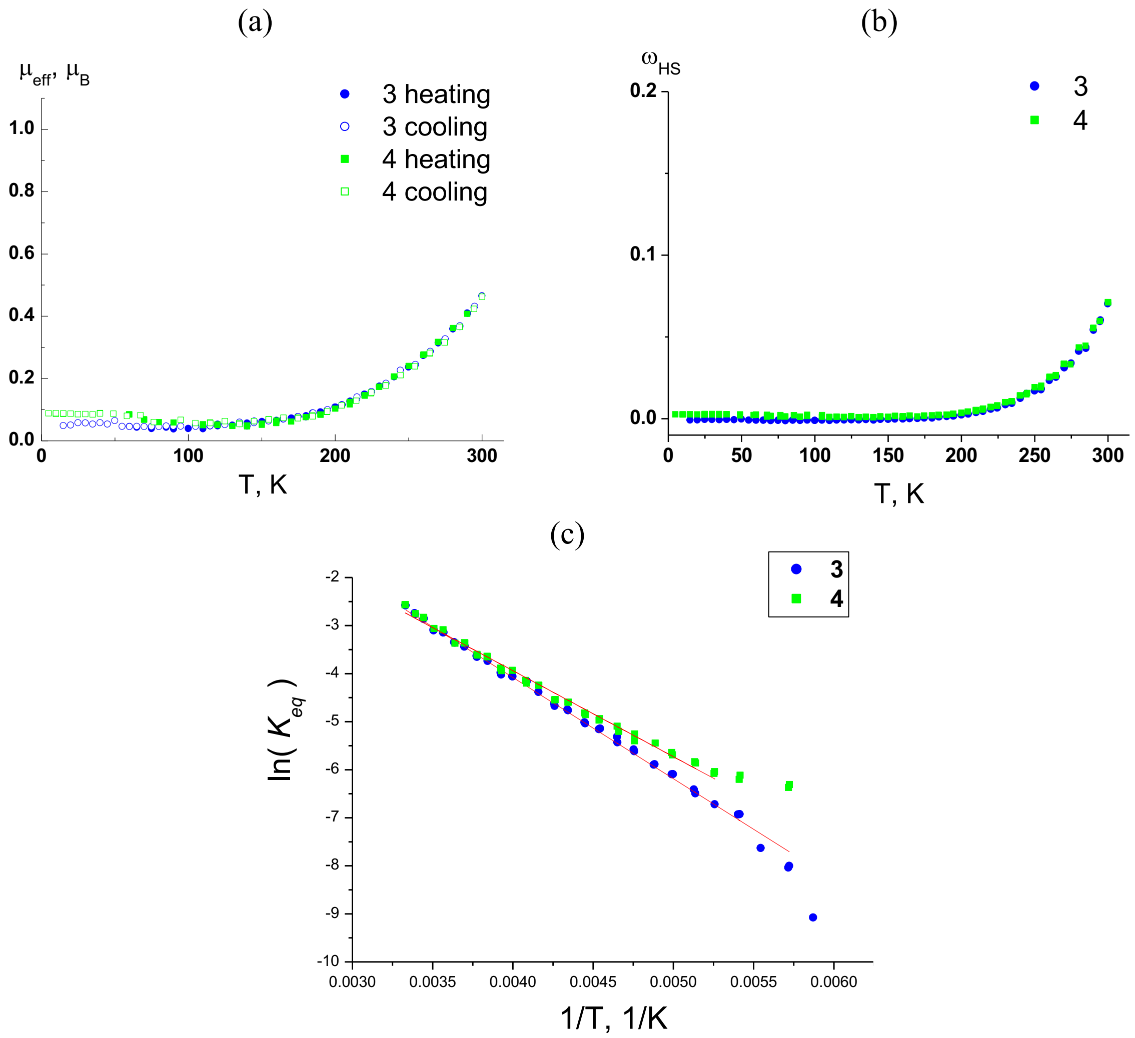

Figure S39. (a) Effective magnetic moment μ_{eff} of **3**$^{\cdot}$ and **4**$^{\cdot}$ as a function of temperature in the $2 < T < 300$ K range. (b) Paramagnetic state fraction ω_{HS} for **3**$^{\cdot}$ and **4**$^{\cdot}$ as a function of temperature. (c) Van 't Hoff isobar plots for the $200 < T < 330$ K range in $\ln K_p - T^{-1}$ coordinate.

S11. CRYSTAL PACKING SYNTHON ANALYSIS

Solid-state optimizations and interaction energy calculations are performed using the QuantumEspresso (QE) code[S11] with the (U)PBE functional, Grimme-D3 general dispersion correction, and ultrasoft pseudopotentials. For solid-state optimization purposes, supercells for LT and HT containing sixteen **R**˙ are used. The supercell used for LT optimizations is built using the XRD data and duplicating *a* and *b* unit cell parameters (2*a*, 2*b*, 1*c*), while for HT is built by replicating the unit cell four times along the *b* axis, *i.e.*, the new cell was (1*a*, 4*b*, 1*c*). Relaxation of the **2**˙-LT, **2**˙-HT and **3**˙ is carried out with fixed cell (FC) and variable cell (VC) cell optimizations. The FC optimizations employ the XRD cell parameters, while in VC optimizations the unit cell parameters relax. Optimization of pairs of **R**˙s to obtain interaction energies are also performed using periodic QE code to avoid BSSE (cell parameters are selected to have isolated pairs of **R**˙s).

To identify unique radical pairs for interaction energy calculations, the analysis of the crystal packing is performed. For **3**˙, a reference radical #1 is defined. The first- and second-neighboring radicals to #1 are then selected among all possibilities within 10.0 Å from #1. Nearest (next-nearest) neighbors to #1 belong to arrays numbered #2–#9 (#3', #6'–#9') (see Figure S40).

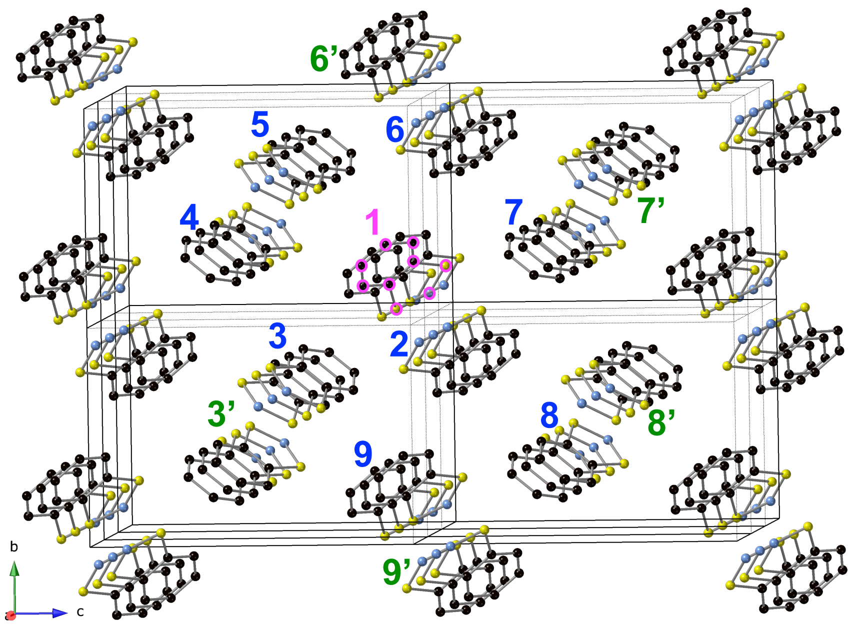

Figure S40. Crystal packing of **3**˙ (F atoms are omitted for clarity) displaying the reference **R**˙ radical (#1, in magenta), the first-nearest (#2–#9, in blue) and second-next-nearest neighboring radicals (#3', #6'–#9', in green).

From all possibilities, the pairs of **R**˙s with largest interaction energy are those involved in the σ four-center-mediated zip-π-stack synthon, *i.e.*, the first neighboring radicals to #1 (see Figure S41). The building blocks of the synthon are a π-stacked head-over-tail pair (–47.03 kJ mol^{-1}), an offset π-stacked head-over-head pair (–17.60 kJ mol^{-1}), and a 4-center coplanar head-to-tail pair (–15.76 kJ mol^{-1}) (Figure S41c-e). In addition, there are ten different pairs of radicals that belong to next-nearest neighboring **R**˙s to #1 (Figure S42), *i.e.*, that establish SBIs with nearby synthons: one coplanar head-to-tail lateral pair [–3.34 (1) kJ mol^{-1}], three interplanar head-over-tail (parallel π-stack) lateral pairs [–8.36 (1), –0.59 (1), and –0.42 (1) kJ mol^{-1}], and six orthogonally arranged pairs [–10.03 (2), –7.40 (2), –4.60 (2), –3.64 (2), –0.46 (2) and –0.38 (2) kJ mol^{-1}]. Therefore, ten pairs of radicals establish sixteen SBIs per reference radical **R**˙.

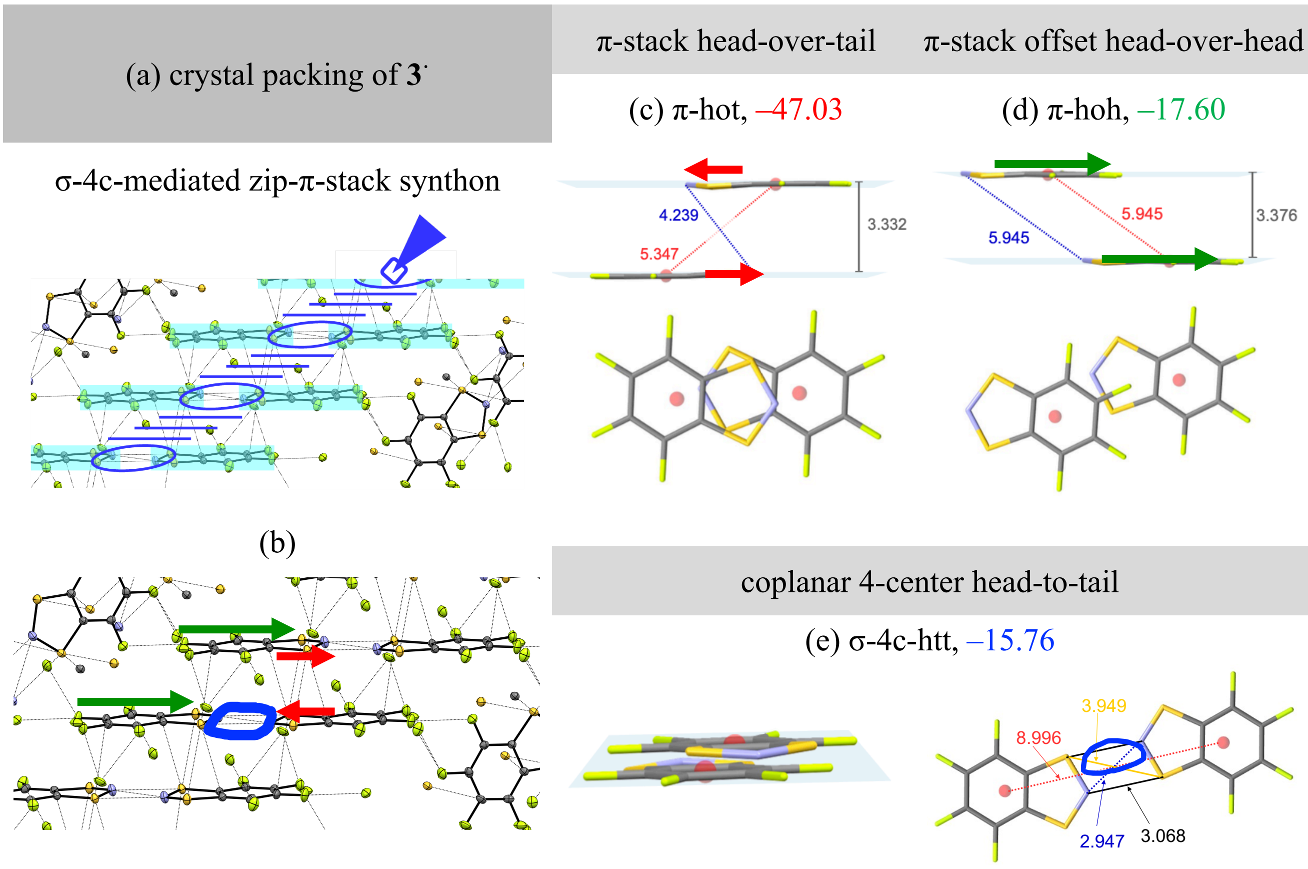

Figure S41. (a) Schematic representation of the σ-4-center-mediated zip-π-stack synthon of **3**˙ with (b) corresponding building blocks (in red, green and blue), namely, (c) a π-stacked head-over-tail pair (–47.03 kJ mol^{-1}, in red), (d) an offset π-stacked head-over-head pair (–17.60 kJ mol^{-1}, in green), and (e) a coplanar 4-center head-to-tail pair (–15.76 kJ mol^{-1}, in blue). Interaction energies E_{int} are given in kJ mol^{-1} for each radical pair.

coplanar head-to-tail lateral	interplanar head-over-tail parallel π-stack lateral	
(a) co-htt-lat, –3.34	(b) i-hot-par-π-lat 1, –8.39	(c) i-hot-par-π-lat 2, –0.59

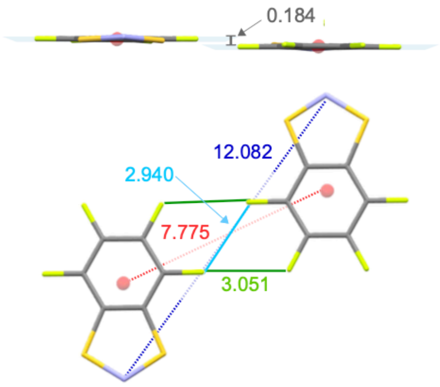

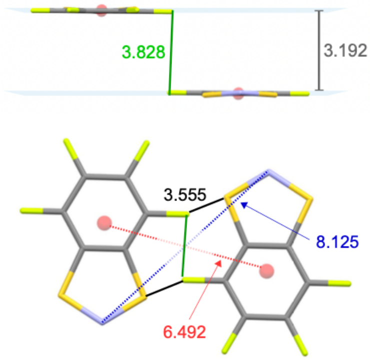

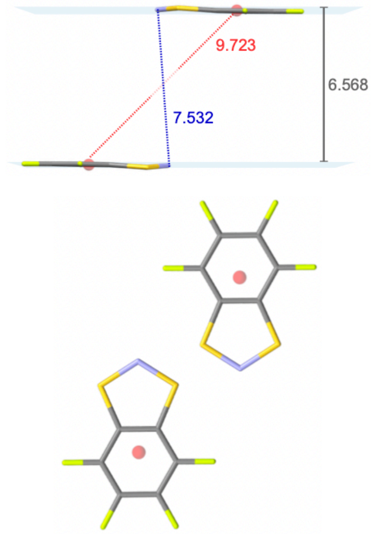

interplanar head-over-tail (parallel π-stack) lateral	orthogonal radical arrangement
(d) i-hot-par-π-lat 3, –0.42	(e) orthogonal 1, –10.03

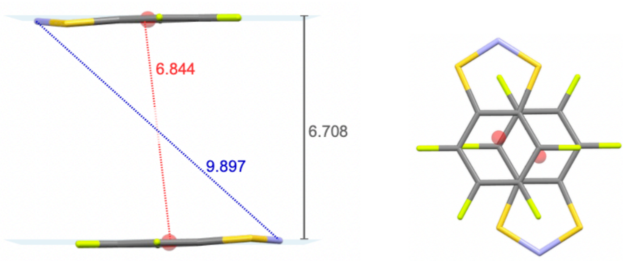

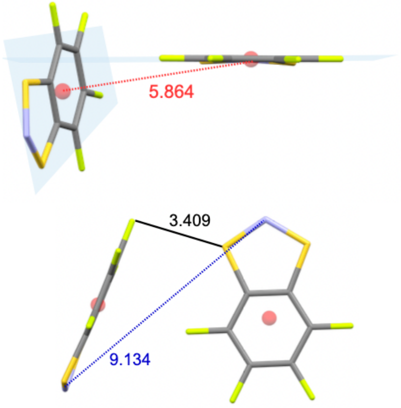

orthogonal radical arrangement		
(f) orthogonal 2, –7.40	(g) orthogonal 3, –4.62	(h) orthogonal 4, –3.64

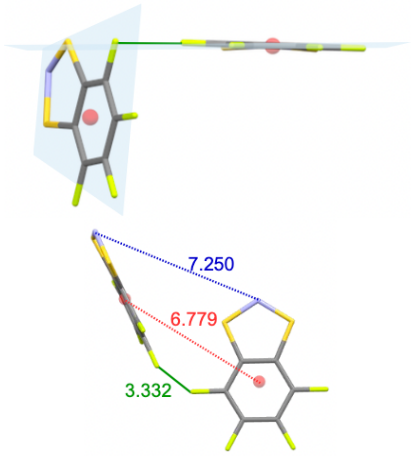

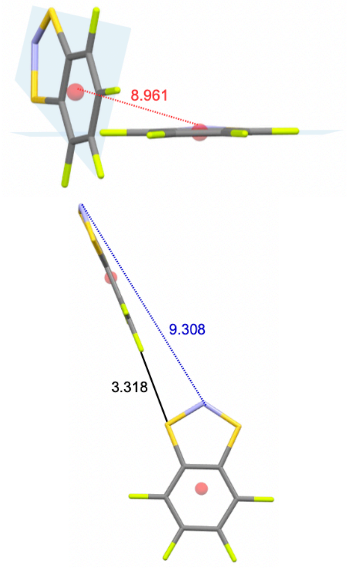

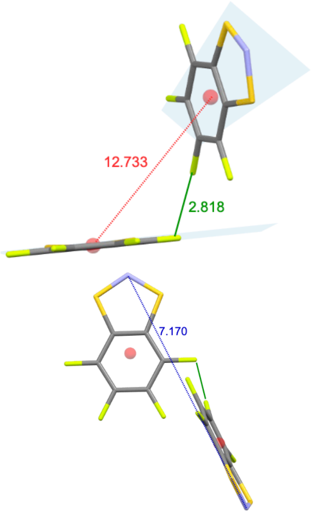

orthogonal radical arrangement

(i) orthogonal 5, –0.46 | (j) orthogonal 6, –0.38

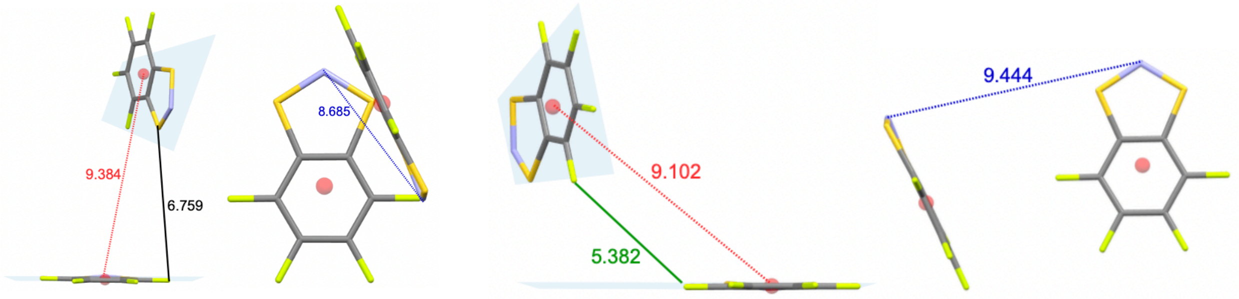

Figure S42. Pairs of radicals between reference radical **R**$^{\cdot}$ #1 and next-nearest neighboring radicals to #1, together with interaction energies E_{int} (kJ mol^{-1}): (a) coplanar head-to-tail lateral pair, (b-d) interplanar head-over-tail (parallel π-stack) lateral pairs, and (e-j) orthogonally arranged pairs.

(a) crystal packing of **2**$^{\cdot}$-LT | π-stack head-over-head

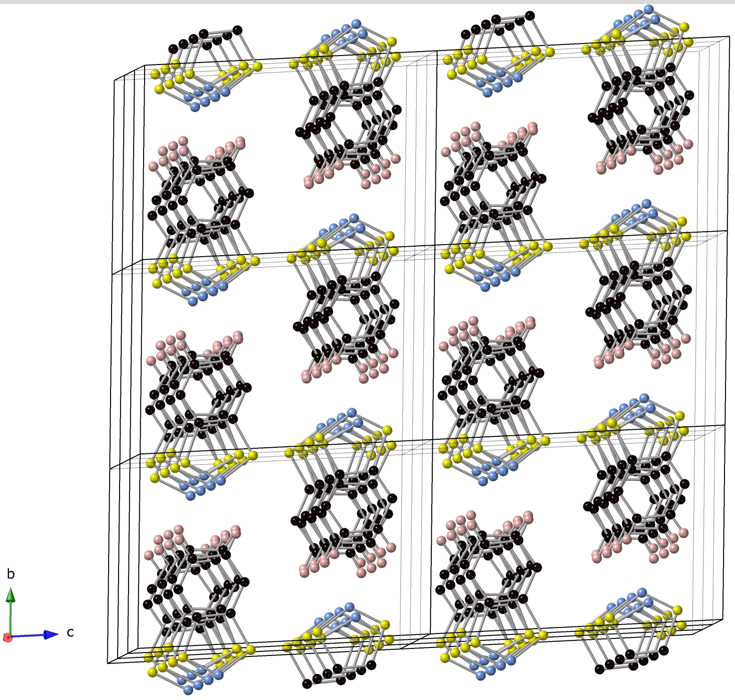

(b) eclipsed, π-hoh-e, –45.19

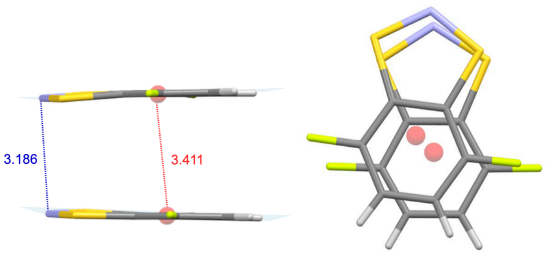

(c) slipped, π-hoh-s, –27.38

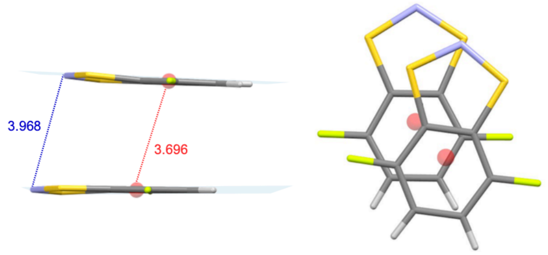

Figure S43. (a) Representation of the alternant 1D π-stack (along *a*-axis) synthon of **2**$^{\cdot}$-LT with corresponding building blocks: (b) a π-stacked head-over-head eclipsed pair (–45.19 kJ mol^{-1}), and (c) a π-stacked head-over-head slipped pair (–27.38 kJ mol^{-1}). Interaction energies E_{int} are given in kJ mol^{-1} together with the radical pair.

For **2**$^{\cdot}$**-LT** (Figure S43), the synthon consists of an alternant 1D π-stack of head-over-head pairs of radicals: one of them is practically eclipsed (π-hoh-e, Figure S43b) and the other displays certain degree of latitudinal slippage (π-hoh-s, Figure S43c). Any given radical in **2**$^{\cdot}$-LT establishes only one π-hoh-e and one π-hoh-s along the synthon. However, it exhibits as many as ten different

lateral contacts between synthons (Figure S44): two coplanar head-to-tail lateral pairs [–8.61 (1) and –8.49 (1) kJ mol^{-1}], six interplanar head-over-tail (parallel π-stack) lateral pairs with distinct degrees of latitudinal and longitudinal slippage [–10.95 (1), –8.61 (1), –8.45 (1), –8.45 (1), –5.56 (1), and –2.22 (1) kJ mol^{-1}], and two interplanar head-to-head (parallel π-stack) lateral pairs with considerable longitudinal slippage [–11.45 (2) and –8.36 (2) kJ mol^{-1}]. In this case, ten pairs of radicals establish twelve SBIs per reference radical **R**$^{\cdot}$. This number is smaller than those belonging to **3**$^{\cdot}$, which means that the 1D π-stack arrangement involves less inter-synthon contacts than the 4-center mediated zip-π-stack synthon.

coplanar head-to-tail lateral

(a) co-htt-lat 1, –8.61

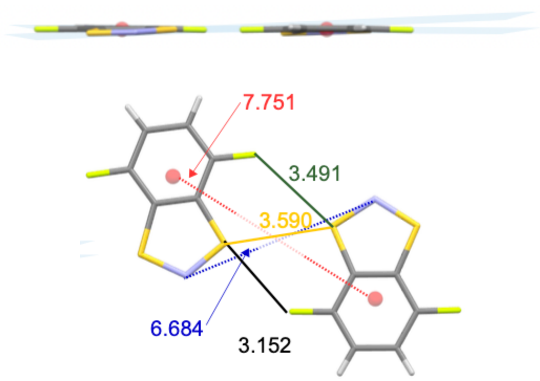

(b) co-htt-lat 2, –8.49

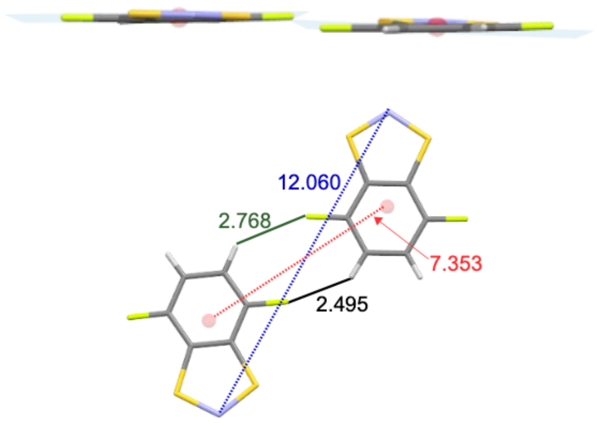

interplanar head-over-tail (parallel π-stack) lateral

(c) i-hot-par-π-lat 1, –10.95

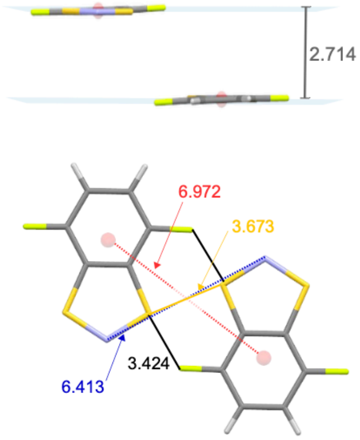

(d) i-hot-par-π-lat 2, –8.61

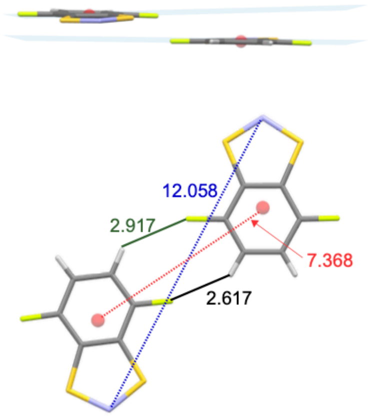

(e) i-hot-par-π-lat 3, –8.44

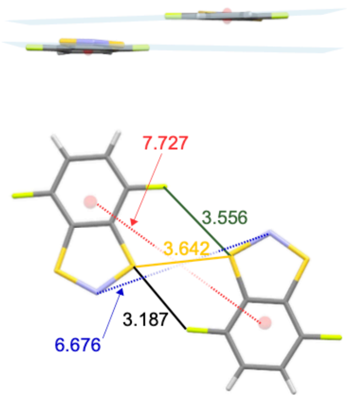

interplanar head-over-tail (parallel π-stack) lateral

(f) i-hot-par-π-lat 4, –8.44

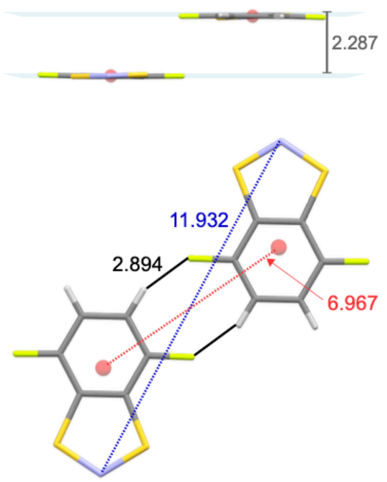

(g) i-hot-par-π-lat 5, –5.56

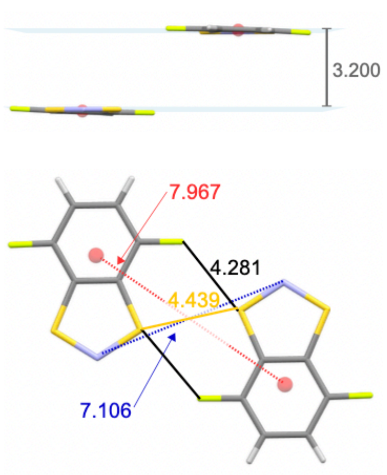

(h) i-hot-par-π-lat 6, –2.22

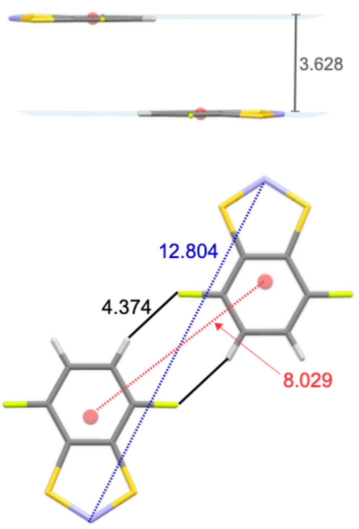

interplanar head-over-head (parallel π-stack) lateral

(i) i-hoh-par-π-lat 1, –11.45

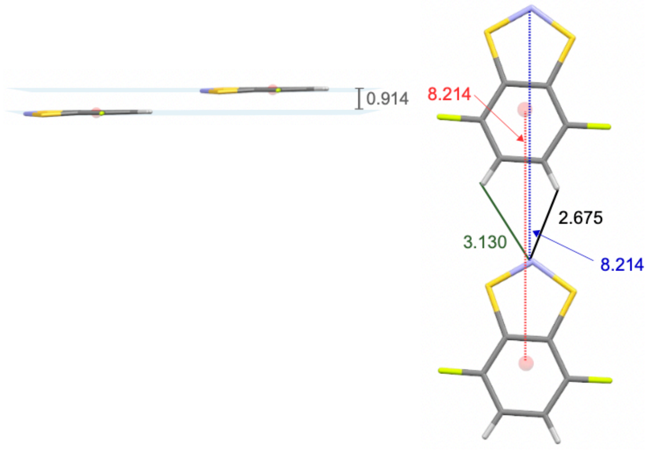

(j) i-hoh-par-π-lat 2, –8.36

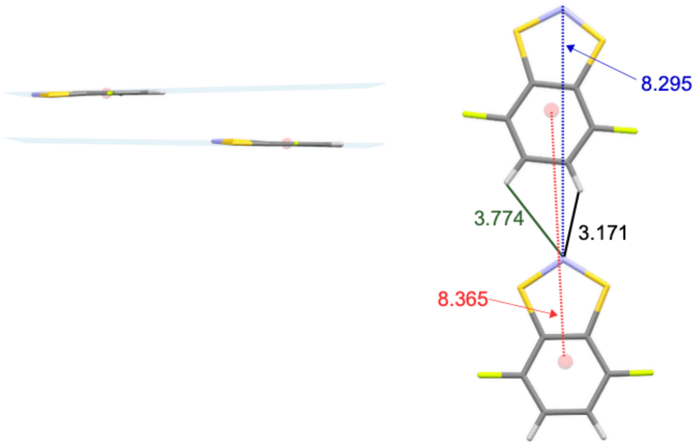

Figure S44. Pairs of radicals between reference radical **R**$^{\cdot}$ #1 of **2**$^{\cdot}$-LT and next-nearest neighboring radicals to #1, together with interaction energies E_{int} (kJ mol^{-1}): (a, b) coplanar head-to-tail lateral pairs, (c-h) interplanar head-over-tail (parallel π-stack) lateral pairs, and (i, j) interplanar head-over-head (parallel π-stack) lateral pairs.

Similar to **2**$^{\cdot}$-LT, the synthon of **2**$^{\cdot}$-HT consists of a regular 1D π-stack of head-over-head pairs of radicals displaying certain degree of latitudinal slippage (π-hoh-reg) (Figure S45b). Since the π-array is regular, any given radical **R**$^{\cdot}$ establishes two π-hoh-reg pairs along the synthon and the number of lateral interactions connecting different π-stacks is thus reduced to eight. These lateral contacts (Figure S45c-j) include: four interplanar head-over-tail (parallel π-stack) lateral pairs [–9.41 (1), –9.07 (1), –6.94 (1), and –2.59 (1) kJ mol^{-1}), two interplanar head-to-tail tilted-π-stack lateral pairs [–8.69 (2) and –8.11 (2) kJ mol^{-1}) and two interplanar head-to-head tilted-π-

stack lateral pairs [–10.49 (2) and –8.53 (2) kJ mol^{-1}). In this case, eight pairs of radicals establish twelve SBIs per reference radical **R**$^{\cdot}$, as in **2**$^{\cdot}$-LT, meaning again that the 1D π-stack arrangement involves less inter-synthon contacts than the four-center mediated zip-π-stack synthon.

(a) crystal packing of **2**$^{\cdot}$-HT	π-stack head-over-head regular

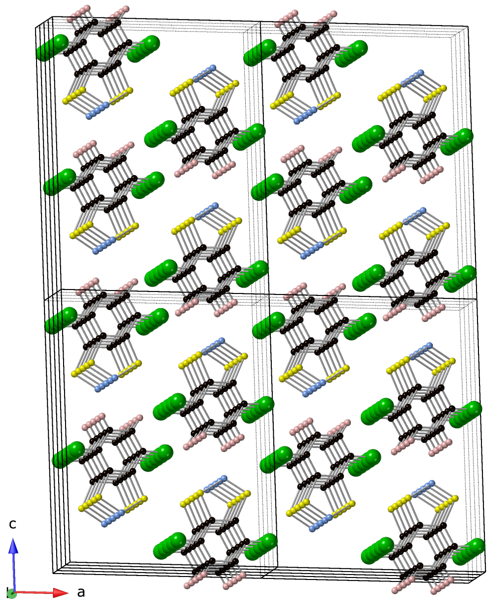

(b) π-hoh-reg, –31.68

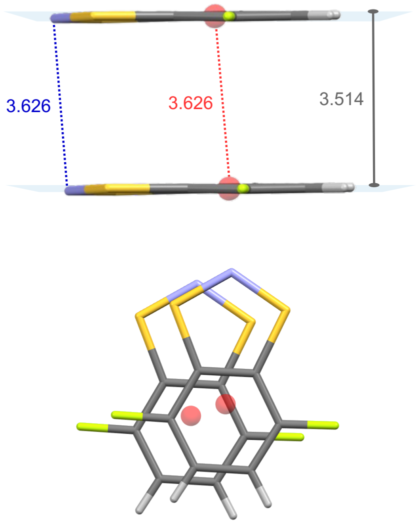

interplanar head-over-tail (parallel π-stack) lateral

(c) i-hot-par-π-lat 1, –9.41

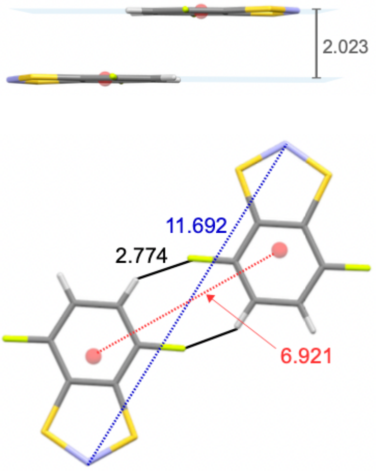

(d) i-hot-par-π-lat 2, –9.07

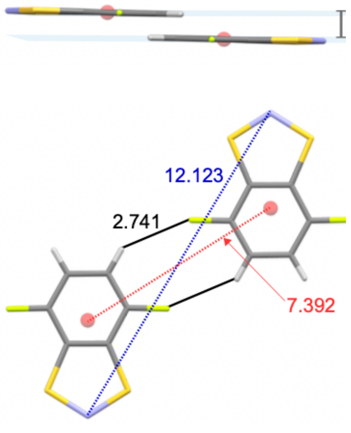

(e) i-hot-par-π-lat 3, –6.94

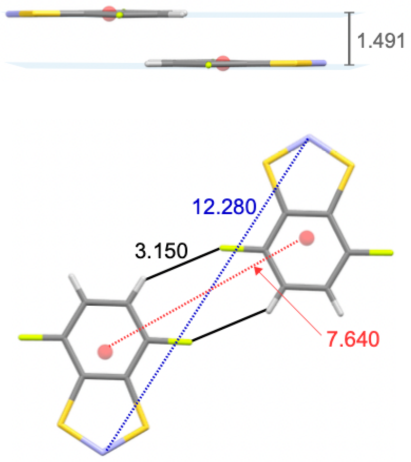

interplanar head-over-tail, lateral

parallel π-stackl

tilted-π-stack

(f) i-hot-par-π-lat 4, –2.59

(g) i-htt-tilt-π-lat 1, –8.69

(h) i-htt-tilt-π-lat 2, –8.11

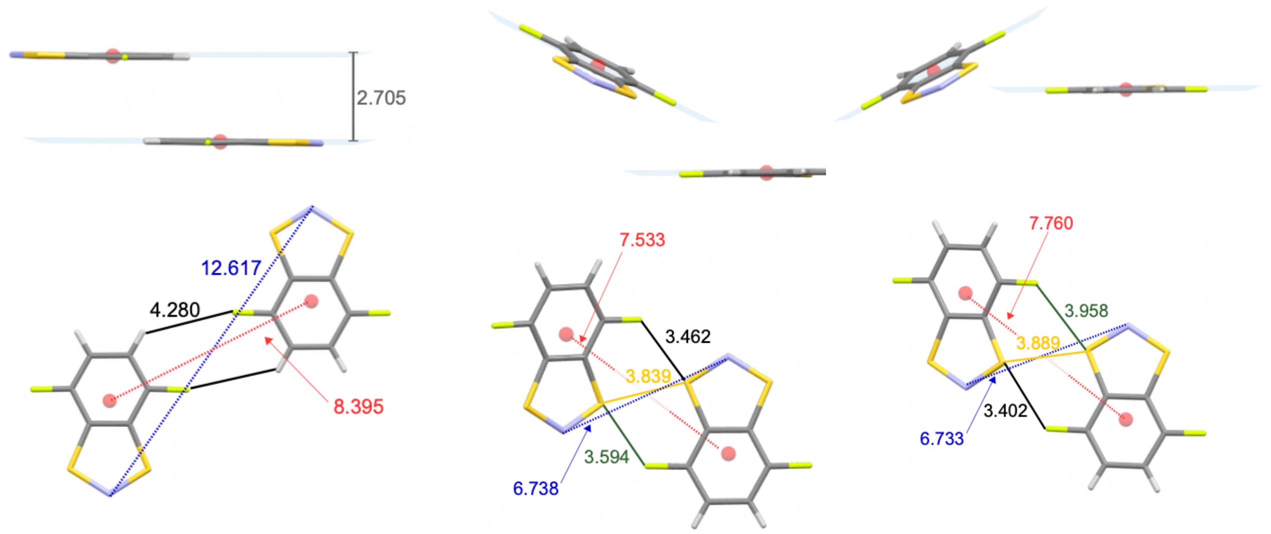

interplanar head-to-head tilted-π-stack, lateral

(i) i-hth-tilt-π-lat 1, –10.49

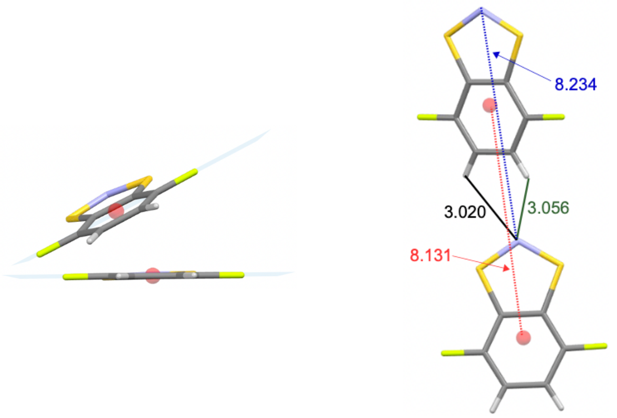

(j) i-hth-tilt-π-lat 2, –8.53

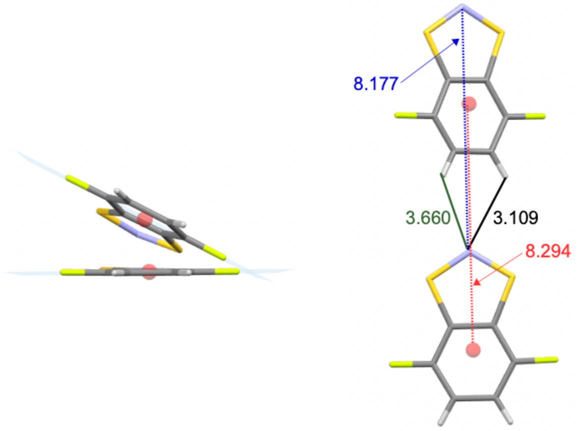

Figure S45. (a) Representation of the regular 1D π-stack (along *b*-axis) synthon of **2**˙-HT with corresponding building block: (b) a π-stacked head-over-head pair (–31.68 kJ mol^{-1}). Pairs of radicals between reference radical #1 of **2**˙-HT and next-nearest neighboring radicals to #1, together with interaction energies E_{int} (kJ mol^{-1}): (c-f) interplanar head-over-tail (parallel π-stack) lateral pairs, (g, h) interplanar head-over-tail (tilted π-stack) lateral pairs, and (i, j) interplanar head-over-head (tilted π-stack) lateral pairs.

S12. HIRSHFELD SURFACE ANALYSIS

Whereas synthon approach operates with molecular ensembles, Hirshfeld surface analysis[S12] is focused on the atom···atom intermolecular contacts. These approaches are each other complementary to give jointly more complete pictures of how molecules assemble in molecular crystals.[S13,S14]

Hirshfeld surface analysis is based on the property d_{norm}, whose negative values indicate that the spatial separation between the closest atoms within and outside the so-called Hirshfeld surface is less than the sum of the corresponding Van der Waal (VdW) radii. Positive/negative values denote separations lesser/greater than the sum of VdW radii (Figure S46, d_{norm} values for all the surfaces: min –0.146, max: 1.0.96 Å, white regions have near-equal distances). The Hirhsfeld surface analysis combined with 2D-fingerprint plots corroborates that **2**˙-HT and **2**˙-LT display two distinct interaction patterns, which can be matched to two different HT (328 K) and LT (180 K) phases (Figures S46 and S47). In contrast, **3**˙ at 296 K and 200 K demonstrate coincident Hirshfeld surfaces and fingerprint patterns, suggesting that no significant packing alterations occur in response to temperature variation (Figures S46 and S47). Hereafter only **3**˙ at 200 K will be discussed. The same is concluded for **4**˙.

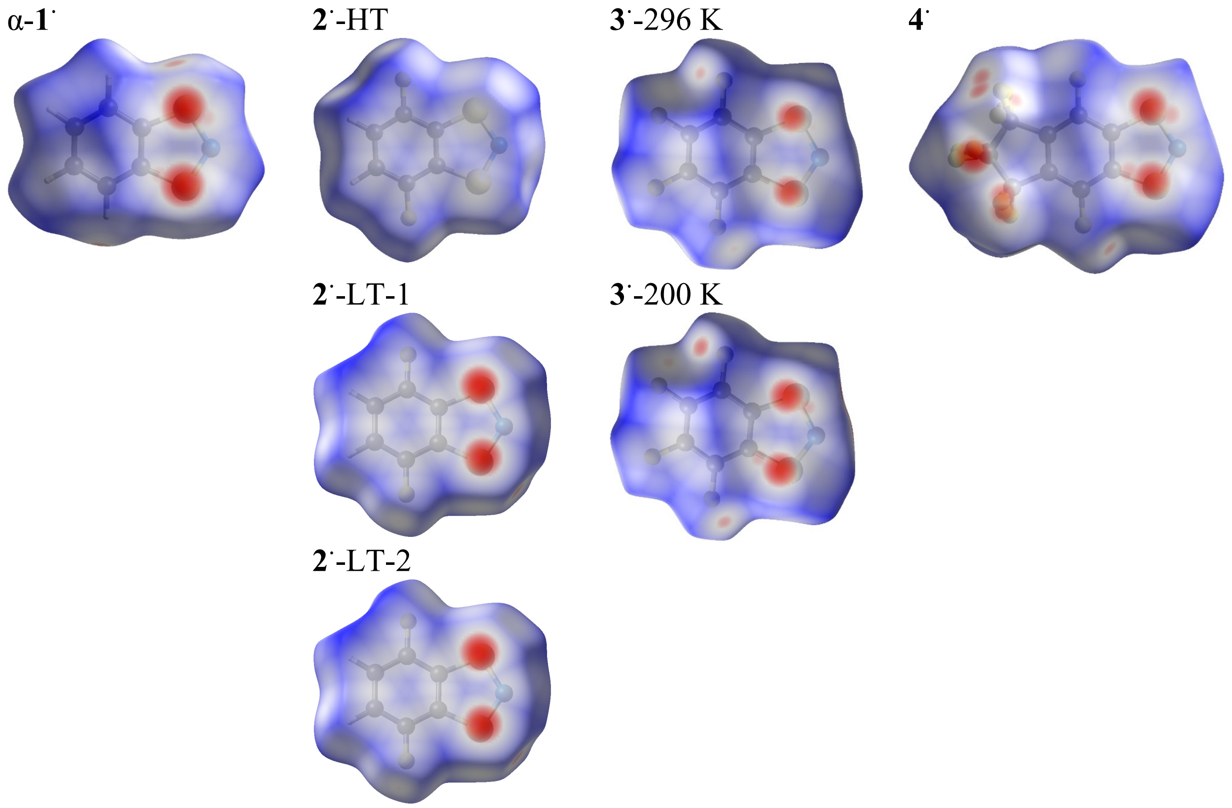

Figure S46. Hirshfeld surfaces of **1**˙-**4**˙. Regions with negative / positive d_{norm} values are red / blue. HT and LT denote high- and low-temperature phases, respectively. For **2**˙-LT, the asymmetric unit contains two independent molecules labeled as 1 and 2.

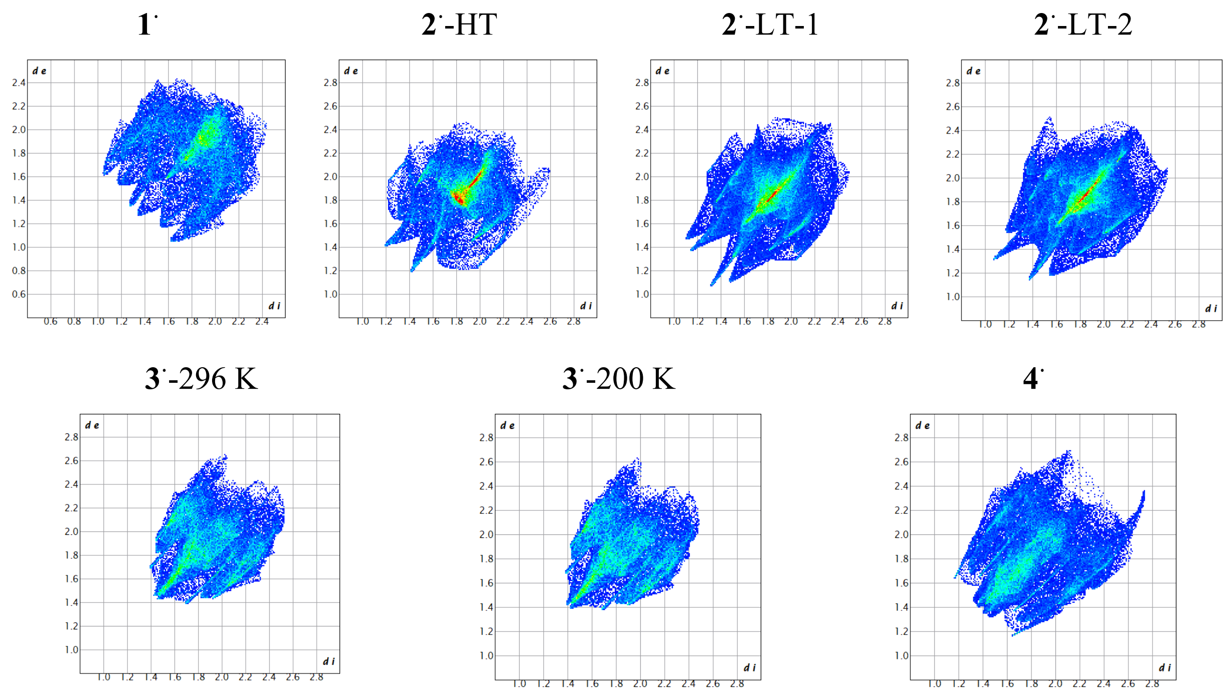

Figure S47. Hirshfeld 2D-fingerprints of **1**˙-**4**˙. For **2**˙-LT, the asymmetric unit contains two independent molecules labeled here 1 and 2. In contrast to different **2**˙-HT and **2**˙-LT phases, those of **3**˙-296 K and **3**˙-200 K are actually the same phase measured at different temperatures. The central red markers in the fingerprints of **2**˙ indicate π-stacking.

At this point, it would be very interesting to compare the values obtained using the Crystal Explorer program[S15] on interaction energy frameworks and their components (namely, $E_{tot} = E_{ele} + E_{pol} + E_{dis} + E_{rep}$, where E_{tot} is the total interaction energy; and its components are the electrostatic (*ele*), polarization (*pol*), dispersion (*dis*), and exchange-repulsion (*rep*) contributions, respectively) with the *ab initio* calculated interaction energies reported in Figures S40-S45 (SI Section S11). Let us stress that CrystalExplorer holds significant potential as a powerful tool to analyze energy frameworks. Yet, it is important to acknowledge the limitations of CrystalExplorer in the context of our study. The interaction energies it provides are derived from a model calibrated using dispersion-corrected DFT data for nitronyl-nitroxides and metal-based open-shell systems, which do not account for π-stacks of planar organic radicals. Notably, the CrystalExplorer documentation itself reports a mean absolute deviation of up to 2.4 kJ/mol for pairs of radicals that can be described with the calibration models and, in some cases, deviations may reach 4.7 kJ/mol. As such, the dispersion energy values obtained for our target benzo-fused dithiazolyl compounds should be regarded as approximate estimates. Consequently, although we did not expect our calculated interaction energies to match CrystalExplorer values numerically, we anticipated that

both approaches would reveal consistent qualitative trends for **2˙** and **3˙**, which would justify extending the analysis to **1˙** and **4˙** using CrystalExplorer data.

The CrystalExplorer (CE) interaction energy decomposition procedure estimates the total energy (E_{tot}) in terms of its electrostatic (E_{ele}), polarization (E_{pol}), dispersion (E_{dis}) and exchange-repulsion (E_{rep}) components. It must be stressed that total energies are the sum of these four energy components, scaled appropriately using scale factors for benchmarked energy models (see Table S3).[S15] For instance, CE-total energies can use two sets of scale factors (k) depending on whether the result is fitted to HF/3-21G or to B3LYP/6-31G(d,p) electron densities. Either way, as it will be here shown, the CE-polarization energies for pairs of organic radicals and, specifically for π-stacked radicals, do not capture the electronic nature of the interaction. They are not well described since the dataset used to extract the electron densities only accounts for very few families of open-shell systems and none of them accounts for π-stackings. Therefore, the CE-total energies cannot be trusted when referring to interactions between 1,3,2-benzodithiazolyls radicals.

Table S3. Scale factors of the electrostatic (k_{ele}), polarization (k_{pol}), dispersion (k_{dis}) and exchange-repulsion (k_{rep}) components of the total energy for benchmarked energy models.[S15]

Energy Model	k_{ele}	k_{pol}	k_{disp}	k_{rep}
CE-HF ... HF/3-21G electron densities	1.019	0.651	0.901	0.811
CE-B3LYP ... B3LYP/6-31G(d,p) electron densities	1.057	0.740	0.871	0.618

For **3˙**-200K, one molecule is designated as the reference, while color-coded interacting neighboring molecules (named 1 to 9 in Table S4) are analyzed for their interaction energies. Comparison of calculated interaction energies (E_{int}) and fitted CE-total energies (E_{tot}) shows that the energies qualitatively agree in all intra- and inter-synthon contacts with no π-stacking involved (3, 4, 6-9 in Table S4) or in which there is a large slippage between π-stacked radicals (2, 5 in Table S4). Yet, CE-total energies fail when the π-stacking between radical moieties is significant (1 in Table S4).

Table S4. Intra/inter-synthon interaction energies (in kJ mol^{-1}) of **3$^{\cdot}$–200K**, as calculated using the explicit DFT/PBE0 functional (E_{int}, QE PBE0 D3 level) or as fitted to the so-called CE-B3LYP benchmarked energy model. Interactions are listed as the distance between centroid···centroid of radicals increases (in Å): the intra-synthon are listed first, then the SBIs (d_{cent} as measured with Mercury[S16]). Note N stands for the number that a given interaction is univocally found given a reference radical **R$^{\cdot}$**, which is at the center of the below figure. E_{ele}, E_{pol}, E_{dis} and E_{rep} are given as absolute values without scale factor, whereas E_{tot} is the sum of the scaled[S15] components.

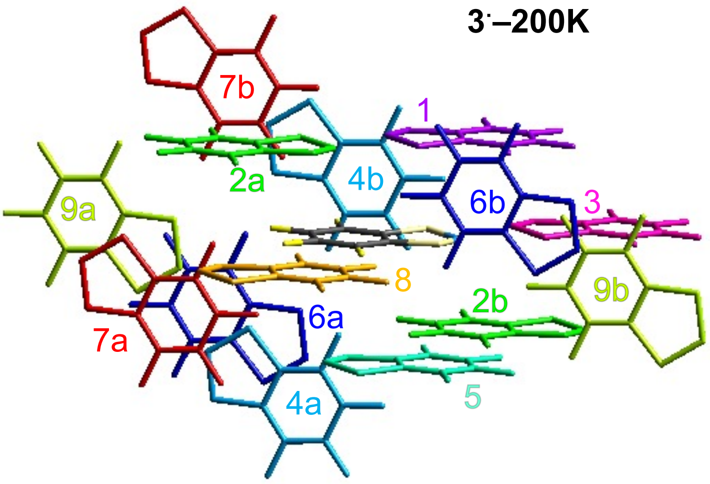

		Mercury					Crystal Explorer				
	N	d_{cent}	ref in Fig S59/S60	E_{int}		R	E_{ele}	E_{pol}	E_{dis}	E_{rep}	E_{tot}
	1	5.342	π-hot	-47.03	1	4.92	-32.8	-1.9	-36.4	80.4	-18.2
	2	5.945	π-hoh	-17.60	2a/2b	5.95	-6.0	-1.1	-19.7	11.7	-17.1
	1	8.996	σ-4c-htt	-15.76	3	8.46	-19.6	-2.4	-9.9	20.9	-18.2
	2	5.864	orthogonal 1	-10.03	4a/4b	5.95	-0.8	-0.6	-14.2	5.0	-10.5
	1	6.492	i-hot-par-π-lat 1	-8.39	5	6.40	-0.3	-0.2	-9.9	3.1	-7.2
	2	6.799	orthogonal 2	-7.40	6a/6b	6.82	-0.6	-0.3	-11.6	3.7	-8.7
	2	7.170	orthogonal 4	-3.64	7a/7b	7.58	-0.7	-0.3	-8.1	1.0	-7.4
	1	7.775	co-htt-lat	-3.34	8	8.01	0.9	-0.1	-6.8	0.3	-4.8
	2	8.961	orthogonal 3	-4.62	9a/9b	8.98	-0.9	-0.2	-4.7	1.6	-4.1

d_{cent} (in Å) measured using the cif file data with Mercury

R (in Å) as listed in the energy model output file of Crystal Explorer

For **2$^{\cdot}$-HT**, the same scenario is encountered: the interaction energy of π-hoh-reg is highly underestimated at CE-B3LYP level (1a,1b in Table S5). In this case, there are clearly more differences between interaction energies calculated at QE PBE0 D3 level and estimated with CE-B3LYP since the crystal packing exclusively involve π-stacking interactions with different degrees of latitudinal and longitudinal slippage (2-6 in Table S5). Here, the CE-total energies are both

below and above the corresponding calculated interaction energy. Therefore, for **2·-HT**, in some cases there is an overestimation and in others an underestimation of the interaction between radical units.

Table S5. Intra/inter-synthon interaction energies (in kJ mol^{-1}) of **2·-HT**, as calculated using the explicit DFT/PBE0 functional (E_{int}, QE PBE0 D3 level) or as fitted to the so-called CE-B3LYP procedure. Interactions are listed as the distance between centroid···centroid of radicals increases (in Å, d_{cent} as measured with Mercury[S16]). Note N stands for the number that a given interaction is univocally found given a reference radical **R·**, which is pancaked between 1a and 1b radicals in the below figure.

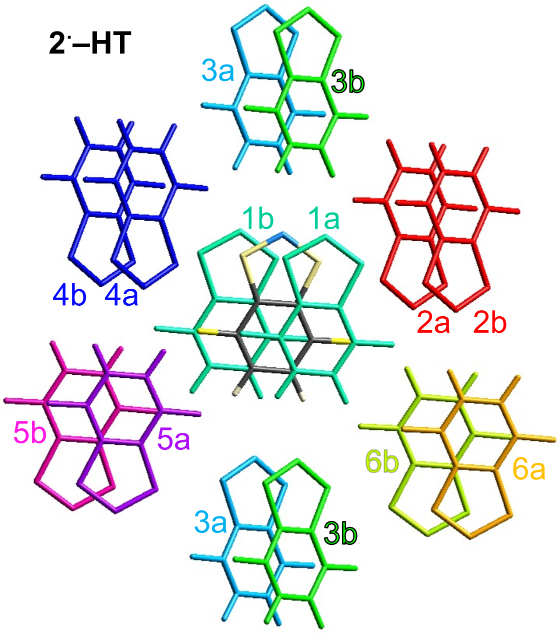

	Mercury					Crystal Explorer					
N	d_{cent}	ref in Fig S63	E_{int}		R	E_{ele}	E_{pol}	E_{dis}	E_{rep}	E_{tot}
2	3.626	π-hoh-reg	-31.68	1a/1b	3.63	-4.2	-0.9	-43.0	38.3	-18.9
1	6.921	i-hot-par-π-lat 1	-9.41	5a	7.84	-5.3	-0.8	-8.5	2.3	-12.2
1	7.392	i-hot-par-π-lat 2	-9.07	6a	8.31	-7.7	-0.9	-7.3	1.9	-14.0
2	7.533	i-htt-tilt-π-lat 1	-8.69	2a/2b	6.81	-1.4	-0.1	-8.3	5.5	-5.3
1	7.640	i-hot-par-π-lat 3	-6.94	5b	8.54	-4.9	-0.5	-4.4	0.3	-9.2
2	7.760	i-hot-tilt-π-lat 2	-8.11	4a/4b	7.00	-1.3	-0.1	-7.2	4.5	-5.0
2	8.131	i-hth-tilt-π-lat 1	-10.49	3a	8.15	-5.1	-1.8	-7.3	2.6	-11.4
2	8.294	i-hth-tilt-π-lat 2	-8.53	3b	8.26	-3.7	-1.2	-5.2	1.2	-8.6
1	8.395	i-hot-par-π-lat 4	-2.59	6b	8.97	–1.0	–0.1	–1.3	0.0	-2.3

d_{cent} (in Å) measured using the cif file data with Mercury

R (in Å) as listed in the energy model output file of Crystal Explorer

Table S6. Intra/inter-synthon interaction energies of **2˙-LT**, as calculated using the explicit DFT/PBE0 functional (E_{int}) or as fitted to the so-called CE-B3LYP procedure. Interactions are listed as the distance between centroid···centroid of radicals increases (d_{cent} as measured with Mercury[S16]). Note N stands for the number that a given interaction is univocally found given a reference radical (see Ref. **R˙** pancaked between 1a and 1b).

2˙–LT

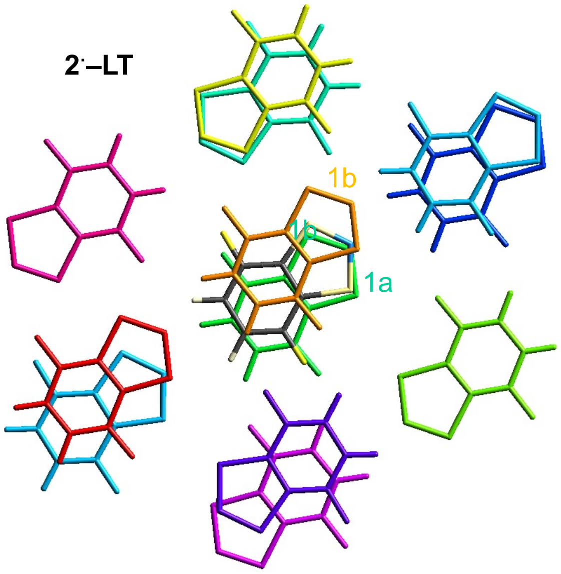

		Mercury				Crystal Explorer					
	N	d_{cent}	ref in Fig S61	E_{int}		R	E_{ele}	E_{pol}	E_{dis}	E_{rep}	E_{tot}
	1	3.411	π-hoh-e	-45.19	1a	3.35	-0.2	-0.1	-0.7	0.0	-0.9
	1	3.696	π-hoh-s	-27.38	1b	3.76	-6.7	-2.1	-7.4	3.6	-12.9
					(1)	6.74	-1.5	-0.1	-8.4	5.4	-5.6
					(1)	6.94	-1.7	-0.1	-1.4	0.0	-3.1
					(1)	6.97	-4.0	-0.8	-12.0	4.5	-12.5
					(1)	7.27	-4.0	-0.8	-12.0	4.5	-12.5
					(0)	7.74	0.0	nan	0.0	0.0	nan
					(2)	8.21	-6.7	-2.1	-7.4	3.6	-12.9
					(1)	8.26	0.0	-0.0	0.0	0.0	-0.0
					(1)	8.27	0.0	-0.0	0.0	0.0	-0.0
					(1)	8.34	0.0	-0.0	0.0	0.0	-0.0

d_{cent} (in Å) measured using the cif file data with Mercury

R (in Å) as listed in the energy model output file of Crystal Explorer

Finally, for **2˙-LT**, there are severe difficulties when it comes to obtain CE-B3LYP energies. As it can be observed in Table S6, the most eclipsed pair of head over head radicals (π-hoh-e, 1a) is not well described, same as the offset slipped π-head over head radical pair (π-hoh-s,

1b). In addition, due to the fact that there are non-equivalent radicals along the π-stack, the resulting interactions are different depending on the radical one chooses as reference. In Table S6, we report one of the possibilities, however, we do not compare any further with the calculated in Figures S61 and S62 (Section S11) because of the obvious problems the CE-total energies have with 1,3,2-benzodithiazolyls π-stacked compounds. Therefore, the figure below only pictures the 1a and 1b pairs along the π-stack.

Our study thus concludes that the estimation of the interaction energy of **R**˙ pairs considering four principal components ($E_{tot} = E_{ele} + E_{pol} + E_{dis} + E_{rep}$) can only provide a reasonable approximation of the SBIs relative strengths for **3**˙ and **2**˙-LT/HT when radicals establish lateral interactions (see Tables S4-S6). The reason for the partial failure of this analysis turns out to be very simple: it is not able to capture the interactions of π-staked radicals. Since interaction energies of π-stacked pairs are underestimated, the dispersion framework is not significative and will not be shown. Note that this will affect the total energy framework as well. Therefore, regrettably, we cannot extrapolate the CrystalExplorer data to draw conclusions for **1**˙ and **4**˙ in terms of interaction energy.

S13. MAGNETIC RESPONSE

The distinct magnetic response of **2**$^{\cdot}$ and **3**$^{\cdot}$ / **4**$^{\cdot}$ is evaluated following a first-principles bottom-up four-step procedure.[S17-S19] Prior to the simulation of their magnetic response, we will proceed to validate the value of J_{AB} calculated at UB3LYP/def2-tzvp level against CASSCF/QD-NEVPT2 level using the same basis set.[S20]

Validation of J_{AB} at QD-NEVPT2 level

For **3**$^{\cdot}$ and **4**$^{\cdot}$, magnetic exchange integrals J_{AB} between **R**$^{\cdot}$s in two selected representative π- and σ-dimers **R**$^{\cdot}_{2}$ extracted from XRD crystal structures at 200 K (Figure S48) are evaluated at broken-symmetry (BS) (U)B3LYP and state-averaged (SA) CASSCF/QD-NEVPT2 levels with the def2-tzvp basis set using *Gaussian*[S21] and *ORCA*[S22] codes, respectively, with the objective of validating magnetic couplings calculated at DFT level against higher-level QD-NEVPT2 calculations. Specifically, the selected π-dimer and σ-dimer are the head-over-tail (π-hot in Figure S48) and coplanar 4-center head-to-tail (σ-4c-htt in Figure S48) pairs that are building blocks of the σ-4c-mediated zip-π-stack synthon of **3**$^{\cdot}$ and **4**$^{\cdot}$.

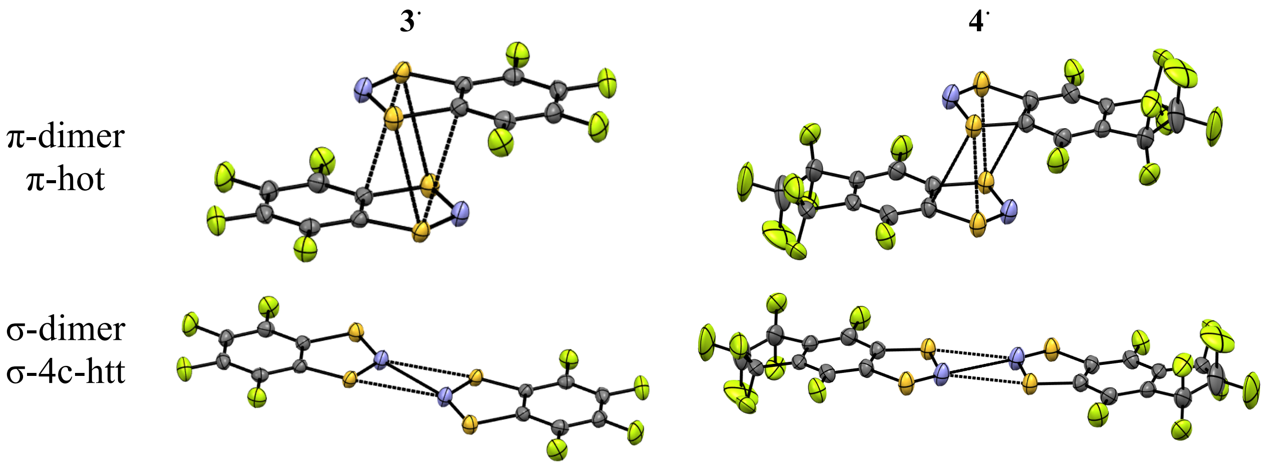

Figure S48. π- and σ-dimers of **3**$^{\cdot}$ and **4**$^{\cdot}$ exhibiting shortened S···S and S···N contacts, respectively, in XRD crystal structures at 200 K.

In the CASSCF calculations, the active space consists of 14 electrons in 12 MOs (see Figures S49-S52). The QD-NEVPT2 calculations are carried out on top of a SA-CASSCF wave function for each conformation of the dimers; MOs are optimized for the average of the lowest-energy singlet and lowest-energy triplet states.

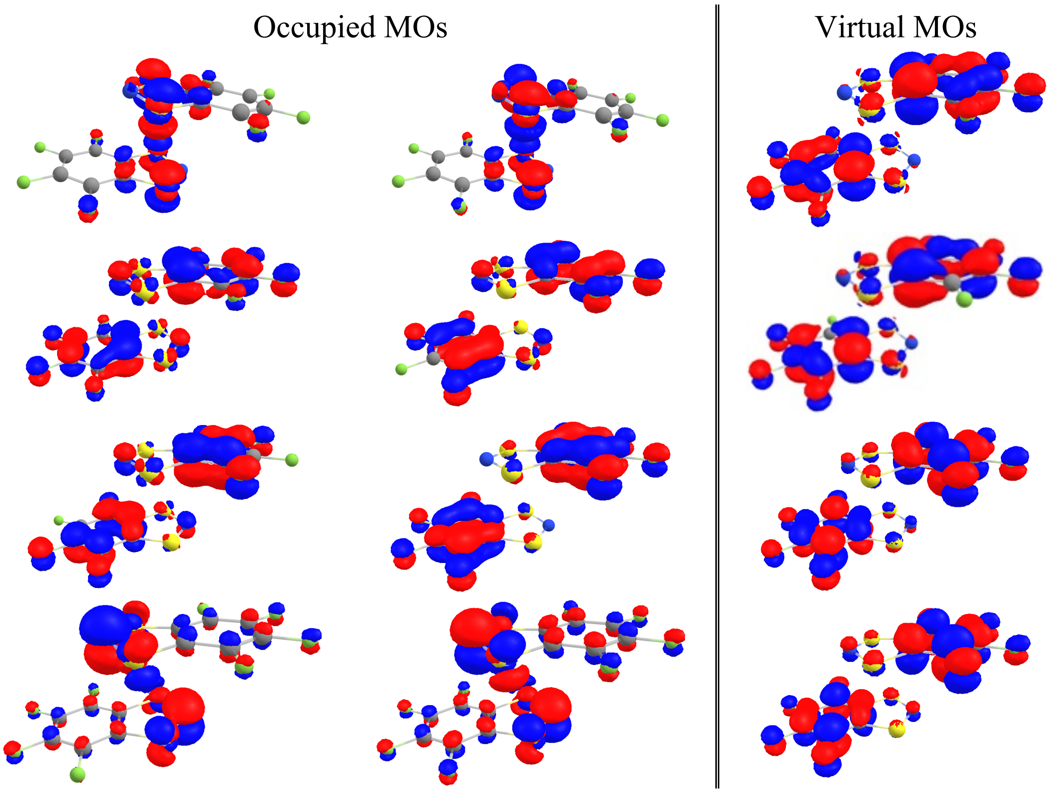

Figure S49. MOs in active space for π-dimer **3**˙$_2$.

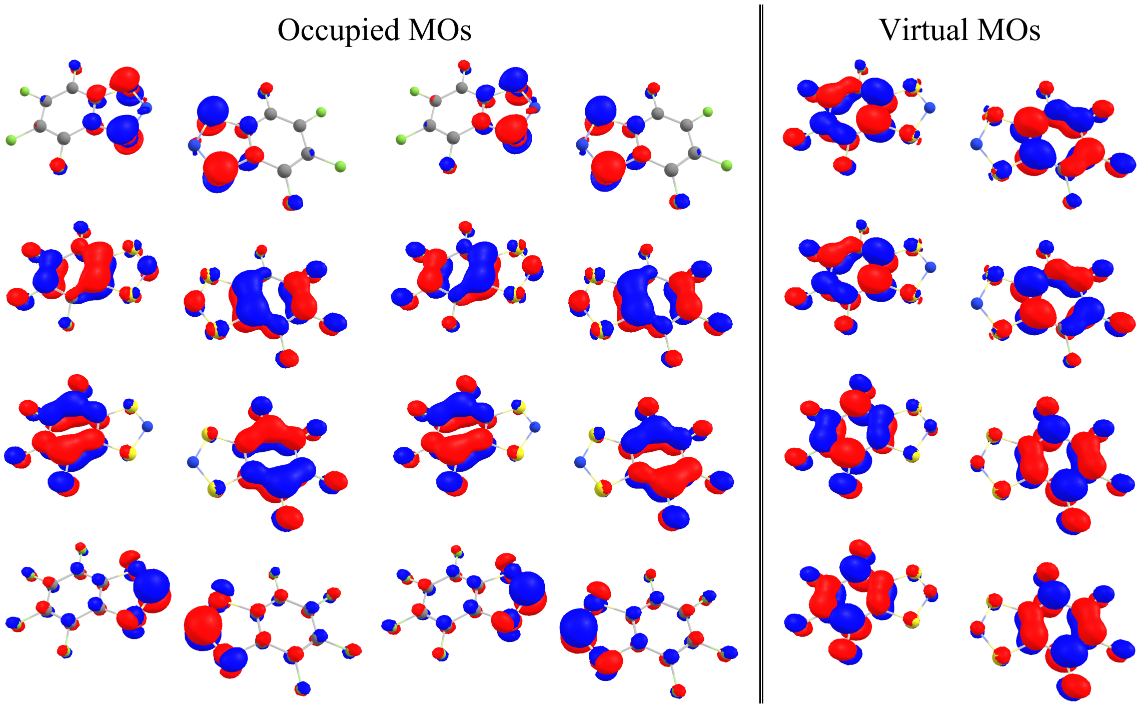

Figure S50. MOs in active space for σ-dimer **3**˙$_2$.

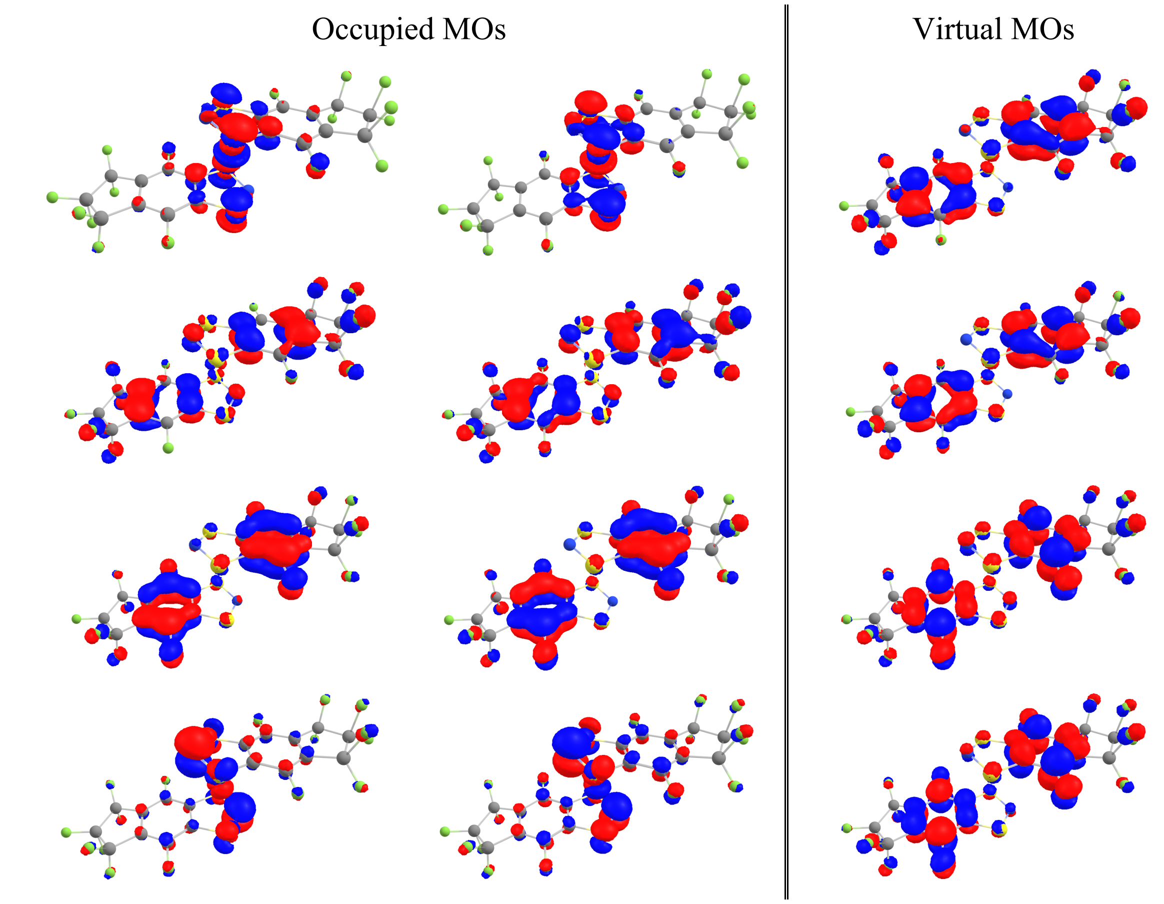

Figure S51. MOs in active space for π-dimer $\mathbf{4}^{\cdot}_{2}$.

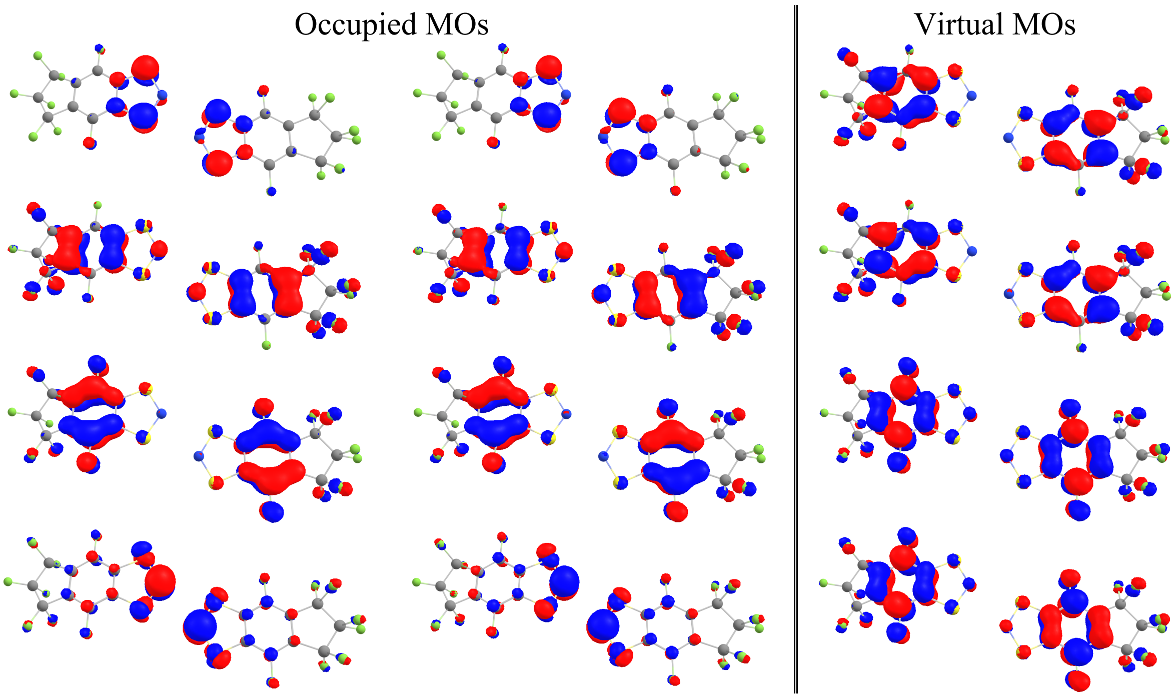

Figure S52. MOs in active space for σ-dimer $\mathbf{4}^{\cdot}_{2}$.

In the SA-CASSCF/QD-NEVPT2 calculations, the integrals J_{AB} were calculated through the lowest singlet-triplet gap ΔE^{S-T} in a pair of neighboring A and B **R**˙s as $\Delta E^{S-T} = E^S - E^T = 2J_{AB}$. In the BS calculations within the Heisenberg Hamiltonian $\hat{H} = -2\sum_{A,B} J_{AB}\, \hat{S}_A \cdot \hat{S}_B$ framework, J_{AB} were calculated both using Yamaguchi and Noodleman approaches. In the former, $J_{AB,Y} = (E_{LS}^{BS} - E_{HS})/(\langle S^2\rangle_{HS} - \langle S^2\rangle_{LS}^{BS})$, where LS / HS stand for low / high spin. In the latter, $J_{AB,N} = (E_S^{BS} - E_T)/(1 + S_{ab}^2)$, where S / T stand for open-shell singlet / triplet states, and S_{ab} is the overlap between *a* and *b* SOMOs belonging to A and B radicals **R**˙s. The Yamaguchi approach is valid over the whole range of coupling strengths converging to the Noodleman approach for weak couplings.[S23-S25]

At SA-CASSCF(14,12) level, the J_{AB} values are markedly lower than for both QD-NEVPT2(14,12) and BS (U)B3LYP ones, as expected since it does not introduce dynamical electron correlation: for π-dimers, J_{AB} is –770 (**R**˙ = **3**˙) and –885 (**R**˙ = **4**˙) cm^{-1}; and for σ-dimers, –1 (**R**˙ = **3**˙) and –14 (**R**˙ = **4**˙) cm^{-1} (Table S7). According to SA-CASSCF(14,12)/QD-NEVPT2 calculations with def2-tzvp basis set, J_{AB} is –1070 (**R**˙ = **3**˙) and –1230 (**R**˙ = **4**˙) cm^{-1} for π-dimers, and –22 (**R**˙ = **3**˙) and –38 (**R**˙ = **4**˙) cm^{-1} for σ-dimers. At BS UB3LYP/def2-tzvp level, the values obtained with both Yamaguchi / Noodleman approaches for σ-dimers are similar, –17 / –17 (**R**˙ = **3**˙) and –36 / –34 (**R**˙ = **4**˙) cm^{-1}. However, $J_{AB,N}$ are more antiferromagnetic for π-dimers (Yamaguchi / Noodleman): J_{AB} is –1431 / –1786 (**R**˙ = **3**˙) and –1595 / –2050 (**R**˙ = **4**˙) cm^{-1}. Although there is a difference in value, Yamaguchi and Noodleman's approaches agree on the magnitude and sign of the magnetic coupling J_{AB}. Due to the large AFM character of the interactions, we do not foresee changes in the magnetic response. Yet, the different J_{AB} will entail a distinct degree of paramagnetic impurities required to reproduce the experimental magnetic behavior of compounds **3**˙ and **4**˙. In the following, we will address this point.

Table S7. Magnetic exchange integrals J_{AB}, cm^{-1}, calculated for π- and σ-dimers of 3˙ and 4˙ (Figure S48)$^{a\text{-}c}$

Dimer	**R**˙	BS (U)B3LYP, code<br>Noodleman ^{a}	 Yamaguchi ^{b}	SA-CASSCF(14,12)^c	QD-NEVPT2(14,12)
π-dimer	**3**˙	–1786	–1431	–770	–1070
π-hot	**4**˙	–2050	–1595	–885	–1229
σ-dimer	**3**˙	–17	–17	–1	–22
σ-4c-htt	**4**˙	–34	–36	–14	–38
			J (**3**˙) / *J* (**4**˙), %		
π-hot		87	90	87	87
σ-4c-htt		50	47	71	58

^{a} def2-tzvp basis set, *Gaussian* code. ^{b} def2-tzvp basis set, *ORCA* code. ^{c} For MOs in active space, see Figures S49-S52.

It is important to note that these values vary significantly upon increasing temperature: at 296 K for **3**˙, the magnetic interaction for the π-dimer decreases to $-1448\ cm^{-1}$ and increases slightly for the σ-dimer to $-18\ cm^{-1}$. Note that Noodleman and Yamaguchi schemes (which assume negligible and non-negligible overlap between SOMOs, respectively[S23-S25]) give consistent results and that, although the numerical value of J_{AB} is different, the ratio J (**3**˙) / J (**4**˙) is preserved in both π- and σ-dimers (Table S7). From this study, one concludes that, despite criticism towards BS,[S24] it is safe to use UB3LYP as a functional to calculate J_{AB} magnetic couplings. Next, we will proceed to systematically evaluate J_{AB} for all possible **R**˙ pairs of **2**˙ and **3**˙, both within and between synthons at BS-DFT/(U)B3LYP level as implemented in the Gaussian code and using Noodleman's scheme. Once more let us stress that compounds **2**˙ and **3**˙ have been chosen as target to be studied because they are representatives of distinct magnetic response: bistability for **2**˙ and diamagnetism for **3**˙.

Simulation of magnetism

The magnetic response of compounds **2**˙ and **3**˙ is studied with a first-principles bottom-up four-step procedure.[S17] First, the crystal packing is analyzed to select pairs of A, B radicals that might be magnetically important, in terms of **R**˙···**R**˙ distances. This has been already done when assessing the interaction energy E_{int} between a reference radical and its surrounding congeners (see SI Section S11). Secondly, once all those A···B dimers that are univocally defined within the crystal have been identified, the microscopic J_{AB} radical···radical magnetic interactions are computed. Systematic evaluation of J_{AB} for all possible pairs of radicals within the synthon and between synthons results in very few significative magnetic couplings (see Table S8). Once all J_{AB}'s have been evaluated, the magnetic topology can be next defined in terms of all computed non-negligible J_{AB} magnetic coupling interactions. From the magnetic topology, we select a representative magnetic model (see Figure S53) to solve the secular equation problem together with the energy spectra and corresponding spin quantum numbers.

There is only one non-negligible magnetic coupling for **2**˙-HT, namely, $J_{\pi-hoh-reg}^{\mathbf{2}^{\cdot}-\mathrm{HT}} = -484.9\ cm^{-1}$, that gives rise to a magnetic topology of isolated regular AFM 1D π-stacks of radicals (Figure S53a). For **2**˙-LT, the magnetic topology consists of alternant AFM 1D π-stacks ($J_{\pi-hoh-e}^{\mathbf{2}^{\cdot}-\mathrm{LT}} = -2356.6\ cm^{-1}$ and $J_{\pi-hoh-s}^{\mathbf{2}^{\cdot}-\mathrm{LT}} = -31.9\ cm^{-1}$ in red and green, respectively) which then interact among then very weakly ($J_{i-hoh-par-\pi 1}^{\mathbf{2}^{\cdot}-\mathrm{LT}} = +0.03\ cm^{-1}$ and $J_{co-htt-lat2}^{\mathbf{2}^{\cdot}-\mathrm{LT}} = -0.01\ cm^{-1}$) (Figure S53b). Therefore, the π-stacks can, in practice, be considered effectively to be isolated as in **2**˙-HT. Please note that the radicals interacting to generate the magnetic topology are those involved in the crystal packing synthons of the HT and LT phases of **2**˙ (SI Section S11). The

magnetic model for both **2**˙-HT and **2**˙-LT is thus formed by cyclic 1D chain models, as illustrated in Figure S53a and S53b, respectively.

Table S8. Magnetic couplings J_{AB} (in cm^{-1}) for significant radical pair interactions obtained at (U)B3LYP/def2-tzvp level using Noodleman's scheme.

**R˙**	J_{AB}, **within synthon**			J_{AB}, **between synthons**	
**2**˙-HT	π-hoh-reg				
	-484.9				
**2**˙-LT	π-hoh-e	π-hoh-s		i-hoh-par-π 1	co-htt-lat 2
	-2356.6	-31.9		+0.03	-0.01
**3**˙	π-hot	π-hoh	σ-4c-htt	orthogonal 2	
(200K)	-1786.0	-9.2	-17.3	+0.60	
(296K)	-1447.7	-7.4	-18.3	+0.52	
**4**˙	π-hot	π-hoh	σ-4c-htt	orthogonal 2	
	-2050.3	-4.0	-33.7	-4.02	

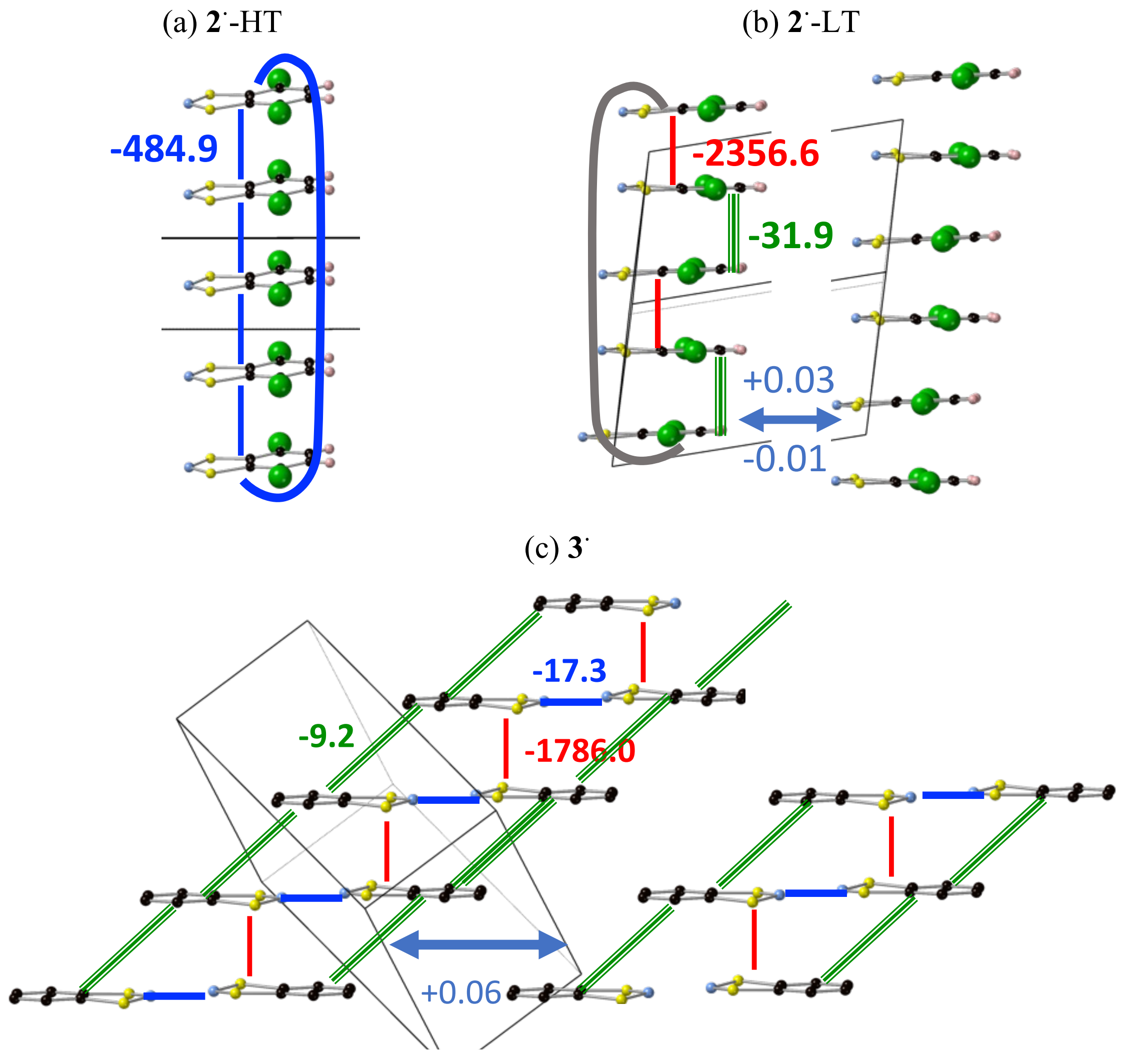

Figure S53. Magnetic models for (a) **2**˙-HT, (b) **2**˙-LT and (c) **3**˙.

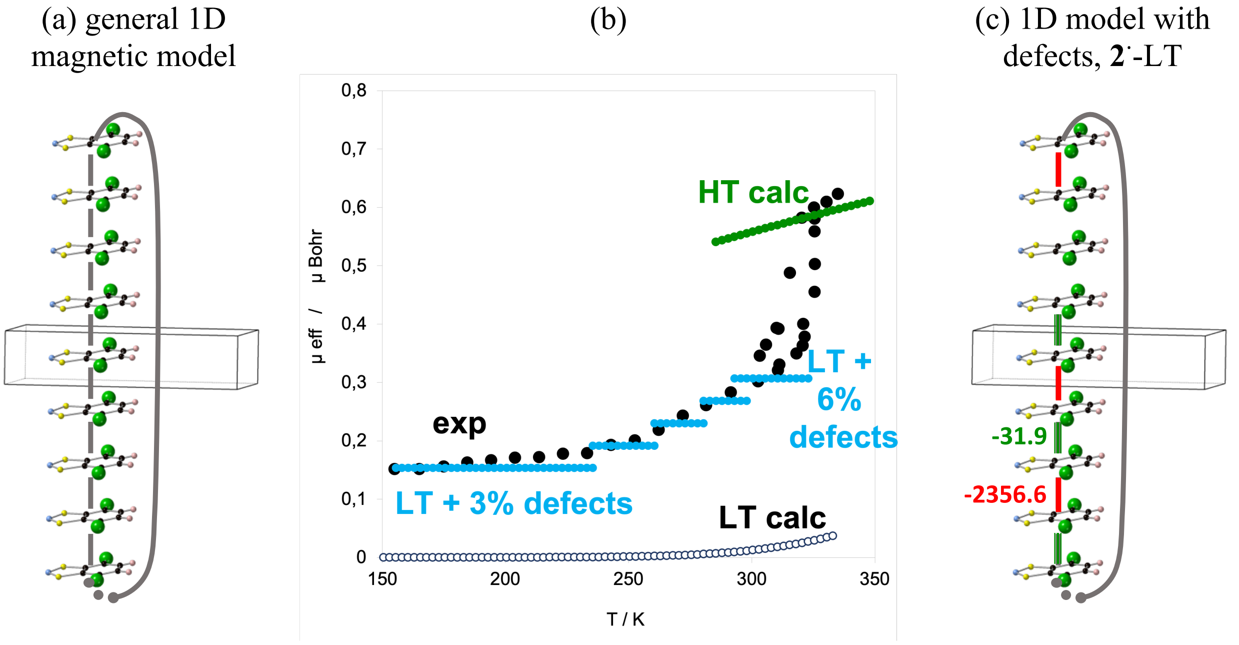

Figure S54. (a) General 1D magnetic model for both HT and LT phases of **2**$^{\cdot}$. (b) Magnetic response of **2**$^{\cdot}$: experimental data, in black; simulated values using either HT or LT data in green and empty black, respectively; simulated values introducing paramagnetic defects to LT data ranging from 3% to 6%, in light blue. Introduction of one single paramagnetic defect (there is one uncoupled radical) into the (c) 1D π-stack magnetic model of **2**$^{\cdot}$-LT.

In **3**$^{\cdot}$, the magnetic topology at 200 K involves AFM strong-rail spin ladders with two distinct rail interactions, namely $J_{\pi-hot}^{\mathbf{3}^{\cdot}}$= –1786.0 and $J_{\pi-hoh}^{\mathbf{3}^{\cdot}}$= –9.2 cm^{-1}, and with $J_{\sigma 4c-htt}^{\mathbf{3}^{\cdot}}$= –17.3 cm^{-1} rung that interact ferromagnetically with nearby spin ladders by $J_{orthogonal2}^{\mathbf{3}^{\cdot}}$= +0.06 cm^{-1} (Figure S53c). Magnetic models with and without inter-spin-ladder interactions have been used to simulate the magnetic response, and no significant change is observed upon inclusion. The magnetic model of **3**$^{\cdot}$, as also observed in **2**$^{\cdot}$-HT and **2**$^{\cdot}$-LT, aligns with the structural synthon of **3**$^{\cdot}$.

The experimental magnetic susceptibility χ(T) data was finally reproduced using cyclic AFM chain (16 radical) models for **2**$^{\cdot}$-LT and **2**$^{\cdot}$-HT with magnetic couplings as listed in Table S5 (see Figure S54a for general magnetic model). At high temperatures, the agreement between experimental (in black) and simulated data from **2**$^{\cdot}$-HT (in green, Figure S54b) is good. However, at low temperatures, it is not: our simulations picture a magnetically silent **2**$^{\cdot}$-LT compound (see empty black symbols, Figure S54b). Note that, for **2**$^{\cdot}$, it has been already indicated that to reproduce the experimental data a value of $J_{\pi-hoh-e}^{\mathbf{2}^{\cdot}-\mathrm{LT}} \approx$ –500 cm^{-1} was required, and that μ_{eff} reaches values corresponding to 7% of the paramagnetic state at 300 K. These pieces of evidence have fueled us to study the potential presence of uncoupled radicals in the crystalline sample. We proceeded to simulate the existence of radicals within the cyclic AFM chain model by not letting

one radical of **2**˙-LT antiferromagnetically couple to adjacent neighbor radicals. Therefore, one $J_{\pi-hoh-e}^{2^{\cdot}-LT}$ and one $J_{\pi-hoh-s}^{2^{\cdot}-LT}$ magnetic couplings must be removed to create one single paramagnetic defect (see Figure S54c). The contribution of those uncoupled radicals (see blue symbols in Figure S54b) rationalizes quantitatively the experimental magnetic response of the LT phase of **2**˙. Specifically, below 240 K, a 3% of paramagnetic defects is sufficient to reproduce the experimental μ_{eff} data. This percentage rises to 6% around 330 K.

(a) σ-4c mediated zip-π-stack with defects, **3**˙

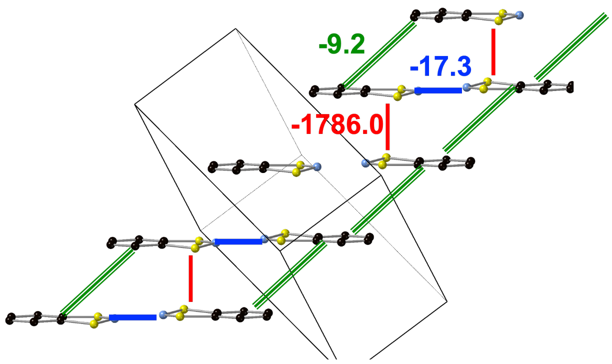

(b)

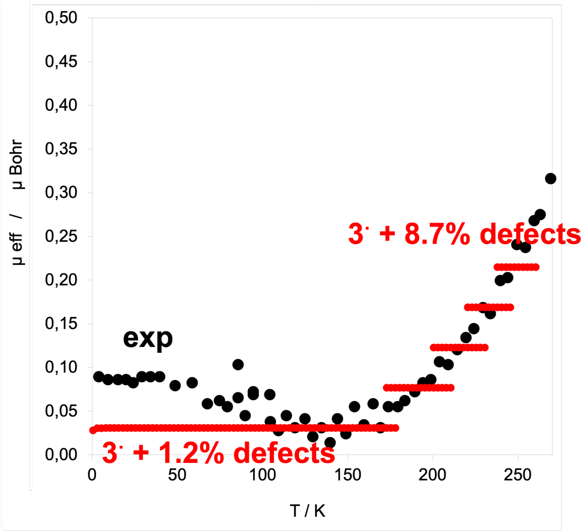

(c) σ-4c mediated zip-π-stack with defects, **4**˙

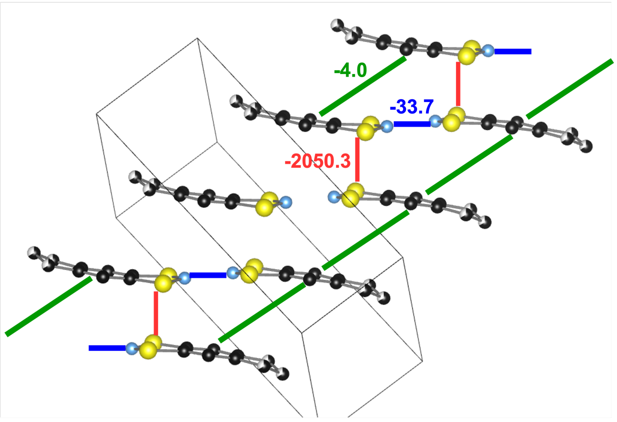

(d)

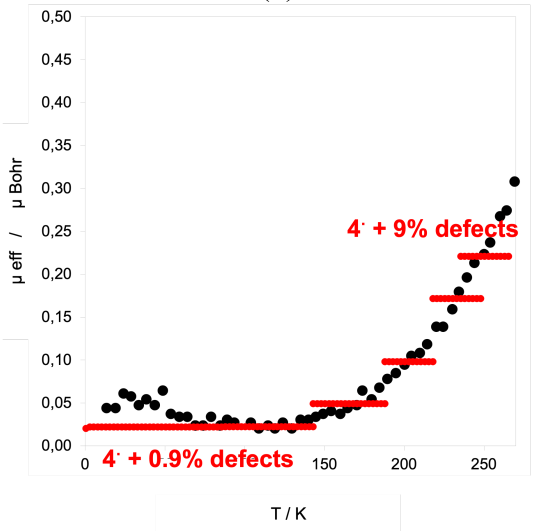

Figure S55. Magnetic model based on σ-4c-mediated zip-π-stack synthon of (a) **3**˙ and (c) **4**˙. Magnetic response of (b) **3**˙: experimental data, in black; simulated values introducing paramagnetic defects to **3**˙ data ranging from 1.2% to 8.7%, in red; (d) **4**˙: experimental data, in black; simulated values introducing paramagnetic defects to **4**˙ data ranging from 0.9% to 9.0%, in red.

For compounds **3**˙ and **4**˙, estimation of the fraction of the paramagnetic state (ω_{HS}) provided the number of paramagnetic defects to be ~17% for **3**˙ and ~22% for **4**˙ (SI Section S9). Therefore, simulations accounting for paramagnetic defects have also been carried out to reproduce the experimental magnetic response. Note that, in the case of **3**˙, one $J_{\pi-hot}^{\mathbf{3}^{\cdot}}$, two $J_{\pi-hoh}^{\mathbf{3}^{\cdot}}$ and one $J_{\sigma 4c-htt}^{\mathbf{3}^{\cdot}}$ magnetic coupling interactions must be removed to create one paramagnetic defect, see Figure S55a. The same applies to **4**˙ (see Figure S55c). Our results indicate that the presence of unpaired radicals reproduces the experimental magnetic response (see Figures S55b and S55d). Simulated values introducing paramagnetic defects to **3**˙ data range from 1.2% to 8.7% and vary depending on the temperature. Same happens for **4**˙, introducing paramagnetic defects ranging from 0.9% to 9.0% reproduces the experimental data. This is a clear indication that thermal fluctuations play an important structural role that should be incorporated into simulations. However, this is currently out of the scope of this paper.

S14. REFERENCES AND NOTES

(S1) I. Yu. Bagryanskaya, H. Bock, Yu. V. Gatilov, A. Haas, M. M. Shakirov, B. Solouki, A. V. Zibarev, *Chem. Ber. / Recueil* **1997**, *130*, 247.

(S2) M. L. Laury, S. E. Boesch, I. Haken, P. Sinha, R. A. Wheeler, A. K. Wilson, *J. Comput. Chem.* **2011**, *32*, 2339.

(S3) A. D. Laurent, D. Jacquemin, *Quant. Chem.* **2013**, *113*, 2019.

(S4) J. Tomasi, B. Mennucci, R. Cammi, *Chem. Rev.* **2005**, *105*, 2999.

(S5) I. Sandler, J. Chen, M. Taylor, S. Sharma, J. Ho, *J. Phys. Chem. A* **2021**, *125*, 1553.

(S6) A. Yu. Makarov, A. A. Buravlev, G. V. Romanenko, A. S. Bogomyakov, B. A. Zakharov, V. A. Morozov, A. S. Sukhikh, I. K. Shundrina, L. A. Shundrin, I. G. Irtegova, S. V. Cherepanova, I. Yu. Bagryanskaya, P. V. Nikulshin, A. V. Zibarev, *ChemPlusChem* **2024**, *89*, e202300736.

(S7) The double integral of the etalon EPR signal was assigned to the number of spins in the etalon. Since the Q-factor of the resonator does not change significantly in the studied temperature range 220 < T < 330 K, the temperature dependence of the observed double integrals relates to the corresponding temperature change in N_T (see Figure S50c).

(S8) M. M. Haugland, J. E. Lovett, E. A. Anderson, *Chem. Soc. Rev.* **2018**, *47*, 668.

(S9) G. A. Bain, J. F. Berry, *J. Chem. Educ.* **2008**, *85*, 532.

(S10) G. D. McManus, J. M. Rawson, N. Feeder, J. van Duijn, E. J. L. McInnes, J. J. Novoa, R. Burriel, F. Palacio, P. Oliete, *J. Mater. Chem*. **2001**, *11*, 1992.

(S11) P. Giannozzi, et al. *J. Phys. Condens. Matter* **2017**, *29*, 465901.

(S12) P. R. Spackman, M. J. Turner, J. J. McKinnon, S. K. Wolff, D. J. Grimwood, D. Jayatilaka, M. A. Spackman, *J. Appl. Crystallogr.* **2021**, *54*, 1006.

(S13) S. L. Tan, M. M. Jotani, E. R. T. Tiekink, *Acta Crystallogr. E* **2019**, 75, 308.

(S14) T. Lu, *Angew. Chem. Int. Ed.* **2025**, *64*, e202504895.

(S15) C. F. Mackenzie, P. R. Spackman, D. Jayatilaka, M. A. Spackman, *IUCrJ* **2017**, *4*, 575

(S16) C. F. Macrae, P. R. Edgington, P. McCabe, E. Pidcock, G. P. Shields, R. Taylor, M. Towler, J. van de Streek, *J. Appl. Crystallogr.* **2006**, *39*, 453.

(S17) M. Deumal, M. J. Bearpark, J. J. Novoa, M. A. Robb, *J. Phys. Chem. A* **2002**, *106*, 1299.

(S18) J. J. Novoa, M. Deumal, J. Jornet-Somoza, *Chem. Soc. Rev.* **2011**, *40*, 3182.

(S19) J. Jornet, M. A. Robb, M. Deumal, J. J. Novoa, *Inorg. Chim. Acta* **2008**, *361*, 3586.

(S20) M. Fumanal, J. Jornet-Somoza, S. Vela, J. J. Novoa, J. Ribas-Ariño, M. Deumal, *J. Mater. Chem. C* **2021**, *9*, 10647.

(S21) M. J. Frisch, et al. *Gaussian 09*; Gaussian Inc., Wallingford, CT; 2009.

(S22) F. Neese, *WIREs Comp. Mol. Sci.* **2022**, *12*, e1606.

(S23) J. P. Malrieu, R. Caballol, C. J. Calzado, C. de Graaf, N. Guihèry, *Chem. Rev.* **2014**, *114*, 429.

(S24) N. Iwara, Z. Huang, A. Mansikkamäki, L. F. Chibotaru, *J. Phys. Chem.* **2025**, *162*, 164701.

(S25) F. Neese, *J. Phys. Chem. Solids* **2004**, *65*, 781.
